# Speciation genes are more likely to have discordant gene trees

**DOI:** 10.1002/evl3.77

**Published:** 2018-08-08

**Authors:** Richard J. Wang, Matthew W. Hahn

**Affiliations:** ^1^ Department of Biology Indiana University Bloomington Indiana; ^2^ Department of Computer Science Indiana University Bloomington Indiana

**Keywords:** Dobzhansky–Muller incompatibilities, gene tree discordance, incomplete lineage sorting, reproductive isolation, speciation

## Abstract

Speciation genes are responsible for reproductive isolation between species. By directly participating in the process of speciation, the genealogies of isolating loci have been thought to more faithfully represent species trees. The unique properties of speciation genes may provide valuable evolutionary insights and help determine the true history of species divergence. Here, we formally analyze whether genealogies from loci participating in Dobzhansky–Muller (DM) incompatibilities are more likely to be concordant with the species tree under incomplete lineage sorting (ILS). Individual loci differ stochastically from the true history of divergence with a predictable frequency due to ILS, and these expectations—combined with the DM model of intrinsic reproductive isolation from epistatic interactions—can be used to examine the probability of concordance at isolating loci. Contrary to existing verbal models, we find that reproductively isolating loci that follow the DM model are often more likely to have discordant gene trees. These results are dependent on the pattern of isolation observed between three species, the time between speciation events, and the time since the last speciation event. Results supporting a higher probability of discordance are found for both derived–derived and derived–ancestral DM pairs, and regardless of whether incompatibilities are allowed or prohibited from segregating in the same population. Our overall results suggest that DM loci are unlikely to be especially useful for reconstructing species relationships, even in the presence of gene flow between incipient species, and may in fact be positively misleading.

**Impact Summary**

The variety of species in nature is kept distinct by the barriers that prevent interbreeding. New species form when genetic changes within different populations prevent them from reproducing. The genetic analysis of reproductive incompatibilities has revealed the identity of genes responsible for reproductive isolation—and thus, speciation—in a number of species. These genes may have a unique pattern of evolution because of their participation in the process of speciation. It has been hypothesized that the evolutionary histories of speciation genes could be especially useful for determining the order in which species diverged. In this article, we formally analyze this hypothesis by combining the prevailing genetic model of speciation with population genetic theory. We find that genetic loci responsible for reproductive isolation do have a unique signal, but in a way that can often be misleading about the order in which species diverged. Our findings contradict existing models and provide a new expectation for the evolutionary history of speciation genes.

Speciation proceeds from the evolution of reproductive isolation between populations. The study of reproductive isolation has advanced our understanding of the genetic basis of speciation for which a common evolutionary model has become established. The Dobzhansky–Muller (DM) model describes how hybrid incompatibilities can arise as the result of epistasis between two or more loci that have diverged between populations (Bateson 1909; Dobzhansky and Dobzhansky 1937; Muller 1942). By having incompatible alleles for these loci arise in separate populations, the DM model allows reproductive isolation to evolve between populations without the appearance of reproductive failure within populations. A growing number of so‐called “speciation genes” that isolate species in accordance with the DM model have emerged from the genetic analysis of reproductive isolation in hybrids (e.g., Ting et al. 1998; Barbash et al. 2003; Presgraves et al. 2003; Bomblies and Weigel 2007; Mihola et al. 2009; Phadnis and Orr 2009; Barr and Fishman 2010; Lienard et al. 2016). Combinations of alleles from different species at these genes cause hybrid infertility or inviability.

The identification of speciation genes in multiple model systems has led to a search for the genetic, molecular, and evolutionary commonalities among them (Orr et al. 2004; Wu and Ting 2004; Oliver et al. 2009; Presgraves 2010; Rieseberg and Blackman 2010; Nosil and Schluter 2011; Castillo and Barbash 2017). A major question is whether the genes leading to reproductive isolation differ from other genes in the genome. Various hypotheses have suggested that speciation genes are more likely to be the targets of adaptive evolution (Coyne and Orr 2004), more prone to interact with other genes (Guerrero et al. 2017), or more likely to be involved in genetic conflict (Bomblies and Weigel 2007; Phadnis and Orr 2009; Ågren 2013).

The unique role of speciation genes in establishing species boundaries has also led to arguments asserting that these genes should be especially informative about species relationships (Ting et al. 2000; Rosenberg 2003; Maroja et al. 2009; Zachos 2009; Nosil and Schluter 2011; Cutter 2013). Such a property becomes useful when multiple species are separated by very short times between successive speciation events. In these cases, individual gene trees may have different topologies from one another and from the species tree (Maddison 1997). This phenomenon is not due to low power or sampling error, but represents a real difference in the genealogical history between loci, due to incomplete lineage sorting (ILS) or gene flow. With the high degree of discordance seen in many systems (e.g., Pollard et al. 2006; White et al. 2009; Jarvis et al. 2014; Pease et al. 2016), concordance between the topology of speciation genes and species trees would provide uniquely powerful insight into evolutionary histories. Verbal models have created the impression that speciation loci are biased toward concordance (Ting et al. 2000; Nosil and Schluter 2011; Cutter 2013), but no formal analysis of this idea has been carried out.

Here, we compare the expected genealogical history of loci involved in Dobzhansky‐Muller Incompatibilities (DMIs) to the expected history of loci uninvolved in incompatibilities from the same genomes. The appreciation of discordance among gene trees has become acute with whole‐genome sequence data, leading to multiple methods that incorporate ILS in the inference of species trees (e.g., Liu et al. 2009; Larget et al. 2010; Drummond et al. 2012; Mirarab and Warnow 2015). However, gene tree discordance has received limited consideration in the context of DMIs. Loci involved in DMIs present additional challenges because of the epistatic nature of incompatibilities, which means that participating loci must act together to produce the incompatible phenotype. In addition, because both alleles involved in an incompatibility cannot segregate in the same population without leading to lower fitness in some individuals, the order in which mutations arise at each locus in a DMI matters.

We find that under a neutral model with ILS, the stochastic processes of mutation and coalescence typically lead to higher rates of species tree discordance at hybrid incompatibility loci. We arrive at this counterintuitive result by examining the probability of ILS at loci participating in a canonical two‐locus DMI. Our analysis considers four potential types of gene trees at a hypothetical incompatibility locus (Fig. 1). A key initial insight is recognizing the possibility that incompatible alleles can arise on discordant gene trees and still lead to reproductive isolation between pairs of species. Figure 2 shows how a DMI can arise from two loci, both with discordant gene trees, and isolate one or more species pairs. Because the expected branch lengths for each type of gene tree differ, mutations giving rise to incompatible alleles are not equally likely among the types of gene trees. We consider each combination of topologies for a pair of loci and calculate the probability of a DMI from differences in expected branch lengths. We find that loci participating in DMIs are typically more likely to have discordant gene trees, and that some patterns of isolation between species are more likely when loci are discordant.

**Figure 1:**
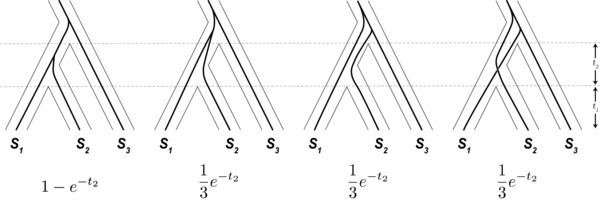
Four types of gene trees at a DMI locus and their expected frequencies. Two gene trees concordant with the species tree (left), and two that are discordant (right). Only the leftmost gene tree coalesces before the first speciation event. The labeled times, *t*_1_ and *t*_2_, are the time from present to the first speciation event and the time between speciation events, respectively. Below each gene tree is the probability of its occurrence for a random locus in the genome under ILS alone.

**Figure 2:**
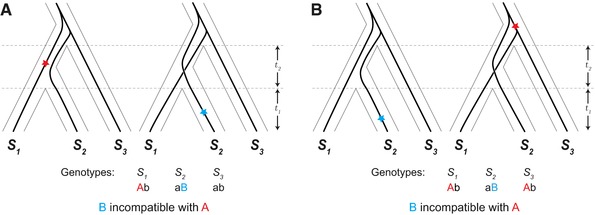
Incompatibility between one or more species pairs due to alleles from two loci that both have discordant gene trees, (*S*_2_, *S*_3_) *S*_1_ and (*S*_1_, *S*_3_) *S*_2_. Red and blue stars mark the position of the first and second mutations, respectively, that produce incompatible alleles. The ancestral genotype for the two loci is denoted “ab”, with the mutations producing derived alleles “A” and “B”. (A) Incompatibility between lineages *S*_1_ and *S*_2_. (B) Incompatibility between lineages *S*_1_ and *S*_2_ as well as *S*_3_ and *S*_2_ from shared incompatible alleles.

## Results

### PRELIMINARIES

**Figure 3:**
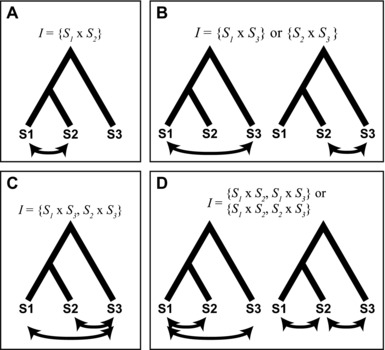
Four patterns of reproductive isolation. A single pairwise DMI can isolate a pair of species, as in panels (A) and (B), or two pairs of species, as in panels (C) and (D). For subsequent calculations, *S*_1_ and *S*_2_ are often interchangeable, leading us to group the two sets of relationships in (B) and (D).

Our genealogical model considers a single pairwise DMI in a three‐species complex. DMIs are typically modeled as isolating two taxa, but depending on where an incompatible allele arises on a phylogenetic tree, a DMI can be shared among different species pairs (Moyle and Payseur 2009). The most straightforward way for this to occur is to have an interaction between two derived alleles (“derived–derived” incompatibilities; Orr 1995), where one of the derived alleles is shared between species, having arisen before their divergence (Fig. 2B). Two mutations inherited by the same lineage can also result in shared isolation, with the second derived allele being incompatible with the ancestral allele in other taxa (“derived–ancestral” incompatibilities; Orr 1995; Fig. S1). Generally, a DMI involving only two loci can produce six patterns of isolation among three taxa. The topology and branch lengths for the two most recently diverged taxa are interchangeable in many of the subsequent calculations, leaving four unique patterns of reproductive isolation (Fig. 3). As we show below, these patterns of reproductive isolation are more often associated with particular types of gene trees.

We allow loci participating in an incompatibility to be discordant with the species tree, defined by the historical timing and order of species’ divergence, only through ILS. Specifically, a DMI locus can have one of four potential types of gene trees (Fig. 1). Although there are only three potential topologies for three species, we divide concordant gene trees into those that coalesce in the ancestral population and those that coalesce between the speciation events (i.e., are lineage‐sorted). Discordant gene trees must coalesce in the ancestral population of all three species. For a single locus, each of the three ancestrally coalescing gene trees are equally likely, at $\frac{1}{3}e^{-t_{2}}$, where *t*_2_ is the interspeciation time (Hudson 1983; Nei 1986). This leaves the probability of a concordant, lineage‐sorted gene tree at $1-e^{-t_{2}}$. The more familiar probability for gene‐tree/species‐tree concordance, $1-\frac{2}{3}e^{-t_{2}}$ (Hudson 1983), includes another $\frac{1}{3}e^{-t_{2}}$ from concordant trees that coalesce in the ancestral population of all three species. For two independent loci, the joint probability of any particular pair of genealogies is a product of the individual probabilities. However, this is not the case for two loci participating in a DMI.

### CALCULATING GENE‐TREE/SPECIES‐TREE CONCORDANCE AT A DMI LOCUS

For a given pattern of reproductive isolation, certain pairs of gene trees are more likely to give rise to incompatible alleles. Loci participating in a DMI must have experienced mutations on the appropriate branches to form the corresponding pattern of isolation. As an obvious example, a mutation specific to the *S*_3_ lineage, on any of the gene trees shown in Figure 1, cannot participate in an incompatibility that isolates *S*_1_ from *S*_2_. In the standard coalescent model, the probability of a mutation on a given branch is proportional to its length and independent of the coalescent process. Because branch lengths differ among gene trees, the probability of a DMI depends on the types of gene trees at a pair of loci. Conversely, the probability of a specific pair of gene trees at the two loci involved in a DMI (DMI loci) depends on the pattern of isolation. We can express the relationship between the probability of each pair of gene trees and the probability of an incompatibility through Bayes’ theorem.

Let *I* be the species pair(s) for which a DMI manifests—that is, *I* specifies the pattern of reproductive isolation between species for a DMI (Fig. 3). The probability that a pair of DMI loci have gene trees of type *T_x_* and *T_y_*, respectively, can be expressed as:

(1) $$P\left(T_{x},T_{y}|I\right)\;=\frac{P\;\left(I|T_{x},T_{y}\right)P\;\left(T_{x}\right)P\;\left(T_{y}\right)}{\sum_{n,m}P\left(I|T_{n},T_{m}\right)P\;\left(T_{n}\right)P\;\left(T_{m}\right)}\;,$$

where *n* and *m* are each indices enumerating the four types of gene trees as ordered in Figure 1 (i.e., *T*_1_ and *T*_2_ are concordant, whereas *T*_3_ and *T*_4_ are discordant), and *P*(*T_x_*)*P*(*T_y_*) is the joint probability of *T_x_* and *T_y_* assuming independence as described above.

Assuming incompatibilities are rare between any given pair of loci, the conditional probability *P*(*I*|*T_x_*, *T_y_*) can be written as a sum of the probability that two mutations result in an incompatibility, across all branches of *T_x_* and *T_y_*. Let *x*_α_ and *y*_β_ be indexed branches on trees *T_x_* and *T_y_*, then,

(2) PI|Tx,Ty=p∑α,βP mutation  on xαP mutation  on yβ×1(xα,yβ),

where **1**(*x*_α_, *y*_β_) is 1 when two mutations, on branches *x*_α_ and *y*_β_, can generate a DMI with isolation pattern *I*, and 0 otherwise; and *p* is the probability of an incompatibility forming between untested allelic combinations (Orr 1995; Orr and Turelli 2001). This probability is valid when the mutations are independent, but the order in which mutations occur must be considered for derived‐ancestral incompatibilities (see Methods). The probability of at least one mutation on a given branch can be estimated by its length,

(3) P mutation  on xα≈2Neμ·Lxα,

where 2*N*_e_μ is the population mutation parameter and *L*(*x*_α_) denotes the branch length of *x*_α_ (see Supporting Information Methods and Hudson 1992). Under an infinite sites model, the expression in equation (3) is equivalent to the probability of observing a derived allele. The following calculations assume such a model, with the probability of a derived allele on branches *x*_α_ and *y*_β_ calculated from their respective branch lengths.

With the probability of each pair of genealogies for a given pattern of reproductive isolation, we can calculate the probability that the gene tree for a single DMI locus is concordant with the species tree by summing the marginal probabilities for a concordant topology,

(4) P concordance |I=12∑C,mPTC,Tm|I+12∑n,CPTn,TC|I,

where *n* and *m* are indices over the four types of gene trees as before, and *C* includes only the indices for the concordant trees (i.e., *T*_1_ and *T*_2_). The probability of discordance can similarly be calculated from a sum of marginal probabilities.

### MUTATIONS THAT ISOLATE SISTER TAXA VIA TWO LOCI WITH DISCORDANT GENE TREES

**Figure 4:**
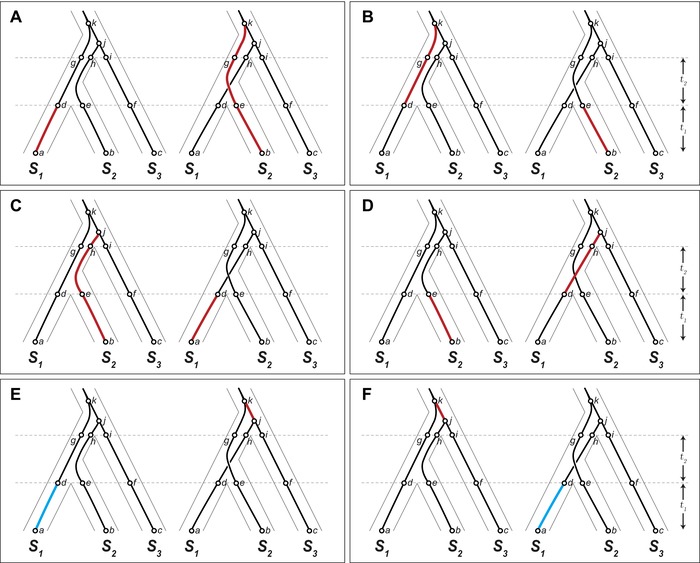
Branch segments on the discordant gene trees, (*S*_2_, *S*_3_) *S*_1_ and (*S*_1_, *S*_3_) *S*_2_, that can give rise to incompatible alleles isolating *S*_1_ and *S*_2_. In (A)–(D), a mutation on the left‐hand tree, (*S*_2_, *S*_3_) *S*_1_, and a mutation on the right‐hand tree, (*S*_1_, *S*_3_) *S*_2_, can give rise to a derived–derived incompatibility. These panels show all combinations of branch segments on which mutations could give rise to a derived–derived incompatibility on the pair of discordant trees shown. Panels (E) and (F) show all combinations of branch segments on which mutations could give rise to a derived‐ancestral incompatibility on this pair of trees. A mutation on branch segment *j*–*k* (red) leaves *S*_2_ with an ancestral allele that can be incompatible with a derived allele produced by a mutation on branch segment *a*–*d* (blue) and inherited by *S*_1_ (see main text).

Determining the limited branch segments on which incompatible alleles for a particular pattern of isolation can arise is central to the calculation of the conditional probability in equation (2). In Figure 4, we illustrate the branch segments on which an incompatible allele isolating the sister taxa, *S*_1_ × *S*_2_, can arise on two discordant gene trees, (*S*_2_, *S*_3_) *S*_1_ and (*S*_1_, *S*_3_) *S*_2_. (For the segments on all gene tree pairs, see Appendix 1 in Supporting Information.) We divide segments on the gene trees in Figure 4 by speciation and coalescent events, labeling each segment by its endpoints (e.g., *a–d* describes the segment specific to *S*_1_ from the present to the most recent speciation event). Two mutations on the highlighted segments in each pair of gene trees in Figure 4, one on the left‐hand tree and one on the right‐hand tree, can produce alleles isolating *S*_1_ and *S*_2_. For example, Figure 4A shows the potential for an *S*_1_ × *S*_2_ incompatibility from a derived allele that arises on the *a–d* segment of the left‐hand tree (inherited by *S*_1_) and a derived allele that arises on segments *b–e*, *e–g*, or *g–k* (inherited by *S*_2_). Because we do not allow incompatibilities to arise before *S*_1_ and *S*_2_ diverge, one mutation must occur on a segment after divergence (i.e., a pair of incompatible alleles cannot arise, for instance, along segment *d–g* on the left‐hand tree and *e–g* on the right‐hand tree).

Interestingly, loci with discordant gene trees can also produce derived–ancestral incompatibilities between sister taxa (Fig. 4E and F). In Figure 4E, a mutation on segment *j–k* on the right‐hand tree produces a derived allele inherited by *S*_1_ and *S*_3_. The second derived allele on segment *a–d* on the left‐hand tree is inherited by *S*_1_, but arises in the background of the derived allele from the first mutation, creating the potential for an incompatibility with the ancestral allele on *S*_2_. A derived‐ancestral incompatibility between sister taxa is only possible in a model that allows incompatible alleles to arise before divergence.

### GENE‐TREE/SPECIES‐TREE DISCORDANCE IS MORE LIKELY AT LOCI ISOLATING SISTER TAXA

**Figure 5:**
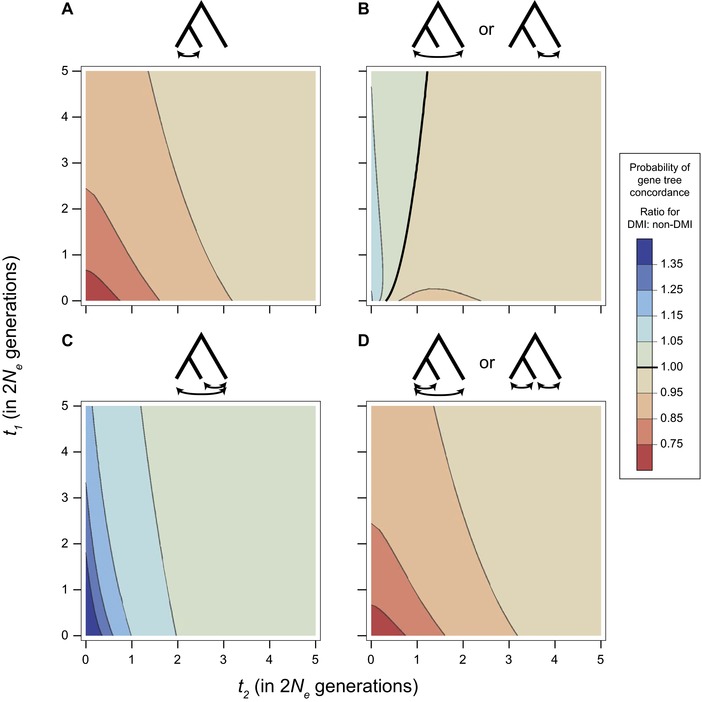
Relative probability of concordance conditioned on the pattern of reproductive isolation. Contour plots show a ratio of the probability of gene–tree species–tree concordance for a DMI locus relative to a random non‐DMI locus. Bold line in (B) and in the legend indicates where the probability of concordance is equal between the two types of loci. All other panels show results that are always either above or below a ratio of 1.

The probability of concordance between the species tree and gene trees at DMI loci depends on the pattern of reproductive isolation considered. Figure 5 shows the probability of concordance for a DMI locus participating in each of the four possible patterns of isolation in a three‐species complex. The values depicted represent a ratio of the probability for gene‐tree/species‐tree concordance at a DMI locus relative to the expected probability of concordance at a random, non‐DMI locus: $1-\frac{2}{3}e^{-t_{2}}$ (Hudson 1983). The greatest deviations from this background probability of gene‐tree/species‐tree concordance occur when little time has elapsed since, and between, speciation events; this is true for all four patterns of isolation (Fig. 5). When *t*_1_ and *t*_2_ are short, branch segments on which incompatible alleles can arise vary greatly between the four types of gene trees (Fig. 1); this in turn leads to larger disparities in gene‐tree/species‐tree discordance among the patterns of isolation (Fig. 5).

Two contrasting patterns emerge as time because divergence grows, depending on whether the locus participates in an incompatibility between sister taxa, *S*_1_ × *S*_2_. For loci participating in an incompatibility isolating sister taxa, the relative probability of gene‐tree/species‐tree concordance is at a minimum when *t*_1_ and *t*_2_ are short, and are 33% less likely to be concordant than a random, non‐DMI locus as these times approach zero (Figs. 5A and D, and S2a). Loci participating in an incompatibility that does not isolate sister taxa are *more* likely to have gene trees that are concordant with the species tree when times are short, up to 67% more likely to be concordant than a random, non‐DMI locus as times approach zero (Figs. 5B and C, and S2b).

The contrast between isolation patterns derives from the restrictions placed on the position of mutations when conditioning on each pattern of reproductive isolation. On concordant trees, alleles involved in sister‐taxa incompatibility must arise before (looking backward in time) the coalescence of lineages from the sister species. This coalescence is, on average, deeper on discordant trees, providing more time for the appropriate mutations to arise. Conversely, the deeper coalescence on discordant trees also reduces the shared branch length leading to sister species. The reduced potential for an incompatible allele shared between the sister species on discordant trees increases the chances that a shared incompatibility isolating both *S*_1_ × *S*_3_ and *S*_2_ × *S*_3_ is due to loci with concordant trees.

### DMI LOCI ARE ON AVERAGE SLIGHTLY MORE LIKELY TO BE DISCORDANT WITH THE SPECIES TREE

The results above were presented separately for the four different patterns of reproductive isolation among three species. To present the probability of gene‐tree/species‐tree discordance across all patterns of isolation, we must take into account the likelihood of each isolation pattern under different histories. For example, pairs of species that have been diverged longer are more likely to harbor incompatibilities, and thus, more likely to be among the species that are isolated. The likelihood that a DMI locus confers a specific pattern of isolation therefore depends on both tip length, *t*_1_, and internal branch length, *t*_2_. As a result, the general, unconditioned probability of gene‐tree/species‐tree concordance at a DMI locus depends on the likelihood of each isolation pattern.

We calculate the relative probability of each isolation pattern by conditioning on the observation of a DMI. The probability of a particular pattern of isolation, *I*_0_, can be written as

(5) PI0| DMI  observed =PI0∑kP(Ik),

where *k* is an index for the patterns of isolation and *P*(*I_k_*) is the denominator in equation (1) from the law of total probability,

(6) $$P\;\left(I_{k}\right)=\sum_{n,m}P\left(I_{k}|\;T_{n},T_{m}\right)P\;\left(T_{n}\right)P\;\left(T_{m}\right)\;.$$

From this, we compare the relative probability of each isolation pattern in our model, which considers ILS, to a model on a fixed species tree with no ILS. For the model with a fixed species tree, we use the expected number of incompatibilities from Wang et al. (2013) to compute the probability of each isolation pattern. In both models, isolation patterns that include an incompatibility between sister species are most likely when tip lengths, *t*_1_, are long relative to the interspeciation time, *t*_2_ (Figs. S3a and d, and S4a and d). The opposite case, with short tip lengths relative to interspeciation time, favors the isolation pattern where both sister species are incompatible with the third species (Figs. S3c and S4c). For intermediate values of *t*_1_ and *t*_2_, the most likely case is an incompatibility that isolates one of the two more distantly related species pairs, that is, isolating *S*_1_ × *S*_3_ or *S*_2_ × *S*_3_ (Figs. S3b and S4b).

The introduction of ILS substantially increases the proportion of incompatibilities that isolate more than one species pair (i.e., isolation patterns in Fig. 3C and D). On a fixed species tree with no ILS, no more than one‐third of incompatibilities ever isolate multiple species pairs, but in a model with ILS, more than half isolate multiple species pairs when *t*_2_ is short relative to *t*_1_ (Fig. S4). This difference arises from the additional branch length that is specific to one lineage on discordant topologies. Coalescence on discordant topologies can only occur in the ancestral population of all three species, substantially increasing lineage‐specific branch lengths. When mutations that produce incompatible alleles are inherited by the same lineage, a derived‐ancestral incompatibility forms with other lineages (Fig. S1). Discordant topologies increase the chances for these shared incompatibilities, especially when *t*_2_ is short relative to *t*_1_.

Putting together the probability of each isolation pattern with its probability of concordance, the general, unconditioned probability of gene‐tree/species‐tree concordance can be calculated by the sum,

(7) P( concordance | DMI  observed )=∑kP concordance |Ik×P(Ik| DMI  observed ).

**Figure 6:**
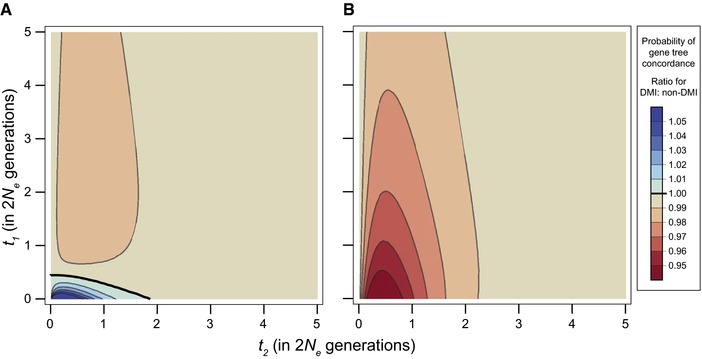
Relative probability of concordance for a DMI locus. (A) Probability of gene–tree/species–tree concordance for a DMI locus in a model restricting incompatibilities from arising in the same population. Concordance is slightly more likely when times are short. Bolded line shows the contour where concordance is equal to the canonical expectation from coalescent theory. (B) Probability of concordance for a DMI locus in a model allowing incompatibilities to arise in the same population. Concordance is always less likely.

Figure 6A shows the general probability of concordance between the species tree and gene trees from a DMI locus, across all patterns of isolation. When tip lengths, *t*_1_, are short, gene‐tree/species‐tree concordance is slightly more likely for DMI loci, up to 27% as both times approach 0. However, for most combinations of times, *t*_1_ and *t*_2_, DMI loci are slightly less likely to be concordant than a random, non‐DMI loci. Overall, gene trees for a locus participating in a DMI have a probability of concordance very slightly below the background value of $1-\frac{2}{3}e^{-t_{2}}$.

### ALLOWING INCOMPATIBILITIES TO ARISE IN THE SAME POPULATION

Our model of DMI loci featuring discordant gene trees has, thus far, explicitly prevented incompatibilities from arising in the same population. This prohibition assumes that selection against incompatibilities is strong enough to prevent the persistence of incompatible alleles in a population. Evidence for the variability of reproductive isolation within populations suggests that the strength of selection may be insufficient to prevent polymorphic incompatibilities from existing (e.g., Corbett‐Detig et al. 2013). To address this possibility in our model, we relax this prohibition, allowing incompatible alleles to arise and segregate in ancestral populations as long as extant lineages do not individually carry the incompatible genotype.

Because our model considers the genealogical history of a DMI locus, incompatibilities that arise in the same population can be incorporated with relative ease. The restriction against these incompatibilities has been enforced by requiring at least one derived allele in an incompatibility to arise after divergence between species pairs. This restriction can be lifted by allowing derived alleles to arise up to (backward in time) the point of coalescence. For derived–ancestral incompatibilities, we continue to enforce the restrictions from mutation order. That is, a derived allele participating in a derived‐ancestral incompatibility may only arise after a mutation has already produced the compatible allele (see Supporting Information Methods). The probability of concordance when considering incompatibilities that arise within populations before divergence can then be calculated by using a relaxed indicator function in equation (2). (The indicator function of each gene tree pair is available in Appendix 2 in Supporting Information Materials).

Overall, allowing the unrestricted emergence of incompatibilities within populations reduces the probability that DMI loci will have gene trees that are concordant with the species tree (Fig. 5B). When tip lengths, *t*_1_, are short, the probability of concordance can be reduced by up to 7% relative to the background expectation. In contrast to the model that restricts DMIs from emerging before divergence, concordance is always less likely than the expectation for non‐DMI loci.

A model that allows incompatibilities to arise within populations also increases the probability of DMIs that isolate sister species (Fig. S5a and d). This results from the additional opportunities for isolating mutations on inner branch segments. Unlike incompatibilities isolating more distantly related species pairs, incompatible alleles on inner branch segments were consistently restricted among sister species from producing incompatibilities within populations. This model also causes the pattern of reproductive isolation to have an even greater impact on the probability of gene‐tree/species‐tree concordance. Qualitatively, the patterns of concordance conditioned on each isolation pattern are similar (see Fig. S6). However, loci participating in incompatibilities between sister taxa, *S*_1_ x *S*_2_, are much less likely to have gene trees that are concordant with the species tree, up to 66% less likely than non‐DMI loci as *t*_1_ and *t*_2_ approach 0. Meanwhile, a locus participating in an incompatibility shared between species pairs *S*_1_ × *S*_3_ and *S*_2_ × *S*_3_ is up to 133% more likely than a non‐DMI locus to have a gene tree that is concordant. As before, the differences in branch length and topology between the types of gene trees are greatest when *t*_1_ and *t*_2_ are short, but these differences are amplified when incompatibilities can arise in the same population before divergence.

### INCOMPATIBLE ALLELES ARE LIKELY TO HAVE ARISEN IN ANCESTRAL POPULATIONS

In the previous section, we allowed pairs of incompatible alleles to arise in the same population before divergence. Because of selection against incompatibilities within populations, such a history for DMI loci should be less common. However, an incompatible allele in a DMI pair could have arisen in an ancestral population without ever having caused an incompatibility within populations. Such an allele would have arisen in the ancestral population and then fixed in one lineage before becoming incompatible with a new mutation after divergence (e.g., the pair of DMI loci in Fig. 4D). As taxa spend more time diverged, incompatibilities between them become more likely to be the result of interactions between new mutations that arise postdivergence. In contrast to this scenario, many formulations of the DM model only allow incompatibilities to form from new mutations after divergence (Orr 1995; Orr and Turelli 2001; Fierst and Hansen 2010; Livingstone et al. 2012; Wang et al. 2013; Fraisse et al. 2014).

**Figure 7:**
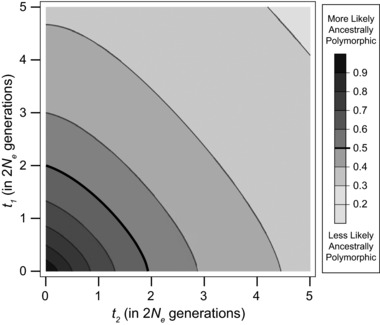
Probability that an incompatibility involves an allele that arose prior to speciation. Bold line shows the contour where a pairwise DMI is equally likely to form from at least one ancestrally arising mutation as from alleles appearing completely after divergence.

To examine the extent to which an incompatible allele is likely to have arisen prior to speciation, we consider its probability in our DM model with ILS. Mutations that occur before divergence give rise to incompatible alleles (but not incompatibilities) in ancestral populations. Branch segments on gene trees positioned before divergence bear such mutations. We can calculate the probability that an incompatibility involves ancestrally arising alleles by adjusting equation (2) to count only mutations on predivergence branch segments,

(8) P(I ANC |Tx,Ty)=p∑α,βP mutation  on xαP( mutation  on yβ)×1(xα,yβ)1ANCxα,yβ,

where **1_ANC_**(*x*_α_, *y*_β_) is 1 when either branch segment is positioned before divergence and 0 otherwise (see Supporting Information Methods). The unconditional probability that an incompatibility involves one ancestrally arising allele can then be calculated following equation (5). Figures 7 and S7 show the proportion of incompatibilities in a three‐species complex involving one incompatible allele that arose in an ancestral population. As branch lengths increase, incompatibilities are more likely to form solely from alleles that arose after divergence.

The pattern of isolation at a DMI has a substantial influence on whether ancestrally arising alleles participate in the incompatibility (Fig. S8). When considering incompatibilities between sister taxa, *S*_1_ × *S*_2_, the proportion of incompatibilities that have at least one ancestrally arising allele depends only on tip length, *t*_1_, and effective population size, *N*_e_. This is because the branch length on which incompatible alleles can arise in the ancestral population depends only on the expected time to coalescence of two lineages from the point of their divergence, which equals 2*N*_e_. The probability of an ancestral allele in a DMI for this case is proportional to the product of the two predivergence segments unique to each lineage and the postdivergence segment of its counterpart (see Fig. S9); this is equal to 2 ⋅ 2*N*_e_ ⋅ *t*_1_. Meanwhile, the probability that a DMI involves only mutations arising postspeciation is proportional to the product of the postdivergence lengths, *t*_1_^2^. For other patterns of isolation, the probability that ancestral alleles participate in the DMI decreases with interspeciation time, *t*_2_.

Overall, for examined speciation times, incompatibilities are likely to involve at least one ancestrally arising allele. When incompatibility loci are allowed to arise in the same population, the probability that incompatibilities result from ancestrally arising alleles increases, but the qualitative patterns are not substantially different from those described above (Fig. S10).

## Discussion

Our results show that DMI loci are slightly more likely to have gene trees that are discordant with the species tree because discordant trees offer more opportunities for the formation of incompatible alleles. This finding follows inevitably from the fact that the mutational target size for a DMI at a particular locus depends on its gene tree, coupled with the constraint that incompatible alleles in a pairwise DMI can only arise on certain pairs of branch segments. A DMI locus is more likely to have a discordant genealogy if these branch segments are longer on discordant trees, and vice versa. The relevant branch segments differ for different patterns of isolation, such that alleles isolating sister taxa are more likely to form on discordant trees, whereas alleles isolating both sister taxa from a third taxon are more likely to form on concordant trees. Our results are in opposition to previous verbal models and suggest that gene tree concordance is unlikely to be useful for identifying DMI loci across the genome. Given the slight excess of discordance expected at DMI loci, gene tree discordance is also unlikely to be useful for this task.

The results presented here assume a neutral model with only ILS acting, leading to several important limitations. Among these are two important considerations that have sometimes been used to argue in favor of greater concordance at speciation loci: postdivergence gene flow and positive selection on incompatibility alleles. The effects of these two phenomena on concordance at DMI loci depend critically on the particulars of the scenario (e.g., the species involved in postdivergence gene flow, the timing of selection, and the species isolated by the incompatibility) and therefore require some discussion. Overall, we argue that the probability of concordance at incompatibility loci is not consistently increased by gene flow or selection during the process of speciation.

At loci conferring reproductive isolation, gene flow can be reduced by the lower fitness of hybrids that inherit the incompatible genotype. This implies that while substantial gene flow between two species may complicate the genealogical history for most of the genome, DMI loci should retain their original history. There is no guarantee that the original history of a DMI locus is concordant with the species tree (i.e., ILS can still occur at this locus), and they therefore may not be any more likely to be concordant than other nonintrogressed loci. Nevertheless, compared to introgressed loci, the relative probability of concordance at DMI loci can be either increased, decreased, or unaffected by postdivergence gene flow.

To see why all of these outcomes are possible, consider two key details concerning patterns of gene flow. First, gene flow must have occurred between the same species isolated by the DMI to have any effect on gene‐tree/species‐tree concordance. General patterns of gene flow between taxa that do not express the incompatibility will not affect the relative probability of concordance. Second, the particular pair of lineages exchanging genes will determine whether DMI loci will be more or less concordant than non‐DMI loci. When gene flow occurs between nonsister taxa (e.g., taxa *S*_1_ and *S*_3_ in Fig. 1), loci with introgressed histories will be more likely to have discordant topologies. Therefore, DMI loci will be relatively more likely to be concordant, though the direction of introgression can have a large effect on the magnitude of this increase (cf. Hibbins and Hahn 2018). Alternatively, when gene flow occurs between sister taxa (taxa *S*_1_ and *S*_2_ in Fig. 1), loci with introgressed histories will actually be more likely to have *concordant* topologies. This occurs because gene flow effectively lengthens the internal branch along which introgressed loci can coalesce, increasing their chances for lineage sorting. The original history, retained by DMI loci, becomes less likely to be concordant with the species tree relative to the history of introgressed loci. Finally, note that theory suggests DMI loci that do not provide a selective advantage in the lineage on which they arose are unlikely to persist in the presence of gene flow (Gavrilets 1997; Bank et al. 2012). In other words, not all DMI loci will be resistant to introgression.

Loci involved in DMIs often bear the signature of positive selection (Coyne and Orr 2004; Orr et al. 2006). Although positive selection on a DMI locus can only increase the probability that its gene tree is concordant with the species tree—because selection reduces *N*_e_, and consequently, the time to coalescence (Kaplan et al. 1989)—the scenarios in which this can occur are limited. To increase the relative probability of concordance, selection on an incompatibility locus must occur in the ancestral population of the sister lineages (*S*_1_ and *S*_2_ in Fig. 1). Lineage‐specific selection, acting on a DMI locus in only one species, can have no effect on the process of lineage sorting that determines concordance, nor can selection in the common ancestor of all three species. When selection on a DMI locus does occur between speciation events, its effects on the relative probability of gene‐tree/species‐tree concordance are greatest when selection is strong and divergence times are short. Figure S11 shows the effects of selection on the probability of concordance compared to a random unselected locus, demonstrating a larger effect with stronger selection. Note also that scenarios involving selection on DMIs that arise in the ancestral population of species *S*_1_ and *S*_2_ can only lead to specific patterns of isolation (in this case, the patterns in Fig. 3B or C). Positive selection in the ancestor of two sister taxa is not expected for DMI loci that isolate them from one another (the patterns in Fig. 3A and D).

Ultimately, the magnitude by which DMI loci are more likely to be concordant depends on how often they are targets of positive selection relative to non‐DMI loci. The results shown in Figure S11 assume that non‐DMIs are under no selection, exaggerating the effects of positive selection on concordance. In fact, a higher chance of concordance from positive selection is not unique to loci that confer reproductive isolation; any locus that has experienced linked selection in the ancestral population is more likely to be concordant with the species tree (Slatkin and Pollack 2006; Stukenbrock et al. 2011; Dutheil et al. 2015). An examination of loci likely to be affected by linked selection is a more direct way of gaining insight into species relationships, regardless of whether they are DMI loci (e.g., Scally et al. 2012; Pease and Hahn 2013; Munch et al. 2016).

As in the traditional DM model, we assumed that incompatibilities have an equal probability of forming between untested allelic combinations, which arise at independent, unlinked loci. An important simplification for our model is the consideration of only a single history for an incompatibility locus in each lineage; that is, we consider only a haploid history for incompatibility loci. Among diploids (and systems with higher ploidy), this simplification is equivalent to assuming that incompatible alleles have fixed in their extant lineages without having passed through the incompatible genotype. Although incompatibility loci can be polymorphic in both extant and ancestral populations (Cutter 2012), tracking this polymorphism in extant populations would require the consideration of multiple lineages in each species. This would necessitate a model of dominance and fitness for each genotypic combination of incompatibility loci. Such a model is outside the scope of our focus here on ILS and the stochastic accumulation of incompatible alleles, but may be interesting for future work. Similarly, we restricted our calculations to three species to produce a tractable model—the number of possible genealogies and isolation patterns grows more than exponentially with the number of species. Although we expect that the main results would remain the same when extended to more species, unique patterns of isolation among multiple species could harbor novel phylogenetic signals.

In examining the possible histories for incompatible alleles, our results highlight how likely they are to have arisen prior to speciation (i.e., in ancestral populations) when they do not affect fitness in conspecific backgrounds. Our results suggest that pairwise DMIs are likely to involve at least one incompatible allele that arose ancestrally until three to four *N*_e_ generations after species divergence. That incompatible alleles are more likely to be from postspeciation mutations as species diverge may not be surprising from a population genetics perspective, but this result may help to clarify an argument on the relative importance of derived versus ancestral alleles in incompatibilities (Cutter 2012). Derived alleles are expected to play a larger role in the formation of incompatibilities because each pairwise incompatibility must involve at least one derived allele (Orr 1995). The distinction between derived and ancestral alleles should, however, not be confused with the genealogical history of participating loci. Mutations that yield incompatible derived alleles can arise both before and after populations diverge; that is, “ancestral” alleles are not derived alleles that arose in ancestral populations. Similarly, a derived–ancestral incompatibility can be the product of two mutations along the same lineage after divergence. Whether an incompatible allele arose in the ancestral population of two or more species depends on the timing of the mutation rather than its state relative to a common ancestor. Thus, the percentage of incompatibilities that involve alleles that arose in ancestral populations decreases with time, but the percentage of ancestral alleles participating in incompatibilities (from derived–ancestral interactions) remains the same. Because the number of potential interactions remains the same, ILS does not change the prediction that incompatibilities should accumulate faster than linearly with divergence time (Orr 1995, Orr and Turelli 2001). This prediction of “snowballing” incompatibilities was made with respect to any two species embedded in a larger tree, a comparison that is not affected by ILS.

The results presented here should be applicable to a number of empirical systems that have been the focus of speciation research. All that is required are short internal branches on a species tree and the ability to carry out crosses between multiple pairs of species. Some examples include species of wild tomato in the genus *Solanum* (Moyle and Nakazato 2010; Pease et al. 2016) and subspecies of mouse within *Mus musculus* (White et al. 2009; Wang et al. 2015). One of the most interesting systems to which these predictions can be applied are the three species in the *Drosophila simulans* clade, which were the focus of one of the seminal studies purporting to demonstrate the unique phylogenetic signal at speciation loci (Ting et al. 2000). However, this study produced what is now thought to be a discordant gene tree from a DMI locus. The tree of the *D. simulans* clade constructed from the hybrid incompatibility locus *OdsH* in Ting et al. (2000) places *D. simulans* as most closely related to *D. mauritiana*. More recently, whole‐genome sequence data (Garrigan et al. 2012) found the best‐fitting maximum‐likelihood tree to be one that groups *D. simulans* with *D. sechellia* to the exclusion of *D. mauritiana*. This inference is supported by an excess of gene trees concordant with this topology in regions of low recombination, where ILS should have the least effect (Pease and Hahn 2013).

The identification of loci participating in hybrid incompatibilities between multiple closely related species pairs has spurred many new analyses, including phylogenetic comparisons among species (Cattani and Presgraves 2009; Scarpino et al. 2013; Sherman et al. 2014; Wang et al. 2015). These comparative approaches can help to elucidate the timing and progression of reproductive isolation (Moyle and Payseur 2009). But the analysis of incompatibilities between multiple species pairs also introduces genealogical ambiguity at incompatibility loci. When incompatible alleles in a DMI arise on gene trees with different topologies, identifying which branch of the species tree they arose on becomes challenging. In fact, incompatible alleles arising on discordant trees may be mapped onto the wrong branches of the species tree by standard methods. Although a derived allele in a pairwise DMI will always be inherited by at least one of the lineages isolated by the incompatibility, the other allele can originate on branches that are shared with uninvolved lineages. For example, in the absence of ILS an incompatibility isolating only the sister species among three taxa (Fig. 3A) would be interpreted as the result of two mutations, each uniquely inherited by one of the sister species. However, in the presence of ILS such an incompatibility could involve a mutation inherited by one of the sister species and a third taxon (as in Fig. 4C and D). Discordance between gene trees is most pronounced when little time separates speciation events, and this is often the case for model systems of speciation, where the ability to perform interspecific crosses is a useful tool for genetic investigation (e.g., True et al. 1996; Slotman et al. 2004; Sweigart et al. 2006; Moyle and Nakazato 2008; Matute and Coyne 2010; White et al. 2011). Under these circumstances, inferences about the origin of incompatibilities from comparative mapping need to be mindful of the genealogical history at DMI loci.

## Methods

### CALCULATING BRANCH LENGTHS

To calculate the probability of gene‐tree/species‐tree concordance at a DMI locus, we find the probability of concordance conditioned on a particular pattern of isolation, *I*. This requires calculating the branch lengths from each pair of gene trees, and the indicator function representing the opportunity to produce an incompatibility (eq. 2). The indicator function can be represented as a matrix, **1** *_Tx_*,*_Ty_*, with rows and columns corresponding to the branches of the respective gene trees *T_x_* and *T_y_*. With four types of gene trees, there are 10 unique matrices (see Appendix 1 in Supporting Information Materials).

**Figure 8:**
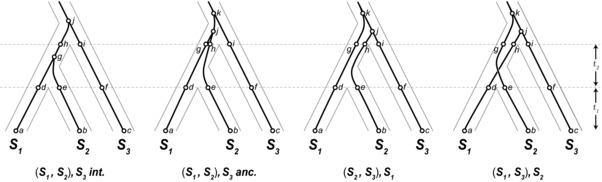
Four types of gene trees with branch segments labeled. Concordant trees are divided into those that coalesce in the time between species divergences (*int*er‐) and those that coalescence in the *anc*estral population.

Branch segments on each of the four types of gene trees are labeled in Figure 8. The probability of a mutation on the branch segments of each tree, *T*, can be assembled into the vector *B_T_*. We order the lengths of each segment (using the segment names as labels for their expected lengths for convenience, that is, *ij* = *L*(*i* − *j*)) in the vectors as follows:

(9) $$\begin{array}{ccc} B_{\left(S1,S2\right)S3\;int.} & = & \left[ad,dg,be,eg,cf,fi,ij,gh,hj\right],\\ \;B_{\left(S1,S2\right)S3\;anc.} & = & \left[ad,dg,gj,be,eh,hj,cf,fi,ik,jk\right],\\ \;B_{\left(S2,S3\right)S1} & = & \;\left[ad,dg,gk,be,eh,hj,cf,fi,ij,jk\right],\\ \;B_{\left(S1,S3\right)S2} & = & \;\left[ad,dh,hj,be,eg,gk,cf,fi,ij,jk\right]. \end{array}$$

The length of segments whose ends are not coalescent events are simply *t*_1_ or *t*_2_. Segments that coalesce in the ancestral population of all three species have expected lengths (in 2*N*_e_ coalescent units) of 1/3, 1, and 4/3, corresponding to the time to coalescence from three‐to‐two, two‐to‐one, and three‐to‐one lineages, respectively.

Several segments on the (*S*_1_, *S*_2_) *S*_3_ *int*. tree have expected values that are conditioned on the first coalescence occurring by *t*_2_. This condition distinguishes this type of tree from the ancestrally coalescing tree with the same topology. Let *v* be a random variable from 0 to *t*_2_, representing the time from the most recent population divergence to the first coalescence event in the (*S*_1_, *S*_2_) *S*_3_ *int*. tree. The probability distribution function of *v* is given by the exponential distribution function for the coalescence of two lineages, *e^−t^*, divided by the probability of coalescence by *t*_2_ (see Mendes and Hahn 2017). Thus,

(10) $$f_{v}\;\left(t\right)=\frac{e^{-t}}{1-e^{-t_{2}}}\;;\;\;0\leq t\leq t_{2}.$$

Let *q*(*t*_2_) be the expected value of *v* for a given value of *t*_2_, then the expected time from the first population divergence to the first coalescence in the (*S*_1_, *S*_2_) *S*_3_ *int*. tree is

(11) $$q\;\left(t_{2}\right)=\int_{0}^{t_{2}}\frac{te^{-t}}{1-e^{-t_{2}}}dt\;=\;1-\frac{t_{2}}{e^{t_{2}}-1}.$$

Filling in the segment lengths from equation (9), the values for *B*_T_ become

(12) $$\begin{array}{ccc} B_{\left(S1,S2\right)S3\;int.} & = & \left[t_{1},q\left(t_{2}\right),t_{1},q\left(t_{2}\right),t_{1},t_{2},1,t_{2}-q\left(t_{2}\right),1\right],\\ \;B_{\left(S1,S2\right)S3\;anc.} & = & \left[t_{1},t_{2},\frac{1}{3},t_{1},t_{2},\frac{1}{3},t_{1},t_{2},\frac{4}{3},1\right],\\ \;B_{\left(S2,S3\right)S1} & = & \left[t_{1},t_{2},\frac{4}{3},t_{1},t_{2},\frac{1}{3},t_{1},t_{2},\frac{1}{3},1\right],\\ \;B_{\left(S1,S3\right)S2} & = & \;\left[t_{1},t_{2},\frac{1}{3},t_{1},t_{2},\frac{4}{3},t_{1},t_{2},\frac{1}{3},1\right]. \end{array}$$

With the terms in equation (2) written as vectors and matrices, *P*(*I|T_x_, T_y_*) can be written conveniently as the matrix product:

(13) $$P\;\left(I|T_{x},T_{y}\right)=\;pB_{T_{x}}1_{T_{x},T_{y}}B_{T_{y}}.$$

For four gene trees, there are 16 combinations of *T_x_*, *T_y_*. We form a 4 × 4 matrix, ***D***(*I*), for each isolation pattern *I*, whose entries are the above matrix product (eq. 13) for each gene tree pair. The rows and columns in ***D***(*I*) are ordered so that they are consistent with the order of trees in Figure 8.

To arrive at the probability of each gene tree pair, Bayes’ theorem is applied (eq. 1). The numerator from equation (1) can be calculated from each element of the ***D***(*I*) product matrix, multiplied by the unconditional probability of each gene tree pair. The probability for each type of gene tree (Fig. 1) can be assembled into a vector of tree probabilities, *T*_prob_, as

(14) T prob =1−e−t2,13e−t2,13e−t2,13e−t2.

The numerator in equation (1) for each gene tree pair can then be represented as the elementwise product of ***D***(*I*) with the outer product of *T*_prob_ with itself. We call this 4 × 4 matrix ***P***(*I*),

(15) PI=T prob ⊗T prob ∘DI.

The denominator for equation (1) is the sum of all elements in the ***P***(*I*) matrix.

Returning to equation (4), the probability of concordance for a single DMI locus is given by the sum of all elements in the first two rows and columns of ***P***(*I*), divided by twice the sum of all elements. Note that the parameters *p* and 2*N*_e_ *μ* do not appear in the probability of concordance as they are cancelled by the denominator in equation (1).

### MUTATION ORDER AND DERIVED–ANCESTRAL INCOMPATIBILITIES

**Figure 9:**
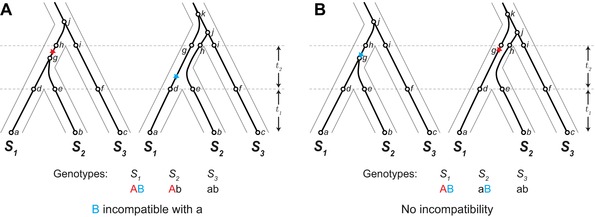
Mutation order determines the potential for a derived‐ancestral incompatibility. Red and blue stars mark the position of the first and second mutations producing incompatible alleles. The ancestral genotype for the two loci is denoted “ab”, with the mutations producing derived alleles “A” and “B”. (A) Derived–ancestral incompatibility from the derived allele in the *S*_1_ lineage with the ancestral allele in the *S*_3_ lineage. (B) No incompatibility forms because the derived–ancestral genotype persists in the *S*_2_ lineage.

Thus far, the probability of each mutation in a DMI pair has been treated as an independent event. That is, the joint probability is calculated as a product of the mutation probabilities on each branch (eq. 2). Although this assumption is valid for derived–derived incompatibilities, greater care must be taken for derived–ancestral incompatibilities due to the order in which mutations must occur. Consider a potential derived–ancestral incompatibility between a mutation on segment *g–h* of the (*S*_1_, *S*_2_) *S*_3_ *int*. tree and a mutation on segment *d–g* of the (*S*_2_, *S*_3_) *S*_1_ tree, as in Figure 9.

A mutation on segment *g–h*, followed by a mutation on *d–g* can result in a derived–ancestral incompatibility between *S*_1_ and *S*_3_. The converse, a mutation that occurs first on segment *d–g* followed by a mutation on *g–h*, cannot result in a derived–ancestral incompatibility because the ancestral allele persists in the *S*_2_ lineage. As the incompatible allelic combination already exists in *S*_2_, this combination cannot be the cause of an incompatibility between *S*_1_ and *S*_3_.

This asymmetry from mutation order occurs because the derived allele in a derived–ancestral incompatibility must arise from the second mutation. If the derived allele arose from the first mutation, it would immediately produce the incompatible genotype. In the previous example, when the second mutation arises on segment *d–g*, the derived allele is inherited by the *S*_1_ lineage, whereas when the second mutation arises on segment *g–h*, both the *S*_1_ and *S*_2_ lineage inherit the derived allele. Between branch segments that have contemporaneous endpoints, this asymmetry does not exist and the order of mutations does not matter. For example, the first mutation on segment *a–d* of either tree in Figure 9 leads to an ancestral allele that can be incompatible with a second mutation at segment *a–d* on the other tree.

Differences in mutation order produce this asymmetry only when the inheritance of the derived allele is made ambiguous by the timing of a coalescent event. This is only possible when the mutations are from segments that overlap temporally. Most pairs of segments where mutation order is ambiguous occur before any population divergence has occurred (Fig. 8). When incompatibilities are restricted to arising only after populations diverge, these segments do not cause any incompatibilities. The only gene tree pairs where mutation order must be considered in this model are those that involve the (*S*_1_, *S*_2_) *S*_3_ *int*. tree. Here, the coalescence in the ancestral population of *S*_1_ and *S*_2_ can change the identity of the derived allele in an *S*_1_ × *S*_3_ incompatibility and an *S*_2_ × *S*_3_ incompatibility.

Returning to our earlier example, we calculate the joint probability of two mutations leading to a derived–ancestral incompatibility from segments *g–h* on an (*S*_1_, *S*_2_) *S*_3_ *int*. tree and *d–g* on an (*S*_2_, *S*_3_) *S*_1_ tree. Such an incompatibility requires the first mutation to be on segment *g–h*, restricting the timing of the second mutation on *d–g*. The probability of the first mutation is unrestricted and is equal to the branch length of *g–h*, which has an expected value of *t*_2_ – *q*(*t*_2_). Let *τ* be the time from the *S*_1_, *S*_2_ divergence to the first mutation on segment *g–h*. Assuming that a single mutation occurs on segment *g–h*, the probability of the first mutation should be uniformly distributed along the length of segment *g–h*. Then,

(16) $$\begin{array}{ccc} E[\tau ] & = & q\left(t_{2}\right)+\frac{1}{2}\left(t_{2}-q\left(t_{2}\right)\right)\\ & = & \frac{1}{2}\left(t_{2}+q\left(t_{2}\right)\right). \end{array}$$

Mutations that occur before the first mutation on *g–h* do not cause an incompatibility, thus the expected value of *τ* is also the expected length of the subsegment of *d–g* from which a second mutation may arise. The probability of an incompatibility from these two segments can then be calculated from the product of E[*τ*] and *t*_2_ – *q*(*t*_2_).

This probability applies for each of the gene tree pairs involving (*S*_1_, *S*_2_) *S*_3_ *int*. and any other gene tree for producing a derived–ancestral incompatibility between *S*_1_ and *S*_3_. Similarly, the same reasoning can be applied for interactions between mutations on *g–h* and *e–h* for incompatibilities between *S*_2_ and *S*_3_. When both gene trees are (*S*_1_, *S*_2_) *S*_3_ *int*., the calculation is more involved due to the coalescence at the end of branch segments from both trees (see Supporting Information Methods).

## DATA ACCESSIBILITY

All plots in this manuscript are analytical solutions produced with the help of Mathematica software. Files containing these calculations in Mathematica format are provided in Supporting Information Material.

Associate Editor: S. Wright

## Supporting information


**Figure S1**. Derived–ancestral incompatibility shared between *S_1_* × *S_2_* and *S_1_* × *S_3_* due to a shared ancestral allele.

**Figure S2**. Relative probability of concordance for DMI loci with two different patterns of isolation.

**Figure S3**. Relative probability of concordance conditioned on the pattern of reproductive isolation (polymorphic incompatibilities allowed).

**Figure S4**. Probability for different patterns of reproductive isolation; model of DMI with loci on a fixed species tree.

**Figure S5**. Probability for different patterns of reproductive isolation; model of DMI with loci subject to ILS.

**Figure S6**. Probability that an incompatibility involves an allele that arose prior to speciation.

**Figure S7**. Probability for different patterns of reproductive isolation; model of DMI with potentially polymorphic loci in addition to being subject to ILS.

**Figure S8**. Probability that an incompatibility involves an ancestrally arising locus conditioned on different patterns of reproductive isolation.

**Figure S9**. The probability of an ancestrally arising allele in a DMI isolating sister taxa.

**Figure S10**. Probability that an incompatibility involves an ancestrally arising locus; model of DMI with potentially polymorphic loci in addition to being subject to ILS.

**Figure S11**. Effects of selection on the probability of concordance at a DMI locus.

**Figure S12**. Calculating constraints on derived–ancestral incompatibilities due to mutation order on a pair of (*S1*, *S2*) *S3* int. trees.
**Table S1**. Probabilities for incompatibility‐participating mutations that depend on mutation order note that patterns A2 and A5 correspond to calculations from equations (16) (main text) and (S4), respectively.

**Supplementary Material**: Appendix 1. Incompatibility matrices.

**Supplementary Material**: Appendix 2. Incompatibility matrices with incompatibilities allowed to arise in the same population.
