# Supplementary material for: Speciation genes are more likely to have discordant gene trees
Source: Evol Lett. 2018 Aug 8;2(4):281–96. doi: 10.1002/evl3.77 (PMC6121824; doi:10.1002/evl3.77)
Supplement: Supplementary file 12 — Figure S12. Calculating constraints on derived–ancestral incompatibilities due to mutation order on a pair of (S1, S2) S3 int. trees. Table S1. Probabilities for incompatibility‐participating mutations that depend on mutation order note that patterns A2 and A5 correspond to calculations from equations (16) (main text) and (S4), respectively. [file EVL3-2-281-s017.pdf]

Supplemental Methods

Probability of a mutation on a branch segment

Assuming mutations are rare, the probability of a mutation on a particular branch segment, i.e. x_α and y_β, is proportional to the length of that segment (Hudson 1992). The probability of a mutation can be estimated as the probability for at least one mutation on the given segment, with no mutations on the rest of the tree,

$$\begin{aligned} P(\text{mutation on } x_\alpha) &= \left[1 - e^{-2N_e\mu\cdot L(x_\alpha)}\right]\left[e^{-2N_e\mu\sum_\alpha L(x_\alpha)}\right] \\ &= \left[1 - \left(1 - 2N_e\mu\cdot L(x_\alpha) + O(\mu^2)\right)\right]\left[1 - 2N_e\mu\sum_\alpha L(x_\alpha) + O(\mu^2)\right] \\ &\approx 2N_e\mu\cdot L(x_\alpha) \end{aligned} \quad \text{(S1)}$$

where $2N_e\mu$ is the population mutation parameter, $L(x_\alpha)$ is the length of branch segment x_α, and a Taylor series approximation has been made to remove terms of order μ^2 and higher.

Mutation order for (S_1, S_2) S_3 int. *gene trees*

Let τ_1 be a random variable representing the time from the S_1, S_2 divergence to the first mutation on segment *g-h* on the first tree. Let v_1 and v_2 be random variables representing the time from the S_1, S_2 divergence to the most recent coalescence on the first and second tree, respectively. The first mutation on segment *g-h* is unconstrained and its probability remains $t_2 - q(t_2)$. The second mutation on segment *d-g* is constrained by both the timing of the first mutation as well as the timing of the coalescence event at one end of the segment (see Fig. S9). If the mutation on the first tree occurs before the coalescence on the second tree, the second mutation can arise on the entire length of the *d-g* segment, up to time v_2. If the coalescence on the second tree occurs before the first mutation, the second mutation may only arise on the truncated *d-g* segment, up to length τ_1. The probability of an incompatible mutation arising on *d-g* is the expected length,

$$P(I \text{ mutation on } d\text{-}g) = \mathrm{P}(\tau_1 > v_2)\mathrm{E}[v_2|\tau_1 > v_2] + \mathrm{P}(\tau_1 < v_2)\mathrm{E}[\tau_1|\tau_1 < v_2] \quad \text{(S2)}$$

Let f_v be the probability distribution function for v_1 and v_2 (as described earlier in Equation 11; main text), and f_τ be the probability distribution function for τ_1. Because we assume only a single mutation occurs on segment *g-h*, the probability of τ_1 is uniformly distributed along its length,
t_2 - v_1. The probability distribution function, f_τ, is then

$$f_\tau(t) = \int_0^t \frac{1}{t_2 - x} f_v(x)\,dx \quad \text{(S3)}$$

The conditional expectations can thus be expressed as

$$\mathrm{E}[\tau_1|\tau_1 < v_2] = \int_0^{t_2} t f_\tau(t) \left[\int_t^{t_2} \frac{f_v(x)dx}{\mathrm{P}(\tau_1 < v_2)} \right] dt$$
$$\mathrm{E}[v_2|\tau_1 > v_2] = \int_0^{t_2} t f_v(t) \left[\int_t^{t_2} \frac{f_\tau(x)dx}{P(\tau_1 > v_2)} \right] dt$$

When these expectations are substituted into Equation S2, the probability of an incompatible mutation arising on *d-g* becomes,

$$P(I \text{ mutation on } d\text{-}g) = \int_0^{t_2} t f_v(t) \left[\int_t^{t_2} f_\tau(x)dx \right] dt + \int_0^{t_2} t f_\tau(t) \left[\int_t^{t_2} f_v(x)dx \right] dt \qquad \text{(S4)}$$

The probability of an incompatibility between these segments can be calculated from the product of the unconstrained mutation probability on segment *g-h*, $t_2 - q(t_2)$, and Equation S4. Since segments *d-g* and *g-h* exist on both trees, this product contributes twice to the probability of a derived-ancestral incompatibility when both loci have (S_1, S_2) S_3 int. trees.

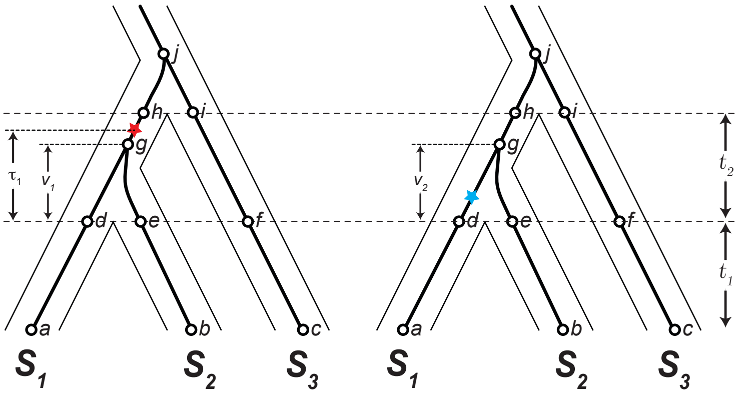

Figure S12. Calculating constraints on derived-ancestral incompatibilities due to mutation order on a pair of (S_1, S_2) S_3 int. trees.
While this figure shows the mutation on the left tree, marked by the red star, as occurring first, this is not always true. Because v_1, v_2, and τ_1 are all random variables, it is possible for the mutation on the second tree, marked by the blue star, to be the first mutation.

Mutation order for incompatibilities that arise ancestrally

As previously noted, there are a number of branch segments with ambiguous endpoints in the ancestral population, forcing us to consider mutation order for derived-ancestral incompatibilities when incompatible alleles are allowed to arise before divergence. These calculations follow Eqs. S2 and S4; the timing of the first mutation restricts the length of the segment on which the second mutation may occur. Let λ_{Ai} be a random variable representing the length of the segment on which an unrestricted second mutation might occur for a pattern of branch segment asymmetry, A_i. Let τ_{Ai} be a random variable representing the time from the oldest divergence to the first mutation for this asymmetry pattern. The probability of the first

mutation is unrestricted and equal to the expected length of the first branch segment. The probability of the second mutation depends on whether the first mutation precedes the coalescence of the second branch segment and can be expressed as

$$\mathrm{P}(\mu_2) = \mathrm{P}(\tau_{Ai} > \lambda_{Ai})\mathrm{E}[\lambda_{Ai}|\tau_{Ai} > \lambda_{Ai}] + \mathrm{P}(\tau_{Ai} < \lambda_{Ai})\mathrm{E}[\tau_{Ai}|\tau_{Ai} < \lambda_{Ai}] \qquad \text{(S5)}$$

Branch segments with mutation order asymmetry affecting the probability of derived-ancestral incompatibilities are labeled with their respective asymmetry patterns in Appendix 2. The probability distribution functions for λ_{Ai} can be calculated from the time to coalescence for three-to-two and two-to-one lineages. Similarly, τ_{Ai} can be calculated from the convolution of these functions and the probability distribution function for a mutation uniformly distributed along the appropriate branch. The probability distribution functions for λ_{Ai} and τ_{Ai}, as well as P(μ_1) and P(μ_2), are listed for each asymmetry pattern in Table S1.

We modify Equation 13 (main text) to calculate the probability of concordance with polymorphic incompatibilities. Let $\mathbf{1R}_{Tx,Ty}$ be the indicator matrix for segments on trees T_x and T_y, whose elements are 1 when the segment combination can lead to an incompatibility and 0 otherwise. Let $\mathbf{A}_{Tx,Ty}$ be a matrix whose elements are the probabilities for a derived-ancestral incompatibility that depends on mutation order (i.e. P(μ_1) · P(μ_2) for that segment combination). The probability of an incompatibility with isolation pattern *I*, conditioned on gene trees T_x and T_y, is then

$$P(I|T_x, T_y) = p\left(B_{T_x}\mathbf{1R}_{T_x,T_y}B_{T_y} - B_{T_x}\mathbf{1}_{\mathbf{A}_{T_x,T_y}}B_{T_y} + \mathbf{A}_{T_x,T_y} \circ \mathbf{1R}_{T_x,T_y}\right) \qquad \text{(S6)}$$

where $\mathbf{1}_{\mathbf{A}_{T_x,T_y}}$ is a matrix whose elements are 1 for non-zero entries of $\mathbf{A}_{Tx,Ty}$ and 0 otherwise.

As before, we can form the 4x4 matrix, ***D***(*I*), for each isolation pattern *I* from Equation S6, and calculate concordance probabilities from Equation 4 (main text).

Table S1. Probabilities for incompatibility-participating mutations that depend on mutation order Note that patterns A_2 and A_5 correspond to calculations from Eqs. 16 (main text) and S4 respectively.

Pattern	$f_{\lambda_{Ai}}(t)$	$f_{\tau_{Ai}}(t)$	$P(\mu_1)$	$P(\mu_2)$
A_1	e^{-t}	$\int_0^t 3e^{-3(t-s)}\left[\int_s^\infty \frac{e^{-x}}{x}dx\right]ds$	1	$1-\frac{3}{4}\log(2)$
A_2	1 for $t=t_2$	$\int_0^t \frac{1}{t_2-x} f_v(x)\,dx$	$t_2-q(t_2)$	$\frac{1}{2}(t_2+q(t_2))$
A_3	$\frac{3}{2}(e^{-t}-e^{-3t})$	$\int_t^\infty \frac{e^{-x}}{x}dx$	1	$\frac{4}{3}-\frac{25}{18}\log(2)$
A_4	$3e^{-3t}$	$\int_t^\infty \frac{e^{-x}}{x}dx$	1	$\frac{1}{3}-\frac{1}{9}\log(4)$
A_5	$f_v(t)$	$\int_0^t \frac{1}{t_2-x} f_v(x)\,dx$	$t_2-q(t_2)$	(see Eq. S4)
A_6	$3e^{-3t}$	$\int_0^t 3e^{-3(t-s)}\left[\int_s^\infty \frac{e^{-x}}{x}dx\right]ds$	1	$\frac{1}{3}-\frac{1}{9}\log(2)$
A_7	$\frac{3}{2}(e^{-t}-e^{-3t})$	$\int_0^t 3e^{-3(t-s)}\left[\int_s^\infty \frac{e^{-x}}{x}dx\right]ds$	1	$\frac{4}{3}-\frac{77}{72}\log(2)$
