# Supplementary material for: Speciation genes are more likely to have discordant gene trees
Source: Evol Lett. 2018 Aug 8;2(4):281–96. doi: 10.1002/evl3.77 (PMC6121824; doi:10.1002/evl3.77)
Supplement: Supplementary file 13 — Supplementary Material: Appendix 1. Incompatibility matrices. [file EVL3-2-281-s012.pdf]

Appendix 1. Incompatibility Matrices

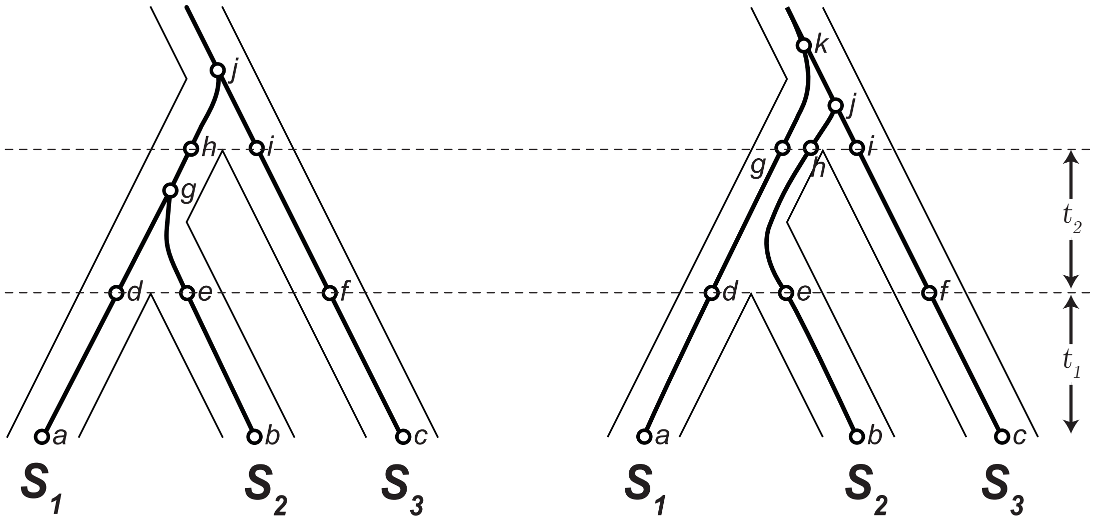

Rows: (S_1, S_2) S_3 int.   Columns: (S_2, S_3) S_1

		S_1 derived			S_2 derived			S_3 derived			S_2, S_3 derived
		ad	dg	gk	be	eh	hj	cf	fi	ij	jk
S_1 derived	ad	S1 x S2<br>S1 x S3	S1 x S2<br>S1 x S3	S1 x S2<br>S1 x S3	S1 x S2	S1 x S2	S1 x S2	S1 x S3	S1 x S3	S1 x S3	S1 x S2<br>S1 x S3
	dg	S1 x S2<br>S1 x S3	–	–	S1 x S2	–	–	S1 x S3	S1 x S3	S1 x S3	–
S_2 derived	be	S1 x S2	S1 x S2	S1 x S2	S1 x S2<br>S2 x S3	S1 x S2<br>S2 x S3	S1 x S2<br>S2 x S3	S2 x S3	S2 x S3	S2 x S3	S1 x S2
	eg	S1 x S2	–	–	S1 x S2<br>S2 x S3	–	–	S2 x S3	S2 x S3	S2 x S3	–
S_3 derived	cf	S1 x S3	S1 x S3	S1 x S3	S2 x S3	S2 x S3	S2 x S3	S1 x S3<br>S2 x S3	S1 x S3<br>S2 x S3	S1 x S3<br>S2 x S3	S1 x S3
	fi	S1 x S3	S1 x S3	S1 x S3	S2 x S3	S2 x S3	S2 x S3	S1 x S3<br>S2 x S3	S1 x S3<br>S2 x S3	S1 x S3<br>S2 x S3	S1 x S3
	ij	S1 x S3	S1 x S3	–	S2 x S3	S2 x S3	–	S1 x S3<br>S2 x S3	S1 x S3<br>S2 x S3	–	–
S_1, S_2 derived	gh	S1 x S3	S1 x S3	-	S2 x S3	S2 x S3	-	S1 x S3<br>S2 x S3	S1 x S3<br>S2 x S3	S1 x S3<br>S2 x S3	–
	hj	S1 x S3	S1 x S3	–	S2 x S3	S2 x S3	–	S1 x S3<br>S2 x S3	S1 x S3<br>S2 x S3	–	–

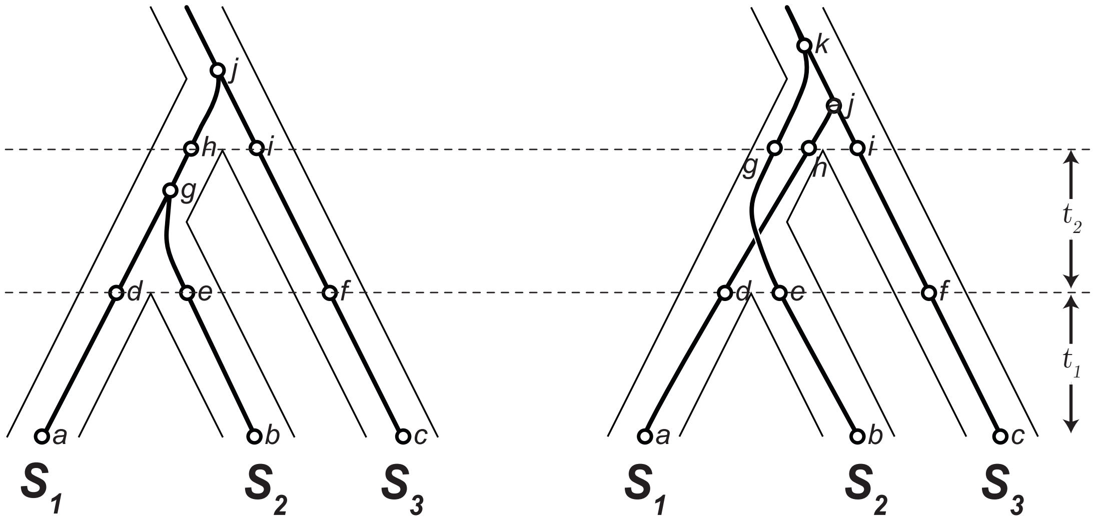

Rows: (S_1, S_2) S_3 int. Columns: (S_1, S_3) S_2

		S_1 derived			S_2 derived			S_3 derived			S_1, S_3 derived
		ad	dh	hj	be	eg	gk	cf	fi	ij	jk
S_1 derived	ad	S1 x S2<br>S1 x S3	S1 x S2<br>S1 x S3	S1 x S2<br>S1 x S3	S1 x S2	S1 x S2	S1 x S2	S1 x S3	S1 x S3	S1 x S3	S1 x S2
	dg	S1 x S2<br>S1 x S3	–	–	S1 x S2	–	–	S1 x S3	S1 x S3	S1 x S3	–
S_2 derived	be	S1 x S2	S1 x S2	S1 x S2	S1 x S2<br>S2 x S3	S1 x S2<br>S2 x S3	S1 x S2<br>S2 x S3	S2 x S3	S2 x S3	S2 x S3	S1 x S2<br>S2 x S3
	eg	S1 x S2	–	–	S1 x S2<br>S2 x S3	–	–	S2 x S3	S2 x S3	S2 x S3	–
S_3 derived	cf	S1 x S3	S1 x S3	S1 x S3	S2 x S3	S2 x S3	S2 x S3	S1 x S3<br>S2 x S3	S1 x S3<br>S2 x S3	S1 x S3<br>S2 x S3	S2 x S3
	fi	S1 x S3	S1 x S3	S1 x S3	S2 x S3	S2 x S3	S2 x S3	S1 x S3<br>S2 x S3	S1 x S3<br>S2 x S3	S1 x S3<br>S2 x S3	S2 x S3
	ij	S1 x S3	S1 x S3	–	S2 x S3	S2 x S3	–	S1 x S3<br>S2 x S3	S1 x S3<br>S2 x S3	–	–
S_1, S_2 derived	gh	S1 x S3	S1 x S3	-	S2 x S3	S2 x S3	-	S1 x S3<br>S2 x S3	S1 x S3<br>S2 x S3	S1 x S3<br>S2 x S3	–
	hj	S1 x S3	S1 x S3	–	S2 x S3	S2 x S3	–	S1 x S3<br>S2 x S3	S1 x S3<br>S2 x S3	–	–

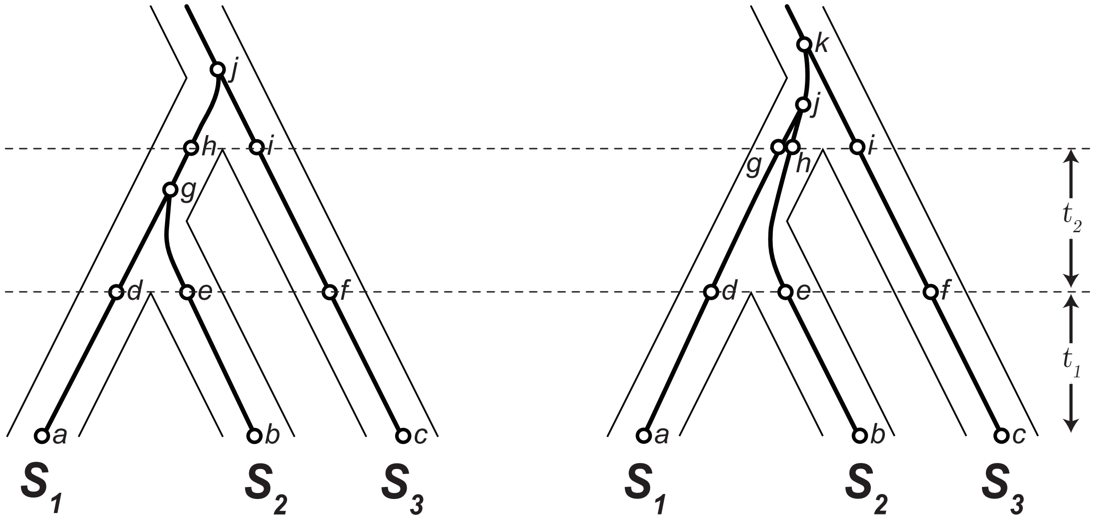

<u>Rows</u>: (S_1, S_2) S_3 int.

<u>Columns</u>: (S_1, S_2) S_3 anc.

		S_1 derived			S_2 derived			S_3 derived			S_1, S_2 derived
		ad	dg	gj	be	eh	hj	cf	fi	ik	jk
S_1 derived	ad	S1 x S2<br>S1 x S3	S1 x S2<br>S1 x S3	S1 x S2<br>S1 x S3	S1 x S2	S1 x S2	S1 x S2	S1 x S3	S1 x S3	S1 x S3	S1 x S3
	dg	S1 x S2<br>S1 x S3	–	–	S1 x S2	–	–	S1 x S3	S1 x S3	S1 x S3	S1 x S3
S_2 derived	be	S1 x S2	S1 x S2	S1 x S2	S1 x S2<br>S2 x S3	S1 x S2<br>S2 x S3	S1 x S2<br>S2 x S3	S2 x S3	S2 x S3	S2 x S3	S2 x S3
	eg	S1 x S2	–	–	S1 x S2<br>S2 x S3	–	–	S2 x S3	S2 x S3	S2 x S3	S2 x S3
S_3 derived	cf	S1 x S3	S1 x S3	S1 x S3	S2 x S3	S2 x S3	S2 x S3	S1 x S3<br>S2 x S3	S1 x S3<br>S2 x S3	S1 x S3<br>S2 x S3	S1 x S3<br>S2 x S3
	fi	S1 x S3	S1 x S3	S1 x S3	S2 x S3	S2 x S3	S2 x S3	S1 x S3<br>S2 x S3	S1 x S3<br>S2 x S3	S1 x S3<br>S2 x S3	S1 x S3<br>S2 x S3
	ij	S1 x S3	S1 x S3	–	S2 x S3	S2 x S3	–	S1 x S3<br>S2 x S3	S1 x S3<br>S2 x S3	–	–
S_1, S_2 derived	gh	S1 x S3	S1 x S3	-	S2 x S3	S2 x S3	-	S1 x S3<br>S2 x S3	S1 x S3<br>S2 x S3	S1 x S3<br>S2 x S3	S1 x S3<br>S2 x S3
	hj	S1 x S3	S1 x S3	–	S2 x S3	S2 x S3	–	S1 x S3<br>S2 x S3	S1 x S3<br>S2 x S3	–	–

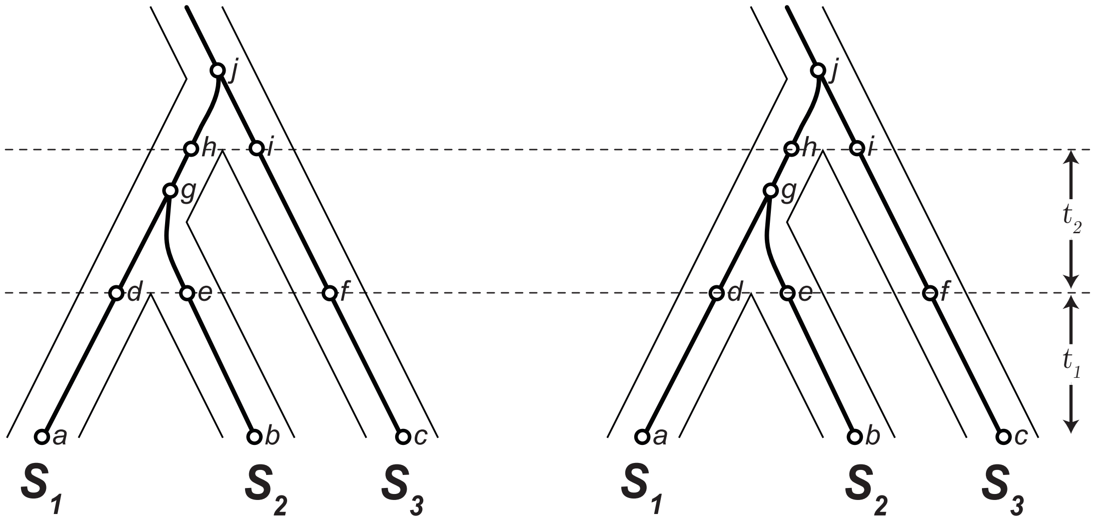

<u>Rows</u>: (S_1, S_2) S_3 int.

<u>Columns</u>: (S_1, S_2) S_3 int.

		S_1 derived		S_2 derived		S_3 derived			S_1, S_2 derived	
		ad	dg	be	eg	cf	fi	ij	gh	hj
S_1 derived	ad	S1 x S2<br>S1 x S3	S1 x S2<br>S1 x S3	S1 x S2	S1 x S2	S1 x S3	S1 x S3	S1 x S3	S1 x S3	S1 x S3
	dg	S1 x S2<br>S1 x S3	–	S1 x S2	–	S1 x S3	S1 x S3	S1 x S3	S1 x S3	S1 x S3
S_2 derived	be	S1 x S2	S1 x S2	S1 x S2<br>S2 x S3	S1 x S2<br>S2 x S3	S2 x S3	S2 x S3	S2 x S3	S2 x S3	S2 x S3
	eg	S1 x S2	–	S1 x S2<br>S2 x S3	–	S2 x S3	S2 x S3	S2 x S3	S2 x S3	S2 x S3
S_3 derived	cf	S1 x S3	S1 x S3	S2 x S3	S2 x S3	S1 x S3<br>S2 x S3	S1 x S3<br>S2 x S3	S1 x S3<br>S2 x S3	S1 x S3<br>S2 x S3	S1 x S3<br>S2 x S3
	fi	S1 x S3	S1 x S3	S2 x S3	S2 x S3	S1 x S3<br>S2 x S3	S1 x S3<br>S2 x S3	S1 x S3<br>S2 x S3	S1 x S3<br>S2 x S3	S1 x S3<br>S2 x S3
	ij	S1 x S3	S1 x S3	S2 x S3	S2 x S3	S1 x S3<br>S2 x S3	S1 x S3<br>S2 x S3	–	S1 x S3<br>S2 x S3	–
S_1, S_2 derived	gh	S1 x S3	S1 x S3	S2 x S3	S2 x S3	S1 x S3<br>S2 x S3	S1 x S3<br>S2 x S3	S1 x S3<br>S2 x S3	S1 x S3<br>S2 x S3	S1 x S3<br>S2 x S3
	hj	S1 x S3	S1 x S3	S2 x S3	S2 x S3	S1 x S3<br>S2 x S3	S1 x S3<br>S2 x S3	–	S1 x S3<br>S2 x S3	–

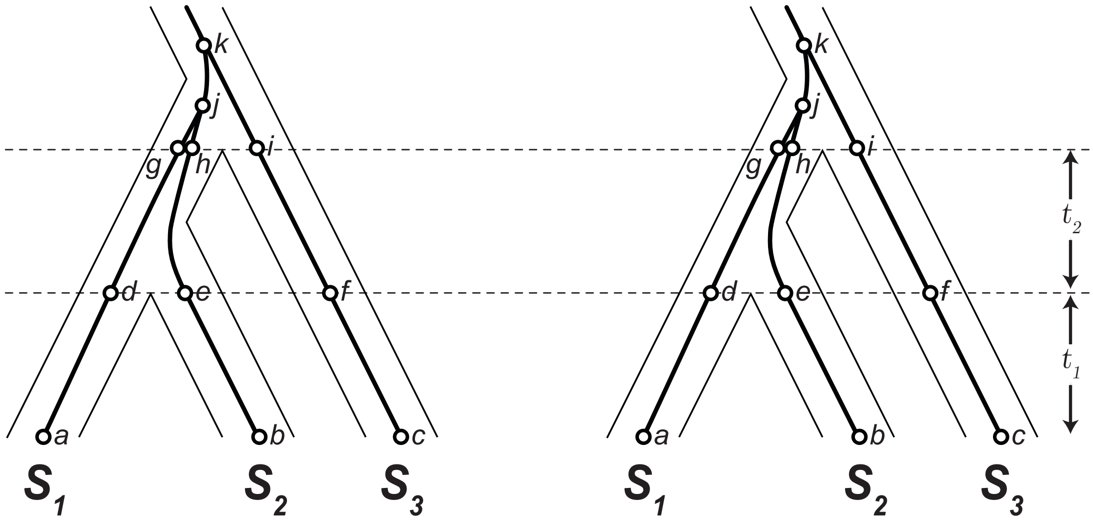

<u>Rows</u>: (S_1, S_2) S_3 anc. <u>Columns</u>: (S_1, S_2) S_3 anc.

		S_1 derived			S_2 derived			S_3 derived			S_1, S_2 derived
		ad	dg	gj	be	eh	hj	cf	fi	ik	jk
S_1 derived	ad	S1 x S2<br>S1 x S3	S1 x S2<br>S1 x S3	S1 x S2<br>S1 x S3	S1 x S2	S1 x S2	S1 x S2	S1 x S3	S1 x S3	S1 x S3	S1 x S3
	dg	S1 x S2<br>S1 x S3	–	–	S1 x S2	–	–	S1 x S3	S1 x S3	S1 x S3	S1 x S3
	gj	S1 x S2<br>S1 x S3	–	–	S1 x S2	–	–	S1 x S3	S1 x S3	–	–
S_2 derived	be	S1 x S2	S1 x S2	S1 x S2	S1 x S2<br>S2 x S3	S1 x S2<br>S2 x S3	S1 x S2<br>S2 x S3	S2 x S3	S2 x S3	S2 x S3	S2 x S3
	eh	S1 x S2	–	–	S1 x S2<br>S2 x S3	–	–	S2 x S3	S2 x S3	S2 x S3	S2 x S3
	hj	S1 x S2	–	–	S1 x S2<br>S2 x S3	–	–	S2 x S3	S2 x S3	–	–
S_3 derived	cf	S1 x S3	S1 x S3	S1 x S3	S2 x S3	S2 x S3	S2 x S3	S1 x S3<br>S2 x S3	S1 x S3<br>S2 x S3	S1 x S3<br>S2 x S3	S1 x S3<br>S2 x S3
	fi	S1 x S3	S1 x S3	S1 x S3	S2 x S3	S2 x S3	S2 x S3	S1 x S3<br>S2 x S3	S1 x S3<br>S2 x S3	S1 x S3<br>S2 x S3	S1 x S3<br>S2 x S3
	ik	S1 x S3	S1 x S3	–	S2 x S3	S2 x S3	–	S1 x S3<br>S2 x S3	S1 x S3<br>S2 x S3	–	–
S_1, S_2 derived	jk	S1 x S3	S1 x S3	–	S2 x S3	S2 x S3	–	S1 x S3<br>S2 x S3	S1 x S3<br>S2 x S3	–	–

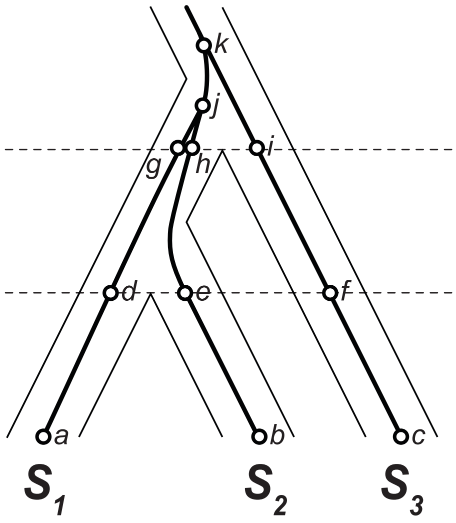

Rows: (S_1, S_2) S_3 anc.

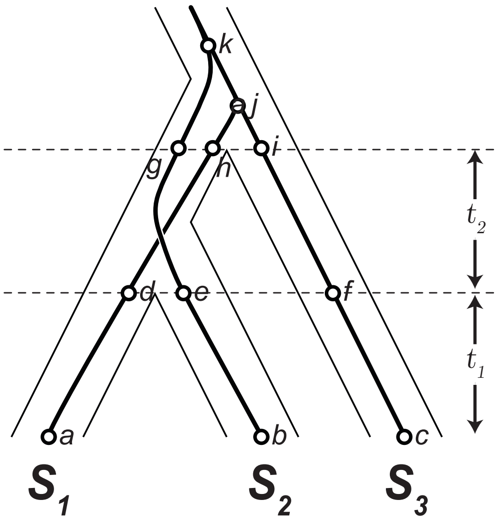

Columns: (S_1, S_3) S_2

		S_1 derived			S_2 derived			S_3 derived			S_1, S_3 derived
		ad	dh	hj	be	eg	gk	cf	fi	ij	jk
S_1 derived	ad	S1 x S2<br>S1 x S3	S1 x S2<br>S1 x S3	S1 x S2<br>S1 x S3	S1 x S2	S1 x S2	S1 x S2	S1 x S3	S1 x S3	S1 x S3	S1 x S2
	dg	S1 x S2<br>S1 x S3	–	–	S1 x S2	–	–	S1 x S3	S1 x S3	S1 x S3	–
	gj	S1 x S2<br>S1 x S3	–	–	S1 x S2	–	–	S1 x S3	S1 x S3	–	–
S_2 derived	be	S1 x S2	S1 x S2	S1 x S2	S1 x S2<br>S2 x S3	S1 x S2<br>S2 x S3	S1 x S2<br>S2 x S3	S2 x S3	S2 x S3	S2 x S3	S1 x S2<br>S2 x S3
	eh	S1 x S2	–	–	S1 x S2<br>S2 x S3	–	–	S2 x S3	S2 x S3	S2 x S3	–
	hj	S1 x S2	–	–	S1 x S2<br>S2 x S3	–	–	S2 x S3	S2 x S3	–	–
S_3 derived	cf	S1 x S3	S1 x S3	S1 x S3	S2 x S3	S2 x S3	S2 x S3	S1 x S3<br>S2 x S3	S1 x S3<br>S2 x S3	S1 x S3<br>S2 x S3	S2 x S3
	fi	S1 x S3	S1 x S3	S1 x S3	S2 x S3	S2 x S3	S2 x S3	S1 x S3<br>S2 x S3	S1 x S3<br>S2 x S3	S1 x S3<br>S2 x S3	S2 x S3
	ik	S1 x S3	S1 x S3	–	S2 x S3	S2 x S3	–	S1 x S3<br>S2 x S3	S1 x S3<br>S2 x S3	–	–
S_1, S_2 derived	jk	S1 x S3	S1 x S3	–	S2 x S3	S2 x S3	–	S1 x S3<br>S2 x S3	S1 x S3<br>S2 x S3	–	–

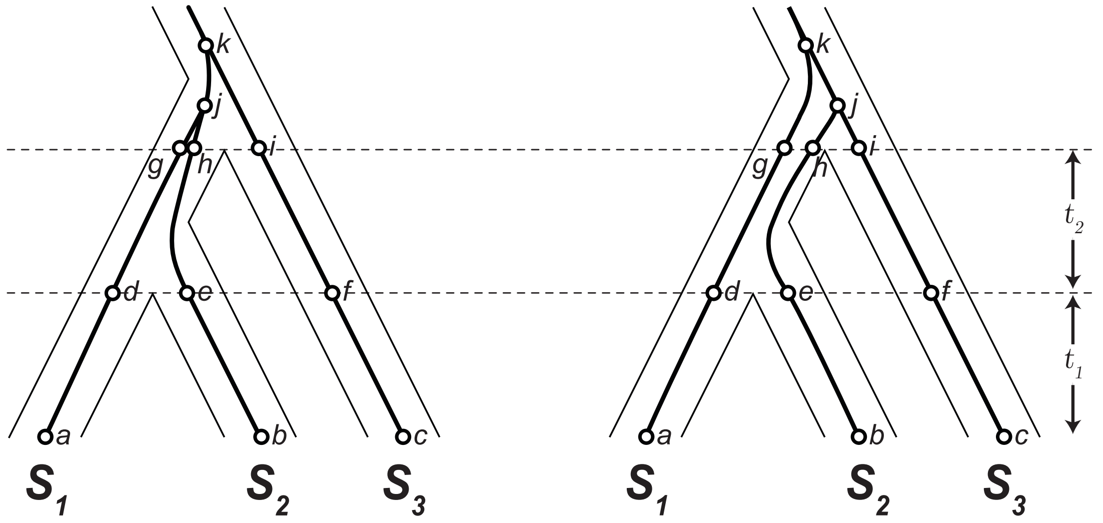

Rows: (S_1, S_2) S_3 anc.   Columns: (S_2, S_3) S_1

		S_1 derived			S_2 derived			S_3 derived			S_2, S_3 derived
		ad	dg	gk	be	eh	hj	cf	fi	ij	jk
S_1 derived	ad	S1 x S2<br>S1 x S3	S1 x S2<br>S1 x S3	S1 x S2<br>S1 x S3	S1 x S2	S1 x S2	S1 x S2	S1 x S3	S1 x S3	S1 x S3	S1 x S2<br>S1 x S3
	dg	S1 x S2<br>S1 x S3	–	–	S1 x S2	–	–	S1 x S3	S1 x S3	S1 x S3	–
	gj	S1 x S2<br>S1 x S3	–	–	S1 x S2	–	–	S1 x S3	S1 x S3	–	–
S_2 derived	be	S1 x S2	S1 x S2	S1 x S2	S1 x S2<br>S2 x S3	S1 x S2<br>S2 x S3	S1 x S2<br>S2 x S3	S2 x S3	S2 x S3	S2 x S3	S1 x S2
	eh	S1 x S2	–	–	S1 x S2<br>S2 x S3	–	–	S2 x S3	S2 x S3	S2 x S3	–
	hj	S1 x S2	–	–	S1 x S2<br>S2 x S3	–	–	S2 x S3	S2 x S3	–	–
S_3 derived	cf	S1 x S3	S1 x S3	S1 x S3	S2 x S3	S2 x S3	S2 x S3	S1 x S3<br>S2 x S3	S1 x S3<br>S2 x S3	S1 x S3<br>S2 x S3	S1 x S3
	fi	S1 x S3	S1 x S3	S1 x S3	S2 x S3	S2 x S3	S2 x S3	S1 x S3<br>S2 x S3	S1 x S3<br>S2 x S3	S1 x S3<br>S2 x S3	S1 x S3
	ik	S1 x S3	S1 x S3	–	S2 x S3	S2 x S3	–	S1 x S3<br>S2 x S3	S1 x S3<br>S2 x S3	–	–
S_1, S_2 derived	jk	S1 x S3	S1 x S3	–	S2 x S3	S2 x S3	–	S1 x S3<br>S2 x S3	S1 x S3<br>S2 x S3	–	–

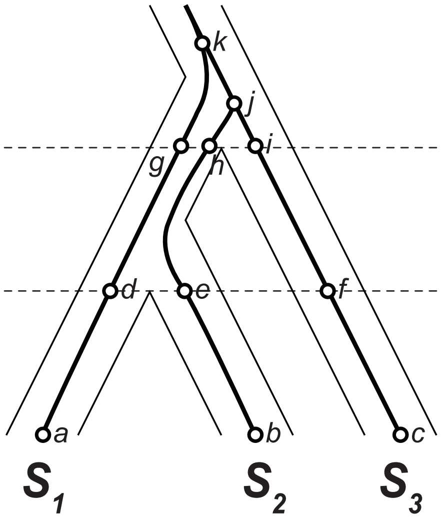

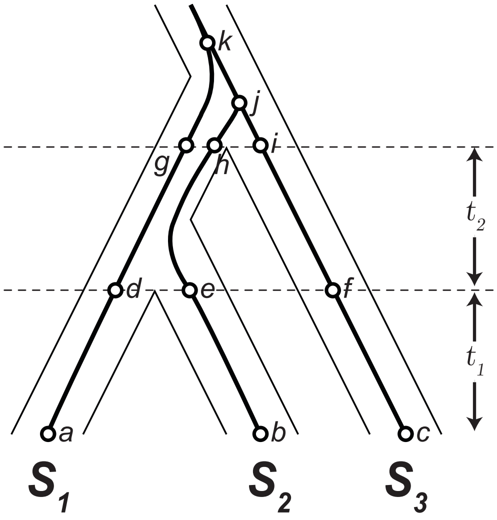

<u>Rows</u>: (S_2, S_3) S_1

<u>Columns</u>: (S_2, S_3) S_1

		S_1 derived			S_2 derived			S_3 derived			S_2, S_3 derived
		ad	dg	gk	be	eh	hj	cf	fi	ij	jk
S_1 derived	ad	S1 x S2<br>S1 x S3	S1 x S2<br>S1 x S3	S1 x S2<br>S1 x S3	S1 x S2	S1 x S2	S1 x S2	S1 x S3	S1 x S3	S1 x S3	S1 x S2<br>S1 x S3
	dg	S1 x S2<br>S1 x S3	–	–	S1 x S2	–	–	S1 x S3	S1 x S3	S1 x S3	–
	gk	S1 x S2<br>S1 x S3	–	–	S1 x S2	–	–	S1 x S3	S1 x S3	–	–
S_2 derived	be	S1 x S2	S1 x S2	S1 x S2	S1 x S2<br>S2 x S3	S1 x S2<br>S2 x S3	S1 x S2<br>S2 x S3	S2 x S3	S2 x S3	S2 x S3	S1 x S2
	eh	S1 x S2	–	–	S1 x S2<br>S2 x S3	–	–	S2 x S3	S2 x S3	S2 x S3	–
	hj	S1 x S2	–	–	S1 x S2<br>S2 x S3	–	–	S2 x S3	S2 x S3	–	–
S_3 derived	cf	S1 x S3	S1 x S3	S1 x S3	S2 x S3	S2 x S3	S2 x S3	S1 x S3<br>S2 x S3	S1 x S3<br>S2 x S3	S1 x S3<br>S2 x S3	S1 x S3
	fi	S1 x S3	S1 x S3	S1 x S3	S2 x S3	S2 x S3	S2 x S3	S1 x S3<br>S2 x S3	S1 x S3<br>S2 x S3	S1 x S3<br>S2 x S3	S1 x S3
	ij	S1 x S3	S1 x S3	–	S2 x S3	S2 x S3	–	S1 x S3<br>S2 x S3	S1 x S3<br>S2 x S3	–	–
S_2, S_3 derived	jk	S1 x S2<br>S1 x S3	–	–	S1 x S2	–	–	S1 x S3	S1 x S3	–	–

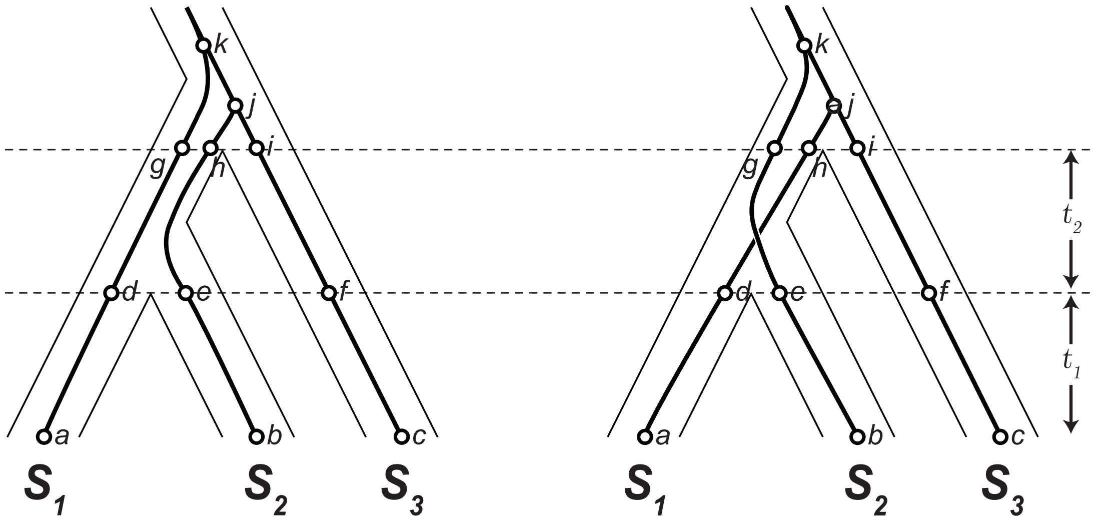

<u>Rows</u>: (S_2, S_3) S_1

<u>Columns</u>: (S_1, S_3) S_2

		S_1 derived			S_2 derived			S_3 derived			S_1, S_3 derived
		ad	dh	hj	be	eg	gk	cf	fi	ij	jk
S_1 derived	ad	S1 x S2<br>S1 x S3	S1 x S2<br>S1 x S3	S1 x S2<br>S1 x S3	S1 x S2	S1 x S2	S1 x S2	S1 x S3	S1 x S3	S1 x S3	S1 x S2
	dg	S1 x S2<br>S1 x S3	–	–	S1 x S2	–	–	S1 x S3	S1 x S3	S1 x S3	–
	gk	S1 x S2<br>S1 x S3	–	–	S1 x S2	–	–	S1 x S3	S1 x S3	–	–
S_2 derived	be	S1 x S2	S1 x S2	S1 x S2	S1 x S2<br>S2 x S3	S1 x S2<br>S2 x S3	S1 x S2<br>S2 x S3	S2 x S3	S2 x S3	S2 x S3	S1 x S2<br>S2 x S3
	eh	S1 x S2	–	–	S1 x S2<br>S2 x S3	–	–	S2 x S3	S2 x S3	S2 x S3	–
	hj	S1 x S2	–	–	S1 x S2<br>S2 x S3	–	–	S2 x S3	S2 x S3	–	–
S_3 derived	cf	S1 x S3	S1 x S3	S1 x S3	S2 x S3	S2 x S3	S2 x S3	S1 x S3<br>S2 x S3	S1 x S3<br>S2 x S3	S1 x S3<br>S2 x S3	S2 x S3
	fi	S1 x S3	S1 x S3	S1 x S3	S2 x S3	S2 x S3	S2 x S3	S1 x S3<br>S2 x S3	S1 x S3<br>S2 x S3	S1 x S3<br>S2 x S3	S2 x S3
	ij	S1 x S3	S1 x S3	–	S2 x S3	S2 x S3	–	S1 x S3<br>S2 x S3	S1 x S3<br>S2 x S3	–	–
S_2, S_3 derived	jk	S1 x S2<br>S1 x S3	–	–	S1 x S2	–	–	S1 x S3	S1 x S3	–	–

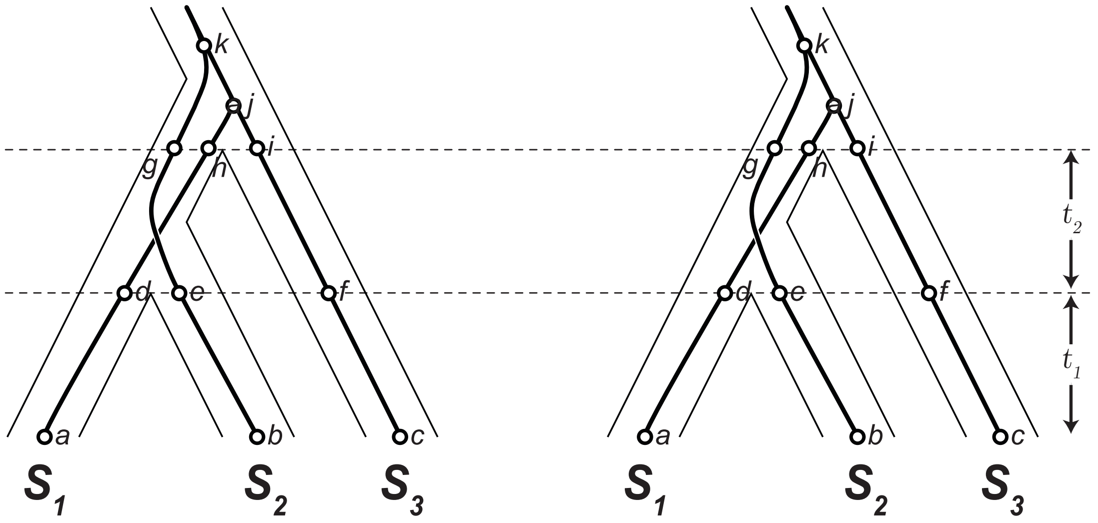

<u>Rows</u>: (S_1, S_3) S_2 <u>Columns</u>: (S_1, S_3) S_2

		S_1 derived			S_2 derived			S_3 derived			S_1, S_3 derived
		ad	dh	hj	be	eg	gk	cf	fi	ij	jk
S_1 derived	ad	S1 x S2<br>S1 x S3	S1 x S2<br>S1 x S3	S1 x S2<br>S1 x S3	S1 x S2	S1 x S2	S1 x S2	S1 x S3	S1 x S3	S1 x S3	S1 x S2
	dh	S1 x S2<br>S1 x S3	–	–	S1 x S2	–	–	S1 x S3	S1 x S3	S1 x S3	–
	hj	S1 x S2<br>S1 x S3	–	–	S1 x S2	–	–	S1 x S3	S1 x S3	–	–
S_2 derived	be	S1 x S2	S1 x S2	S1 x S2	S1 x S2<br>S2 x S3	S1 x S2<br>S2 x S3	S1 x S2<br>S2 x S3	S2 x S3	S2 x S3	S2 x S3	S1 x S2<br>S2 x S3
	eg	S1 x S2	–	–	S1 x S2<br>S2 x S3	–	–	S2 x S3	S2 x S3	S2 x S3	–
	gk	S1 x S2	–	–	S1 x S2<br>S2 x S3	–	–	S2 x S3	S2 x S3	–	–
S_3 derived	cf	S1 x S3	S1 x S3	S1 x S3	S2 x S3	S2 x S3	S2 x S3	S1 x S3<br>S2 x S3	S1 x S3<br>S2 x S3	S1 x S3<br>S2 x S3	S2 x S3
	fi	S1 x S3	S1 x S3	S1 x S3	S2 x S3	S2 x S3	S2 x S3	S1 x S3<br>S2 x S3	S1 x S3<br>S2 x S3	S1 x S3<br>S2 x S3	S2 x S3
	ij	S1 x S3	S1 x S3	–	S2 x S3	S2 x S3	–	S1 x S3<br>S2 x S3	S1 x S3<br>S2 x S3	–	–
S_1, S_3 derived	jk	S1 x S2	–	–	S1 x S2<br>S2 x S3	–	–	S2 x S3	S2 x S3	–	–
