# Supplementary material for: Speciation genes are more likely to have discordant gene trees
Source: Evol Lett. 2018 Aug 8;2(4):281–96. doi: 10.1002/evl3.77 (PMC6121824; doi:10.1002/evl3.77)
Supplement: Supplementary file 14 — Supplementary Material: Appendix 2. Incompatibility matrices with incompatibilities allowed to arise in the same population. [file EVL3-2-281-s013.pdf]

Appendix 2. Incompatibility Matrices with incompatibilities allowed to arise in the same population

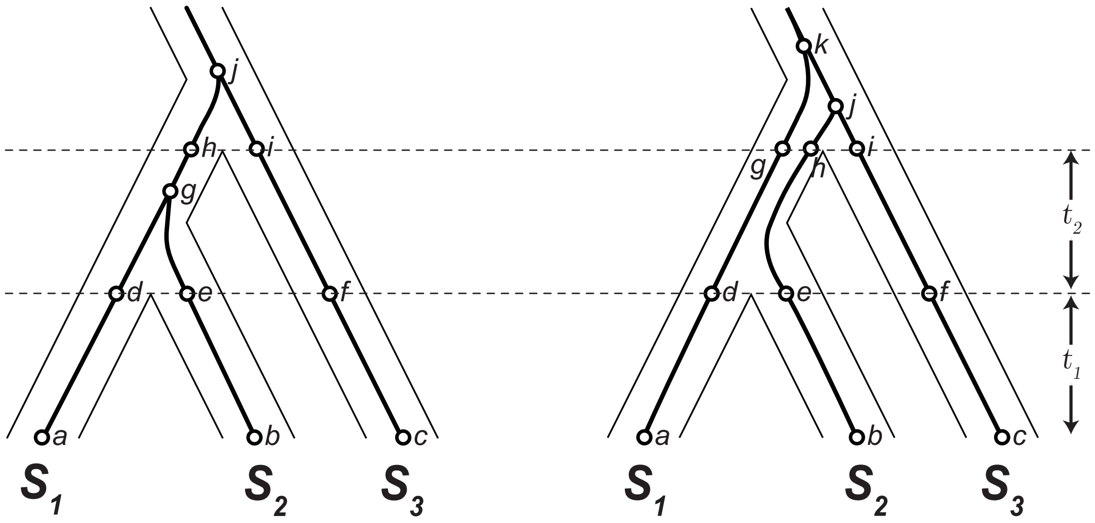

Rows: (S_1, S_2) S_3 int.

Columns: (S_2, S_3) S_1

		S_1 derived			S_2 derived			S_3 derived			S_2, S_3 derived
		ad	dg	gk	be	eh	hj	cf	fi	ij	jk
S_1 derived	ad	S1 x S2<br>S1 x S3	S1 x S2<br>S1 x S3	S1 x S2<br>S1 x S3	S1 x S2	S1 x S2	S1 x S2	S1 x S3	S1 x S3	S1 x S3	S1 x S2<br>S1 x S3
	dg	S1 x S2<br>S1 x S3	S1 x S2<br>S1 x S3	S1 x S2<br>S1 x S3	S1 x S2	S1 x S2	S1 x S2	S1 x S3	S1 x S3	S1 x S3	S1 x S2<br>S1 x S3
S_2 derived	be	S1 x S2	S1 x S2	S1 x S2	S1 x S2<br>S2 x S3	S1 x S2<br>S2 x S3	S1 x S2<br>S2 x S3	S2 x S3	S2 x S3	S2 x S3	S1 x S2
	eg	S1 x S2	S1 x S2	S1 x S2	S1 x S2<br>S2 x S3	S1 x S2<br>S2 x S3	S1 x S2<br>S2 x S3	S2 x S3	S2 x S3	S2 x S3	S1 x S2
S_3 derived	cf	S1 x S3	S1 x S3	S1 x S3	S2 x S3	S2 x S3	S2 x S3	S1 x S3<br>S2 x S3	S1 x S3<br>S2 x S3	S1 x S3<br>S2 x S3	S1 x S3
	fi	S1 x S3	S1 x S3	S1 x S3	S2 x S3	S2 x S3	S2 x S3	S1 x S3<br>S2 x S3	S1 x S3<br>S2 x S3	S1 x S3<br>S2 x S3	S1 x S3
	ij	S1 x S3	S1 x S3	S1 x S3	S2 x S3	S2 x S3	S2 x S3	S1 x S3<br>S2 x S3	S1 x S3<br>S2 x S3	S1 x S3<br>S2 x S3	S1 x S3<br>**A_1** order
S_1, S_2 derived	gh	S1 x S3	S1 x S3<br>**A_2** order	-	S2 x S3	S2 x S3<br>**A_2** order	-	S1 x S3<br>S2 x S3	S1 x S3<br>S2 x S3	S1 x S3<br>S2 x S3	–
	hj	S1 x S3	S1 x S3	S1 x S3<br>**A_3** order	S2 x S3	S2 x S3	S2 x S3<br>**A_4** order	S1 x S3<br>S2 x S3	S1 x S3<br>S2 x S3	S1 x S3<br>S2 x S3	–

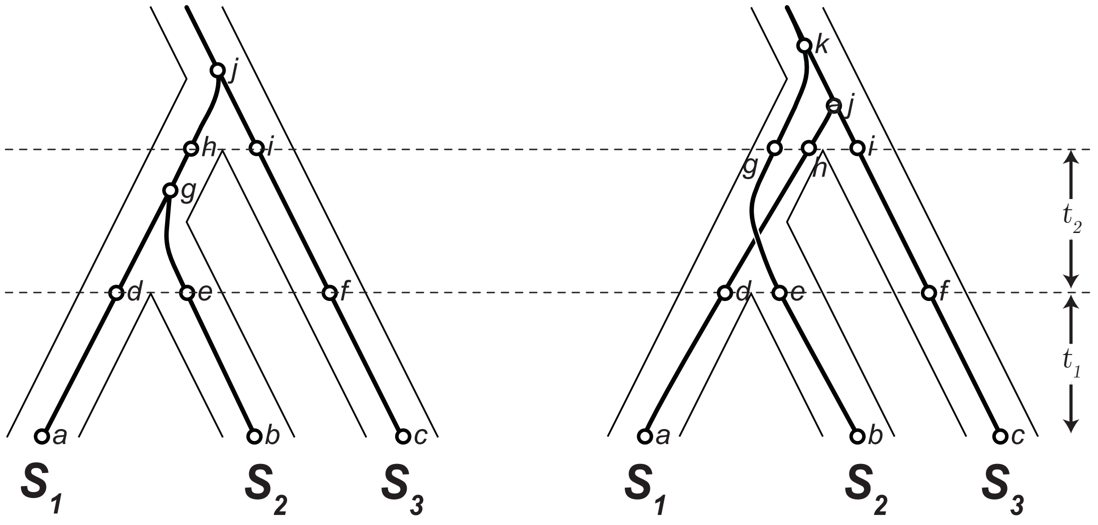

Rows: (S_1, S_2) S_3 int.

Columns: (S_1, S_3) S_2

		S_1 derived			S_2 derived			S_3 derived			S_1, S_3 derived
		ad	dh	hj	be	eg	gk	cf	fi	ij	jk
S_1 derived	ad	S1 x S2<br>S1 x S3	S1 x S2<br>S1 x S3	S1 x S2<br>S1 x S3	S1 x S2	S1 x S2	S1 x S2	S1 x S3	S1 x S3	S1 x S3	S1 x S2
	dg	S1 x S2<br>S1 x S3	S1 x S2<br>S1 x S3	S1 x S2<br>S1 x S3	S1 x S2	S1 x S2	S1 x S2	S1 x S3	S1 x S3	S1 x S3	S1 x S2
S_2 derived	be	S1 x S2	S1 x S2	S1 x S2	S1 x S2<br>S2 x S3	S1 x S2<br>S2 x S3	S1 x S2<br>S2 x S3	S2 x S3	S2 x S3	S2 x S3	S1 x S2<br>S2 x S3
	eg	S1 x S2	S1 x S2	S1 x S2	S1 x S2<br>S2 x S3	S1 x S2<br>S2 x S3	S1 x S2<br>S2 x S3	S2 x S3	S2 x S3	S2 x S3	S1 x S2<br>S2 x S3
S_3 derived	cf	S1 x S3	S1 x S3	S1 x S3	S2 x S3	S2 x S3	S2 x S3	S1 x S3<br>S2 x S3	S1 x S3<br>S2 x S3	S1 x S3<br>S2 x S3	S2 x S3
	fi	S1 x S3	S1 x S3	S1 x S3	S2 x S3	S2 x S3	S2 x S3	S1 x S3<br>S2 x S3	S1 x S3<br>S2 x S3	S1 x S3<br>S2 x S3	S2 x S3
	ij	S1 x S3	S1 x S3	S1 x S3	S2 x S3	S2 x S3	S2 x S3	S1 x S3<br>S2 x S3	S1 x S3<br>S2 x S3	S1 x S3<br>S2 x S3	S2 x S3<br>**A_1** order
S_1, S_2 derived	gh	S1 x S3	S1 x S3<br>**A_2** order	-	S2 x S3	S2 x S3<br>**A_2** order	-	S1 x S3<br>S2 x S3	S1 x S3<br>S2 x S3	S1 x S3<br>S2 x S3	–
	hj	S1 x S3	S1 x S3	S1 x S3[d]<br>**A_4** order	S2 x S3	S2 x S3	S2 x S3<br>**A_3** order	S1 x S3<br>S2 x S3	S1 x S3<br>S2 x S3	S1 x S3<br>S2 x S3	–

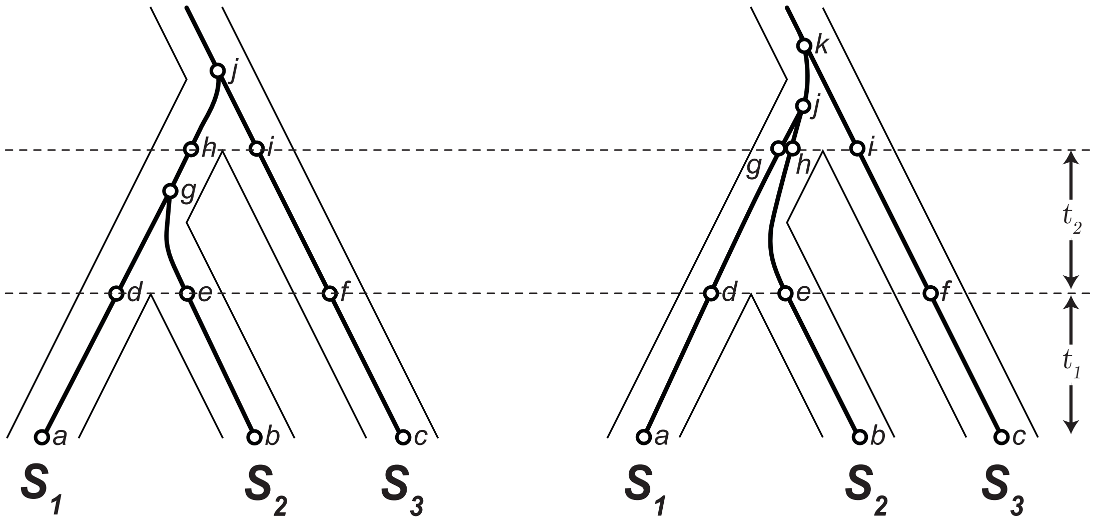

<u>Rows</u>: (S_1, S_2) S_3 int.

<u>Columns</u>: (S_1, S_2) S_3 anc.

		S_1 derived			S_2 derived			S_3 derived			S_1, S_2 derived
		ad	dg	gj	be	eh	hj	cf	fi	ik	jk
S_1 derived	ad	S1 x S2<br>S1 x S3	S1 x S2<br>S1 x S3	S1 x S2<br>S1 x S3	S1 x S2	S1 x S2	S1 x S2	S1 x S3	S1 x S3	S1 x S3	S1 x S3
	dg	S1 x S2<br>S1 x S3	S1 x S2<br>S1 x S3	S1 x S2<br>S1 x S3	S1 x S2	S1 x S2	S1 x S2	S1 x S3	S1 x S3	S1 x S3	S1 x S3
S_2 derived	be	S1 x S2	S1 x S2	S1 x S2	S1 x S2<br>S2 x S3	S1 x S2<br>S2 x S3	S1 x S2<br>S2 x S3	S2 x S3	S2 x S3	S2 x S3	S2 x S3
	eg	S1 x S2	S1 x S2	S1 x S2	S1 x S2<br>S2 x S3	S1 x S2<br>S2 x S3	S1 x S2<br>S2 x S3	S2 x S3	S2 x S3	S2 x S3	S2 x S3
S_3 derived	cf	S1 x S3	S1 x S3	S1 x S3	S2 x S3	S2 x S3	S2 x S3	S1 x S3<br>S2 x S3	S1 x S3<br>S2 x S3	S1 x S3<br>S2 x S3	S1 x S3<br>S2 x S3
	fi	S1 x S3	S1 x S3	S1 x S3	S2 x S3	S2 x S3	S2 x S3	S1 x S3<br>S2 x S3	S1 x S3<br>S2 x S3	S1 x S3<br>S2 x S3	S1 x S3<br>S2 x S3
	ij	S1 x S3	S1 x S3	S1 x S3	S2 x S3	S2 x S3	S2 x S3	S1 x S3<br>S2 x S3	S1 x S3<br>S2 x S3	S1 x S3<br>S2 x S3	S1 x S3<br>S2 x S3
S_1, S_2 derived	gh	S1 x S3	S1 x S3<br>**A_2** order	-	S2 x S3	S2 x S3<br>**A_2** order	-	S1 x S3<br>S2 x S3	S1 x S3<br>S2 x S3	S1 x S3<br>S2 x S3	S1 x S3<br>S2 x S3
	hj	S1 x S3	S1 x S3	S1 x S3<br>**A_4** order	S2 x S3	S2 x S3	S2 x S3<br>**A_4** order	S1 x S3<br>S2 x S3	S1 x S3<br>S2 x S3	S1 x S3<br>S2 x S3	S1 x S3<br>S2 x S3

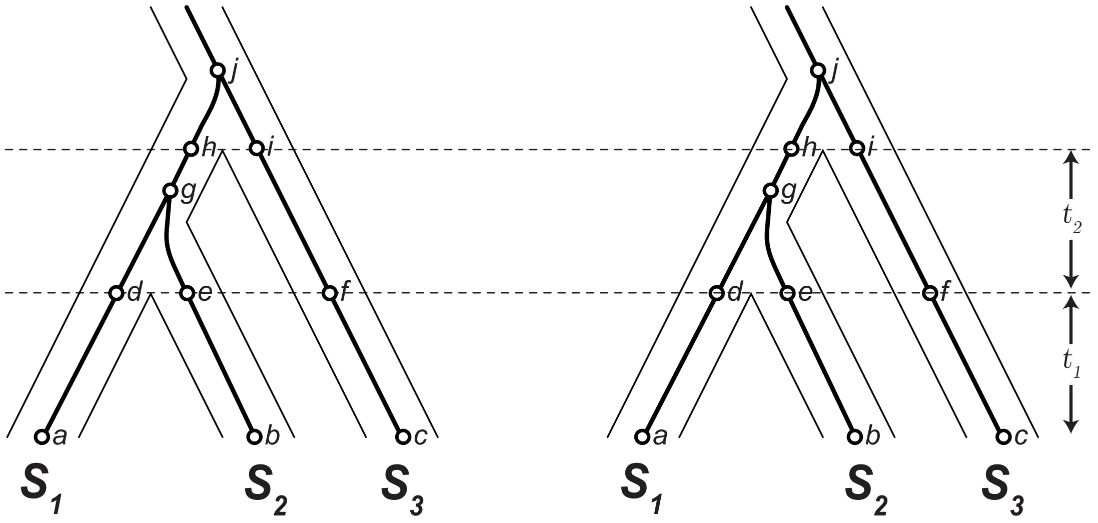

Rows: (S_1, S_2) S_3 int.

Columns: (S_1, S_2) S_3 int.

		S_1 derived		S_2 derived		S_3 derived			S_1, S_2 derived	
		ad	dg	be	eg	cf	fi	ij	gh	hj
S_1 derived	ad	S1 x S2<br>S1 x S3	S1 x S2<br>S1 x S3	S1 x S2	S1 x S2	S1 x S3	S1 x S3	S1 x S3	S1 x S3	S1 x S3
	dg	S1 x S2<br>S1 x S3	S1 x S2<br>S1 x S3	S1 x S2	S1 x S2	S1 x S3	S1 x S3	S1 x S3	S1 x S3<br>A_5 order	S1 x S3
S_2 derived	be	S1 x S2	S1 x S2	S1 x S2<br>S2 x S3	S1 x S2<br>S2 x S3	S2 x S3	S2 x S3	S2 x S3	S2 x S3	S2 x S3
	eg	S1 x S2	S1 x S2	S1 x S2<br>S2 x S3	S1 x S2<br>S2 x S3	S2 x S3	S2 x S3	S2 x S3	S2 x S3<br>A_5 order	S2 x S3
S_3 derived	cf	S1 x S3	S1 x S3	S2 x S3	S2 x S3	S1 x S3<br>S2 x S3	S1 x S3<br>S2 x S3	S1 x S3<br>S2 x S3	S1 x S3<br>S2 x S3	S1 x S3<br>S2 x S3
	fi	S1 x S3	S1 x S3	S2 x S3	S2 x S3	S1 x S3<br>S2 x S3	S1 x S3<br>S2 x S3	S1 x S3<br>S2 x S3	S1 x S3<br>S2 x S3	S1 x S3<br>S2 x S3
	ij	S1 x S3	S1 x S3	S2 x S3	S2 x S3	S1 x S3<br>S2 x S3	S1 x S3<br>S2 x S3	S1 x S3<br>S2 x S3	S1 x S3<br>S2 x S3	S1 x S3<br>S2 x S3
S_1, S_2 derived	gh	S1 x S3	S1 x S3<br>A_5 order	S2 x S3	S2 x S3<br>A_5 order	S1 x S3<br>S2 x S3	S1 x S3<br>S2 x S3	S1 x S3<br>S2 x S3	S1 x S3<br>S2 x S3	S1 x S3<br>S2 x S3
	hj	S1 x S3	S1 x S3	S2 x S3	S2 x S3	S1 x S3<br>S2 x S3	S1 x S3<br>S2 x S3	S1 x S3<br>S2 x S3	S1 x S3<br>S2 x S3	S1 x S3<br>S2 x S3

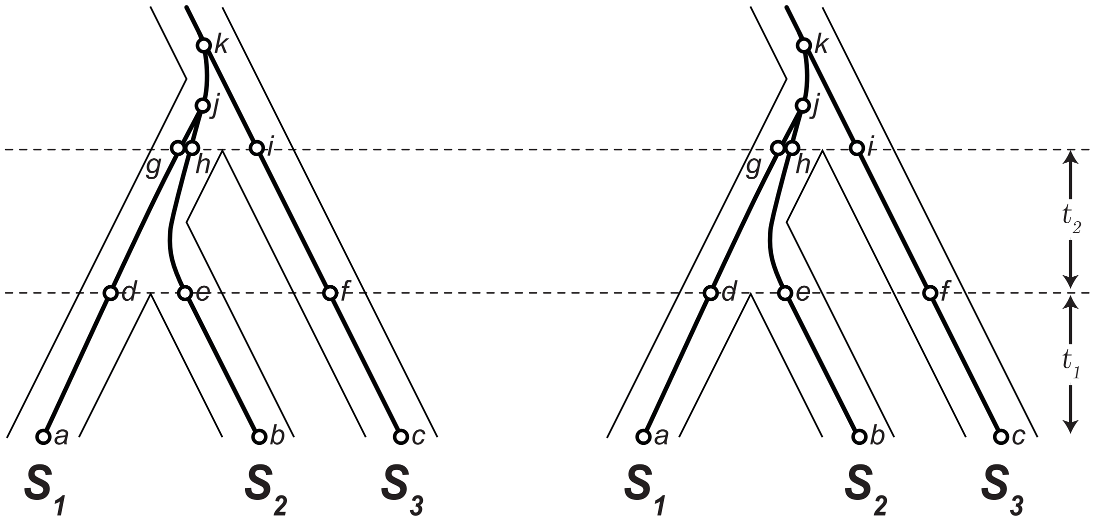

<u>Rows</u>: (S_1, S_2) S_3 anc.

<u>Columns</u>: (S_1, S_2) S_3 anc.

		S_1 derived			S_2 derived			S_3 derived			S_1, S_2 derived
		ad	dg	gj	be	eh	hj	cf	fi	ik	jk
S_1 derived	ad	S1 x S2<br>S1 x S3	S1 x S2<br>S1 x S3	S1 x S2<br>S1 x S3	S1 x S2	S1 x S2	S1 x S2	S1 x S3	S1 x S3	S1 x S3	S1 x S3
	dg	S1 x S2<br>S1 x S3	S1 x S2<br>S1 x S3	S1 x S2<br>S1 x S3	S1 x S2	S1 x S2	S1 x S2	S1 x S3	S1 x S3	S1 x S3	S1 x S3
	gj	S1 x S2<br>S1 x S3	S1 x S2<br>S1 x S3	S1 x S2<br>S1 x S3	S1 x S2	S1 x S2	S1 x S2	S1 x S3	S1 x S3	S1 x S3	S1 x S3<br>**A_6** order
S_2 derived	be	S1 x S2	S1 x S2	S1 x S2	S1 x S2<br>S2 x S3	S1 x S2<br>S2 x S3	S1 x S2<br>S2 x S3	S2 x S3	S2 x S3	S2 x S3	S2 x S3
	eh	S1 x S2	S1 x S2	S1 x S2	S1 x S2<br>S2 x S3	S1 x S2<br>S2 x S3	S1 x S2<br>S2 x S3	S2 x S3	S2 x S3	S2 x S3	S2 x S3
	hj	S1 x S2	S1 x S2	S1 x S2	S1 x S2<br>S2 x S3	S1 x S2<br>S2 x S3	S1 x S2<br>S2 x S3	S2 x S3	S2 x S3	S2 x S3	S2 x S3<br>**A_6** order
S_3 derived	cf	S1 x S3	S1 x S3	S1 x S3	S2 x S3	S2 x S3	S2 x S3	S1 x S3<br>S2 x S3	S1 x S3<br>S2 x S3	S1 x S3<br>S2 x S3	S1 x S3<br>S2 x S3
	fi	S1 x S3	S1 x S3	S1 x S3	S2 x S3	S2 x S3	S2 x S3	S1 x S3<br>S2 x S3	S1 x S3<br>S2 x S3	S1 x S3<br>S2 x S3	S1 x S3<br>S2 x S3
	ik	S1 x S3	S1 x S3	S1 x S3	S2 x S3	S2 x S3	S2 x S3	S1 x S3<br>S2 x S3	S1 x S3<br>S2 x S3	S1 x S3<br>S2 x S3	S1 x S3<br>S2 x S3
S_1, S_2 derived	jk	S1 x S3	S1 x S3	S1 x S3<br>**A_6** order	S2 x S3	S2 x S3	S2 x S3<br>**A_6** order	S1 x S3<br>S2 x S3	S1 x S3<br>S2 x S3	S1 x S3<br>S2 x S3	S1 x S3<br>S2 x S3

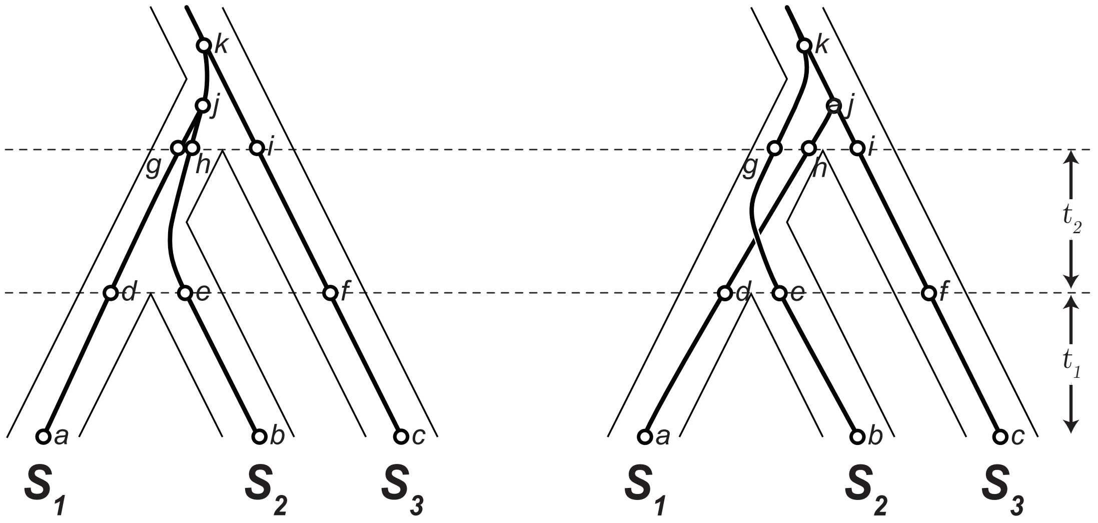

<u>Rows</u>: (S_1, S_2) S_3 anc.   <u>Columns</u>: (S_1, S_3) S_2

		S_1 derived			S_2 derived			S_3 derived			S_1, S_3 derived
		ad	dh	hj	be	eg	gk	cf	fi	ij	jk
S_1 derived	ad	S1 x S2<br>S1 x S3	S1 x S2<br>S1 x S3	S1 x S2<br>S1 x S3	S1 x S2	S1 x S2	S1 x S2	S1 x S3	S1 x S3	S1 x S3	S1 x S2
	dg	S1 x S2<br>S1 x S3	S1 x S2<br>S1 x S3	S1 x S2<br>S1 x S3	S1 x S2	S1 x S2	S1 x S2	S1 x S3	S1 x S3	S1 x S3	S1 x S2
	gj	S1 x S2<br>S1 x S3	S1 x S2<br>S1 x S3	S1 x S2<br>S1 x S3	S1 x S2	S1 x S2	S1 x S2	S1 x S3	S1 x S3	S1 x S3	S1 x S2<br>**A_6** order
S_2 derived	be	S1 x S2	S1 x S2	S1 x S2	S1 x S2<br>S2 x S3	S1 x S2<br>S2 x S3	S1 x S2<br>S2 x S3	S2 x S3	S2 x S3	S2 x S3	S1 x S2<br>S2 x S3
	eh	S1 x S2	S1 x S2	S1 x S2	S1 x S2<br>S2 x S3	S1 x S2<br>S2 x S3	S1 x S2<br>S2 x S3	S2 x S3	S2 x S3	S2 x S3	S1 x S2<br>S2 x S3
	hj	S1 x S2	S1 x S2	S1 x S2	S1 x S2<br>S2 x S3	S1 x S2<br>S2 x S3	S1 x S2<br>S2 x S3	S2 x S3	S2 x S3	S2 x S3	S1 x S2<br>S2 x S3
S_3 derived	cf	S1 x S3	S1 x S3	S1 x S3	S2 x S3	S2 x S3	S2 x S3	S1 x S3<br>S2 x S3	S1 x S3<br>S2 x S3	S1 x S3<br>S2 x S3	S2 x S3
	fi	S1 x S3	S1 x S3	S1 x S3	S2 x S3	S2 x S3	S2 x S3	S1 x S3<br>S2 x S3	S1 x S3<br>S2 x S3	S1 x S3<br>S2 x S3	S2 x S3
	ik	S1 x S3	S1 x S3	S1 x S3	S2 x S3	S2 x S3	S2 x S3	S1 x S3<br>S2 x S3	S1 x S3<br>S2 x S3	S1 x S3<br>S2 x S3	S2 x S3<br>**A_7** order
S_1, S_2 derived	jk	S1 x S3	S1 x S3	S1 x S3<br>**A_6** order	S2 x S3	S2 x S3	S2 x S3<br>**A_7** order	S1 x S3<br>S2 x S3	S1 x S3<br>S2 x S3	S1 x S3<br>S2 x S3	–

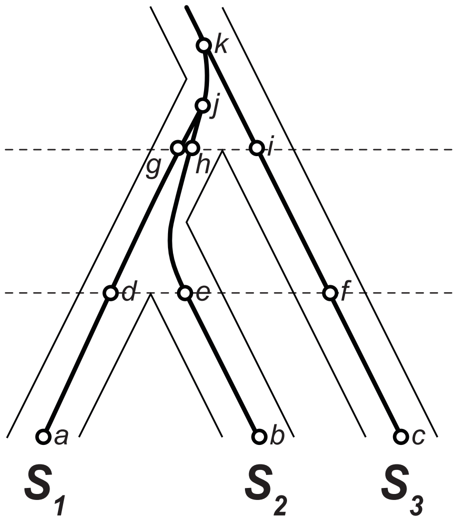

Rows: (S_1, S_2) S_3 anc.

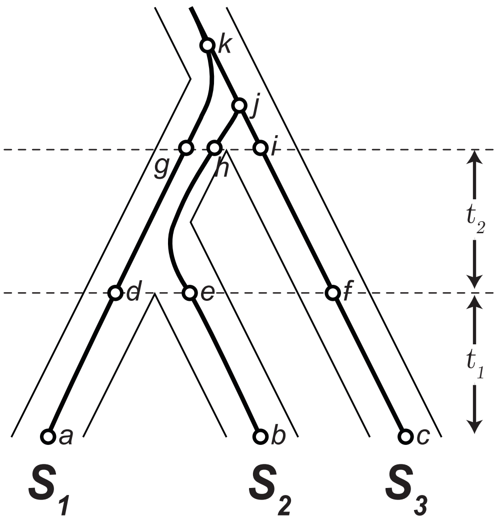

Columns: (S_2, S_3) S_1

		S_1 derived			S_2 derived			S_3 derived			S_2, S_3 derived
		ad	dg	gk	be	eh	hj	cf	fi	ij	jk
S_1 derived	ad	S1 x S2<br>S1 x S3	S1 x S2<br>S1 x S3	S1 x S2<br>S1 x S3	S1 x S2	S1 x S2	S1 x S2	S1 x S3	S1 x S3	S1 x S3	S1 x S2<br>S1 x S3
	dg	S1 x S2<br>S1 x S3	S1 x S2<br>S1 x S3	S1 x S2<br>S1 x S3	S1 x S2	S1 x S2	S1 x S2	S1 x S3	S1 x S3	S1 x S3	S1 x S2<br>S1 x S3
	gj	S1 x S2<br>S1 x S3	S1 x S2<br>S1 x S3	S1 x S2<br>S1 x S3	S1 x S2	S1 x S2	S1 x S2	S1 x S3	S1 x S3	S1 x S3	S1 x S2<br>S1 x S3
S_2 derived	be	S1 x S2	S1 x S2	S1 x S2	S1 x S2<br>S2 x S3	S1 x S2<br>S2 x S3	S1 x S2<br>S2 x S3	S2 x S3	S2 x S3	S2 x S3	S1 x S2
	eh	S1 x S2	S1 x S2	S1 x S2	S1 x S2<br>S2 x S3	S1 x S2<br>S2 x S3	S1 x S2<br>S2 x S3	S2 x S3	S2 x S3	S2 x S3	S1 x S2
	hj	S1 x S2	S1 x S2	S1 x S2	S1 x S2<br>S2 x S3	S1 x S2<br>S2 x S3	S1 x S2<br>S2 x S3	S2 x S3	S2 x S3	S2 x S3	S1 x S2<br>**A_6** order
S_3 derived	cf	S1 x S3	S1 x S3	S1 x S3	S2 x S3	S2 x S3	S2 x S3	S1 x S3<br>S2 x S3	S1 x S3<br>S2 x S3	S1 x S3<br>S2 x S3	S1 x S3
	fi	S1 x S3	S1 x S3	S1 x S3	S2 x S3	S2 x S3	S2 x S3	S1 x S3<br>S2 x S3	S1 x S3<br>S2 x S3	S1 x S3<br>S2 x S3	S1 x S3
	ik	S1 x S3	S1 x S3	S1 x S3	S2 x S3	S2 x S3	S2 x S3	S1 x S3<br>S2 x S3	S1 x S3<br>S2 x S3	S1 x S3<br>S2 x S3	S1 x S3<br>**A_7** order
S_1, S_2 derived	jk	S1 x S3	S1 x S3	S1 x S3<br>**A_7** order	S2 x S3	S2 x S3	S2 x S3<br>**A_6** order	S1 x S3<br>S2 x S3	S1 x S3<br>S2 x S3	S1 x S3<br>S2 x S3	–

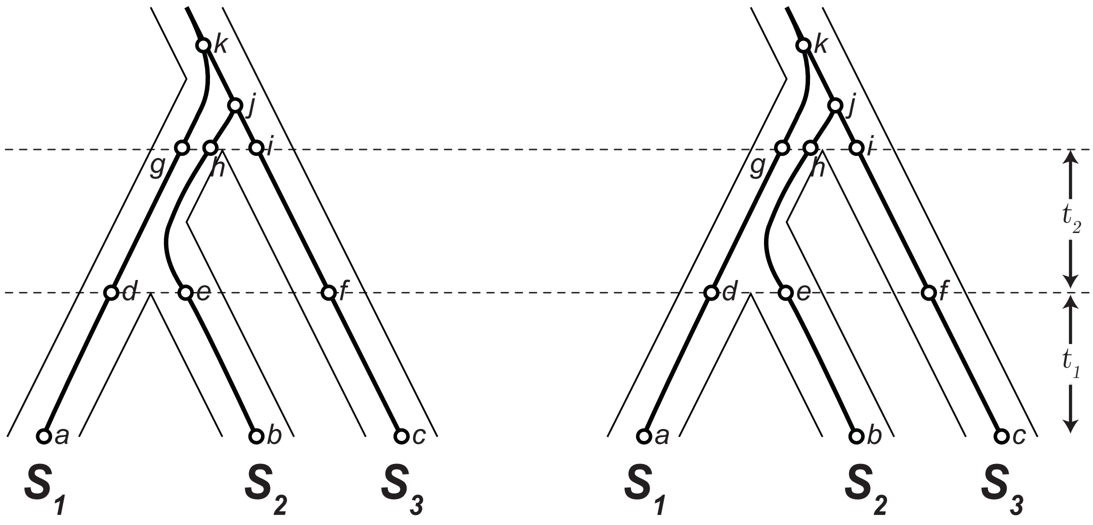

Rows: (S_2, S_3) S_1 Columns: (S_2, S_3) S_1

		S_1 derived			S_2 derived			S_3 derived			S_2, S_3 derived
		ad	dg	gk	be	eh	hj	cf	fi	ij	jk
S_1 derived	ad	S1 x S2<br>S1 x S3	S1 x S2<br>S1 x S3	S1 x S2<br>S1 x S3	S1 x S2	S1 x S2	S1 x S2	S1 x S3	S1 x S3	S1 x S3	S1 x S2<br>S1 x S3
	dg	S1 x S2<br>S1 x S3	S1 x S2<br>S1 x S3	S1 x S2<br>S1 x S3	S1 x S2	S1 x S2	S1 x S2	S1 x S3	S1 x S3	S1 x S3	S1 x S2<br>S1 x S3
	gk	S1 x S2<br>S1 x S3	S1 x S2<br>S1 x S3	S1 x S2<br>S1 x S3	S1 x S2	S1 x S2	S1 x S2	S1 x S3	S1 x S3	S1 x S3	S1 x S2<br>S1 x S3
S_2 derived	be	S1 x S2	S1 x S2	S1 x S2	S1 x S2<br>S2 x S3	S1 x S2<br>S2 x S3	S1 x S2<br>S2 x S3	S2 x S3	S2 x S3	S2 x S3	S1 x S2
	eh	S1 x S2	S1 x S2	S1 x S2	S1 x S2<br>S2 x S3	S1 x S2<br>S2 x S3	S1 x S2<br>S2 x S3	S2 x S3	S2 x S3	S2 x S3	S1 x S2
	hj	S1 x S2	S1 x S2	S1 x S2	S1 x S2<br>S2 x S3	S1 x S2<br>S2 x S3	S1 x S2<br>S2 x S3	S2 x S3	S2 x S3	S2 x S3	S1 x S2<br>**A_6** order
S_3 derived	cf	S1 x S3	S1 x S3	S1 x S3	S2 x S3	S2 x S3	S2 x S3	S1 x S3<br>S2 x S3	S1 x S3<br>S2 x S3	S1 x S3<br>S2 x S3	S1 x S3
	fi	S1 x S3	S1 x S3	S1 x S3	S2 x S3	S2 x S3	S2 x S3	S1 x S3<br>S2 x S3	S1 x S3<br>S2 x S3	S1 x S3<br>S2 x S3	S1 x S3
	ij	S1 x S3	S1 x S3	S1 x S3	S2 x S3	S2 x S3	S2 x S3	S1 x S3<br>S2 x S3	S1 x S3<br>S2 x S3	S1 x S3<br>S2 x S3	S1 x S3<br>**A_6** order
S_2, S_3 derived	jk	S1 x S2<br>S1 x S3	S1 x S2<br>S1 x S3	S1 x S2<br>S1 x S3	S1 x S2	S1 x S2	S1 x S2<br>**A_6** order	S1 x S3	S1 x S3	S1 x S3<br>**A_6** order	S1 x S2<br>S1 x S3

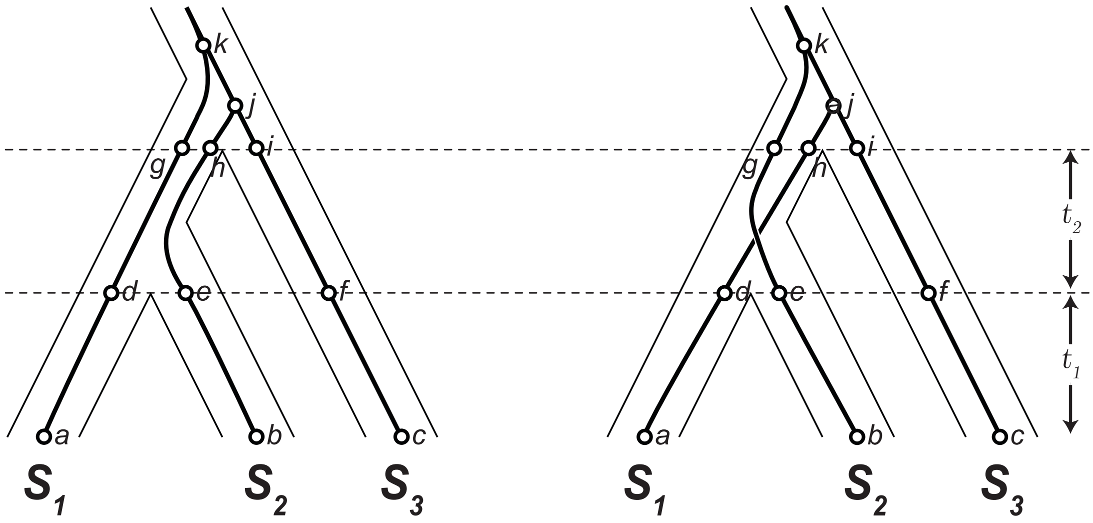

<u>Rows</u>: (S_2, S_3) S_1 <u>Columns</u>: (S_1, S_3) S_2

		S₁ derived			S₂ derived			S₃ derived			S₁, S₃ derived
		ad	dh	hj	be	eg	gk	cf	fi	ij	jk
S₁ derived	ad	S1 x S2<br>S1 x S3	S1 x S2<br>S1 x S3	S1 x S2<br>S1 x S3	S1 x S2	S1 x S2	S1 x S2	S1 x S3	S1 x S3	S1 x S3	S1 x S2
	dg	S1 x S2<br>S1 x S3	S1 x S2<br>S1 x S3	S1 x S2<br>S1 x S3	S1 x S2	S1 x S2	S1 x S2	S1 x S3	S1 x S3	S1 x S3	S1 x S2
	gk	S1 x S2<br>S1 x S3	S1 x S2<br>S1 x S3	S1 x S2<br>S1 x S3	S1 x S2	S1 x S2	S1 x S2	S1 x S3	S1 x S3	S1 x S3	S1 x S2<br>**A_7** order
S₂ derived	be	S1 x S2	S1 x S2	S1 x S2	S1 x S2<br>S2 x S3	S1 x S2<br>S2 x S3	S1 x S2<br>S2 x S3	S2 x S3	S2 x S3	S2 x S3	S1 x S2<br>S2 x S3
	eh	S1 x S2	S1 x S2	S1 x S2	S1 x S2<br>S2 x S3	S1 x S2<br>S2 x S3	S1 x S2<br>S2 x S3	S2 x S3	S2 x S3	S2 x S3	S1 x S2<br>S2 x S3
	hj	S1 x S2	S1 x S2	S1 x S2	S1 x S2<br>S2 x S3	S1 x S2<br>S2 x S3	S1 x S2<br>S2 x S3	S2 x S3	S2 x S3	S2 x S3	S1 x S2<br>S2 x S3
S₃ derived	cf	S1 x S3	S1 x S3	S1 x S3	S2 x S3	S2 x S3	S2 x S3	S1 x S3<br>S2 x S3	S1 x S3<br>S2 x S3	S1 x S3<br>S2 x S3	S2 x S3
	fi	S1 x S3	S1 x S3	S1 x S3	S2 x S3	S2 x S3	S2 x S3	S1 x S3<br>S2 x S3	S1 x S3<br>S2 x S3	S1 x S3<br>S2 x S3	S2 x S3
	ij	S1 x S3	S1 x S3	S1 x S3	S2 x S3	S2 x S3	S2 x S3	S1 x S3<br>S2 x S3	S1 x S3<br>S2 x S3	S1 x S3<br>S2 x S3	S2 x S3<br>**A_6** order
S₂, S₃ derived	jk	S1 x S2<br>S1 x S3	S1 x S2<br>S1 x S3	S1 x S2<br>S1 x S3	S1 x S2	S1 x S2	S1 x S2<br>**A_7** order	S1 x S3	S1 x S3	S1 x S3<br>**A_6** order	–

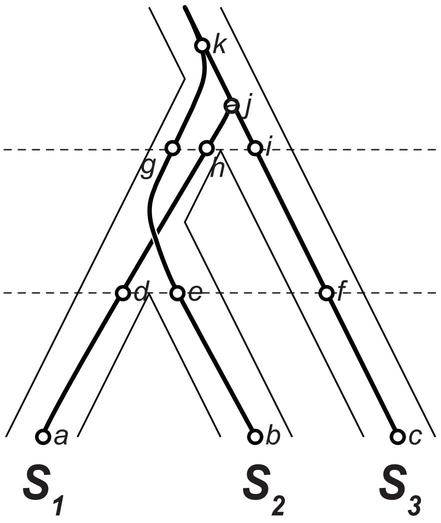

Rows: (S_1, S_3) S_2

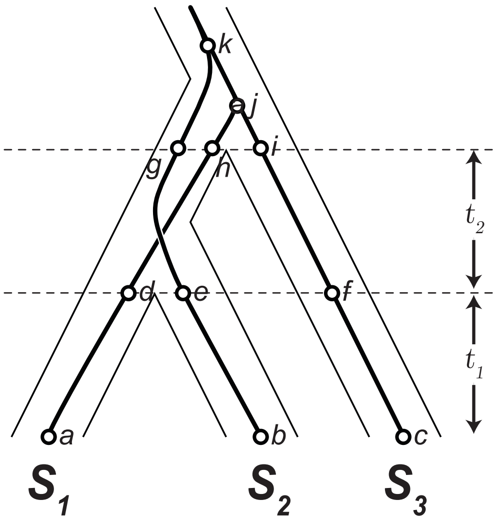

Columns: (S_1, S_3) S_2

		S_1 derived			S_2 derived			S_3 derived			S_1, S_3 derived
		ad	dh	hj	be	eg	gk	cf	fi	ij	jk
S_1 derived	ad	S1 x S2<br>S1 x S3	S1 x S2<br>S1 x S3	S1 x S2<br>S1 x S3	S1 x S2	S1 x S2	S1 x S2	S1 x S3	S1 x S3	S1 x S3	S1 x S2
	dh	S1 x S2<br>S1 x S3	S1 x S2<br>S1 x S3	S1 x S2<br>S1 x S3	S1 x S2	S1 x S2	S1 x S2	S1 x S3	S1 x S3	S1 x S3	S1 x S2
	hj	S1 x S2<br>S1 x S3	S1 x S2<br>S1 x S3	S1 x S2<br>S1 x S3	S1 x S2	S1 x S2	S1 x S2	S1 x S3	S1 x S3	S1 x S3	S1 x S2<br>**A_6** order
S_2 derived	be	S1 x S2	S1 x S2	S1 x S2	S1 x S2<br>S2 x S3	S1 x S2<br>S2 x S3	S1 x S2<br>S2 x S3	S2 x S3	S2 x S3	S2 x S3	S1 x S2<br>S2 x S3
	eg	S1 x S2	S1 x S2	S1 x S2	S1 x S2<br>S2 x S3	S1 x S2<br>S2 x S3	S1 x S2<br>S2 x S3	S2 x S3	S2 x S3	S2 x S3	S1 x S2<br>S2 x S3
	gk	S1 x S2	S1 x S2	S1 x S2	S1 x S2<br>S2 x S3	S1 x S2<br>S2 x S3	S1 x S2<br>S2 x S3	S2 x S3	S2 x S3	S2 x S3	S1 x S2<br>S2 x S3
S_3 derived	cf	S1 x S3	S1 x S3	S1 x S3	S2 x S3	S2 x S3	S2 x S3	S1 x S3<br>S2 x S3	S1 x S3<br>S2 x S3	S1 x S3<br>S2 x S3	S2 x S3
	fi	S1 x S3	S1 x S3	S1 x S3	S2 x S3	S2 x S3	S2 x S3	S1 x S3<br>S2 x S3	S1 x S3<br>S2 x S3	S1 x S3<br>S2 x S3	S2 x S3
	ij	S1 x S3	S1 x S3	S1 x S3	S2 x S3	S2 x S3	S2 x S3	S1 x S3<br>S2 x S3	S1 x S3<br>S2 x S3	S1 x S3<br>S2 x S3	S2 x S3<br>**A_6** order
S_1, S_3 derived	jk	S1 x S2	S1 x S2	S1 x S2<br>**A_6** order	S1 x S2<br>S2 x S3	S1 x S2<br>S2 x S3	S1 x S2<br>S2 x S3	S2 x S3	S2 x S3	S2 x S3<br>**A_6** order	S1 x S2<br>S2 x S3
