# Supplementary material for: Speciation genes are more likely to have discordant gene trees
Source: Evol Lett. 2018 Aug 8;2(4):281–96. doi: 10.1002/evl3.77 (PMC6121824; doi:10.1002/evl3.77)

```
Get[NotebookDirectory[] <> "RelaxedIncompatibilityMatrices.m"]

Get[NotebookDirectory[] <> "RelaxedAncestralMatrices.m"]

{{0, 1, 1, 0, 1, 1, 0, 0, 1, 1}, {1, 0, 1, 1, 0, 1, 0, 0, 1, 1},
 {1, 1, 1, 1, 1, 1, 1, 1, 1, 1}, {0, 1, 1, 0, 1, 1, 0, 0, 1, 1},
 {1, 0, 1, 1, 0, 1, 0, 0, 1, 1}, {1, 1, 1, 1, 1, 1, 1, 1, 1, 1},
 {0, 0, 1, 0, 0, 1, 0, 0, 1, 1}, {0, 0, 1, 0, 0, 1, 0, 0, 1, 1},
 {1, 1, 1, 1, 1, 1, 1, 1, 1, 1}, {1, 1, 1, 1, 1, 1, 1, 1, 1, 1}}

trees = {"(S1,S2)S3 int", "(S1,S2)S3 anc", "(S2,S3)S1", "(S1,S3)S2"};
tree$probabilities = {1 - E^-t2, E^-t2/3, E^-t2/3, E^-t2/3};

BranchLength["(S1,S2)S3 int"] = {t1, q[t2], t1, q[t2], t1, t2, 1, t2 - q[t2], 1};
BranchLength["(S1,S2)S3 anc"] = {t1, t2, 1/3, t1, t2, 1/3, t1, t2, 4/3, 1};
BranchLength["(S2,S3)S1"] = {t1, t2, 4/3, t1, t2, 1/3, t1, t2, 1/3, 1};
BranchLength["(S1,S3)S2"] = {t1, t2, 1/3, t1, t2, 4/3, t1, t2, 1/3, 1};

ParseMatrix[treematrix_, incompatibility_] :=
 Boole[Map[SameQ[#, incompatibility] &, MR[treematrix], {2}]]

GetBranchProducts[tree1_, tree2_, incompatibility_] :=
 If[MatrixQ[MR[StringJoin[tree1, ";", tree2]]],
  Simplify[BranchLength[tree1].ParseMatrix[
     StringJoin[tree1, ";", tree2], incompatibility].BranchLength[tree2]],
  Simplify[BranchLength[tree2].ParseMatrix[StringJoin[tree2, ";", tree1],
     incompatibility].BranchLength[tree1]]]

GetBranchProducts$AncOnly[tree1_, tree2_, incompatibility_] :=
 If[MatrixQ[MR[StringJoin[tree1, ";", tree2]]],
  Simplify[BranchLength[tree1].
    (ParseMatrix[StringJoin[tree1, ";", tree2], incompatibility] *
      RDA[StringJoin[tree1, ";", tree2]]).
    BranchLength[tree2]],
  Simplify[BranchLength[tree2].
    (ParseMatrix[StringJoin[tree2, ";", tree1], incompatibility] *
      RDA[StringJoin[tree2, ";", tree1]]).
    BranchLength[tree1]]]

ParseAMatrix[treematrix_, incompatibility_] :=
 Boole[Map[Not[#] &, PossibleZeroQ[AM[treematrix]], {2}]] *
  Boole[Map[SameQ[#, incompatibility] &, MR[treematrix], {2}]]
```

```
CullDerivedAncestralProducts[tree1_, tree2_, incompatibility_] :=
 If[MatrixQ[AM[StringJoin[tree1, ";", tree2]]],
  Simplify[BranchLength[tree1].ParseAMatrix[
     StringJoin[tree1, ";", tree2], incompatibility].BranchLength[tree2]],
  Simplify[BranchLength[tree2].ParseAMatrix[StringJoin[tree2, ";", tree1],
     incompatibility].BranchLength[tree1]]]

CullDerivedAncestralProducts$AncOnly[tree1_, tree2_, incompatibility_] :=
 If[MatrixQ[AM[StringJoin[tree1, ";", tree2]]],
  Simplify[BranchLength[tree1].
    (ParseAMatrix[StringJoin[tree1, ";", tree2], incompatibility] *
      RDA[StringJoin[tree1, ";", tree2]]).BranchLength[tree2]],
  Simplify[BranchLength[tree2].
    (ParseAMatrix[StringJoin[tree2, ";", tree1], incompatibility] *
      RDA[StringJoin[tree2, ";", tree1]]).BranchLength[tree1]]]

AddDerivedAncestralOrdering[tree1_, tree2_, incompatibility_] :=
 If[MatrixQ[AM[StringJoin[tree1, ";", tree2]]],
  Total[
   ParseAMatrix[StringJoin[tree1, ";", tree2], incompatibility] *
    AM[StringJoin[tree1, ";", tree2]], 2],
  Total[
   ParseAMatrix[StringJoin[tree2, ";", tree1], incompatibility] *
    AM[StringJoin[tree2, ";", tree1]], 2]]

AddDerivedAncestralOrdering$AncOnly[tree1_, tree2_, incompatibility_] :=
 If[MatrixQ[AM[StringJoin[tree1, ";", tree2]]],
  Total[
   RDA[StringJoin[tree1, ";", tree2]] *
    ParseAMatrix[StringJoin[tree1, ";", tree2], incompatibility] *
    AM[StringJoin[tree1, ";", tree2]], 2],
  Total[
   RDA[StringJoin[tree2, ";", tree1]] *
    ParseAMatrix[StringJoin[tree2, ";", tree1], incompatibility] *
    AM[StringJoin[tree2, ";", tree1]], 2]]

fv[x_] = E^-x / (1 - E^-t2);
fτ[t_] = Simplify[Limit[Integrate[fv[x] / (t2 - x), x], x → t] -
    Limit[Integrate[fv[x] / (t2 - x), x], x → 0]];
g[t2_] = FullSimplify[Integrate[t * fv[t] * Integrate[fτ[x], {x, t, t2}], {t, 0, t2}] +
    Integrate[t * fτ[t] * Integrate[fv[x], {x, t, t2}], {t, 0, t2}]];
q[x_] := 1 - (x / (E^x - 1));
```

```
A₁ = 1 - (3 Log[2]) / 4;
A₂ = 1 / 2 (-q[t₂] + t₂) (q[t₂] + t₂);
A₃ = 4 / 3 - (25 Log[2]) / 18;
A₄ = 1 / 9 (3 - Log[4]);
A₅ = 2 * (t₂ - q[t₂]) * g[t₂];
A₆ = 1 / 9 (3 - Log[2]);
A₇ = 4 / 3 - (77 Log[2]) / 72;
```

```
S1xS2$MutationMatrix =
  Outer[GetBranchProducts[##, S1xS2] &, trees, trees] -
   Outer[CullDerivedAncestralProducts[##, S1xS2] &, trees, trees] +
   Outer[AddDerivedAncestralOrdering[##, S1xS2] &, trees, trees];

S1xS2$TreeMatrix = Outer[Times, tree$probabilities, tree$probabilities];

S1xS2$PMatrix = S1xS2$MutationMatrix * S1xS2$TreeMatrix;

S1xS2$MarginalConcordant =
  Simplify[(1/2) (Total[S1xS2$PMatrix[[1]]] + Total[S1xS2$PMatrix[[2]]] +
      Total[S1xS2$PMatrix[[All, 1]]] + Total[S1xS2$PMatrix[[All, 2]]])];
S1xS2$MarginalDiscordant =
  Simplify[(1/2) (Total[S1xS2$PMatrix[[3]]] + Total[S1xS2$PMatrix[[4]]] +
      Total[S1xS2$PMatrix[[All, 3]]] + Total[S1xS2$PMatrix[[All, 4]]])];

$Aborted

$Aborted

S1xS2$ConcordanceRatio = FullSimplify[
  S1xS2$MarginalConcordant / (S1xS2$MarginalConcordant + S1xS2$MarginalDiscordant)]
```

$$\frac{-4\,\text{Log}[2] + 324\,e^{2 t_2}\,(1+t_1)^2 - 72\,e^{t_2}\,(1+t_1)\,(4+3 t_1+3 t_2)}{-36 - 93\,\text{Log}[2] + 324\,e^{2 t_2}\,(1+t_1)^2}$$

```
ContourPlot[S1xS2$ConcordanceRatio / (1 - 2 e^-t2 / 3),
 {t2, 0, 3}, {t1, 0, 3}, PlotLegends → Automatic,
 Contours → {0.55, 0.65, 0.75, 0.85, 0.95, 1, 1.05, 1.15, 1.25, 1.35, 1.45},
 ColorFunctionScaling → False,
 ColorFunction → ColorData[{"ThermometerColors", {1.6, 0.4}}]]
```

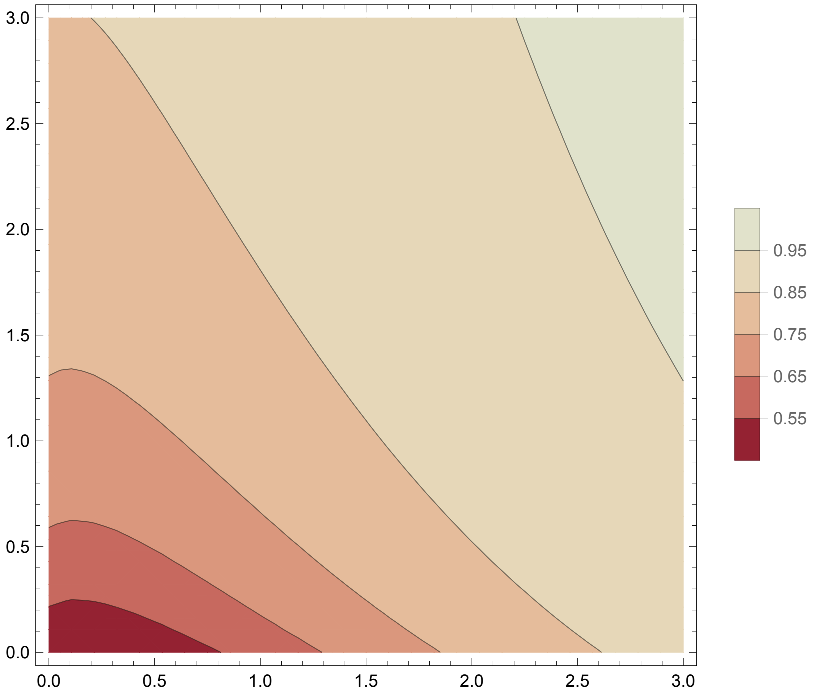


```
Limit[Limit[S1xS2$ConcordanceRatio / (1 - 2 e^-t2 / 3), t1 → 0], t2 → 0]
```

$$\frac{3\,(36 - 4\,\mathrm{Log}[2])}{288 - 93\,\mathrm{Log}[2]}$$

```
N[3 (36 - 4 Log[2]) / (288 - 93 Log[2])]
```

0.445931

```
1 - 0.4459310745427609`
```

0.554069

```
S1xS2$S1xS3$MutationMatrix =
  Outer[GetBranchProducts[##, S1xS2$S1xS3] &, trees, trees] -
   Outer[CullDerivedAncestralProducts[##, S1xS2$S1xS3] &, trees, trees] +
   Outer[AddDerivedAncestralOrdering[##, S1xS2$S1xS3] &, trees, trees];
```

```
S1xS2$S1xS3$TreeMatrix = Outer[Times, tree$probabilities, tree$probabilities];
```

```
S1xS2$S1xS3$PMatrix = S1xS2$S1xS3$MutationMatrix * S1xS2$S1xS3$TreeMatrix;
```

```
S1xS2$S1xS3$MarginalConcordant =
  Simplify[
   (1/2) (Total[S1xS2$S1xS3$PMatrix[[1]]] + Total[S1xS2$S1xS3$PMatrix[[2]]] +
      Total[S1xS2$S1xS3$PMatrix[[All, 1]]] +
      Total[S1xS2$S1xS3$PMatrix[[All, 2]]])];
S1xS2$S1xS3$MarginalDiscordant =
  Simplify[
   (1/2) (Total[S1xS2$S1xS3$PMatrix[[3]]] + Total[S1xS2$S1xS3$PMatrix[[4]]] +
      Total[S1xS2$S1xS3$PMatrix[[All, 3]]] + Total[S1xS2$S1xS3$PMatrix[[All, 4]]])];

S1xS2$S1xS3$ConcordanceRatio = FullSimplify[S1xS2$S1xS3$MarginalConcordant /
   (S1xS2$S1xS3$MarginalConcordant + S1xS2$S1xS3$MarginalDiscordant)]
```

$$1-\frac{2\,e^{-t_2}\left(4+3\,t_1+3\,t_2\right)}{9\,(1+t_1)}$$

```
S1xS2$S2xS3$MutationMatrix =
  Outer[GetBranchProducts[##, S1xS2$S2xS3] &, trees, trees] -
   Outer[CullDerivedAncestralProducts[##, S1xS2$S2xS3] &, trees, trees] +
   Outer[AddDerivedAncestralOrdering[##, S1xS2$S2xS3] &, trees, trees];

S1xS2$S2xS3$TreeMatrix = Outer[Times, tree$probabilities, tree$probabilities];

S1xS2$S2xS3$PMatrix = S1xS2$S2xS3$MutationMatrix * S1xS2$S2xS3$TreeMatrix;

S1xS2$S2xS3$MarginalConcordant =
  Simplify[
   (1/2) (Total[S1xS2$S2xS3$PMatrix[[1]]] + Total[S1xS2$S2xS3$PMatrix[[2]]] +
      Total[S1xS2$S2xS3$PMatrix[[All, 1]]] +
      Total[S1xS2$S2xS3$PMatrix[[All, 2]]])];
S1xS2$S2xS3$MarginalDiscordant =
  Simplify[
   (1/2) (Total[S1xS2$S2xS3$PMatrix[[3]]] + Total[S1xS2$S2xS3$PMatrix[[4]]] +
      Total[S1xS2$S2xS3$PMatrix[[All, 3]]] + Total[S1xS2$S2xS3$PMatrix[[All, 4]]])];

S1xS2$S2xS3$ConcordanceRatio = FullSimplify[S1xS2$S2xS3$MarginalConcordant /
    (S1xS2$S2xS3$MarginalConcordant + S1xS2$S2xS3$MarginalDiscordant)];

S1xS2$S1xS3$ConcordanceRatio == S1xS2$S2xS3$ConcordanceRatio

True
```

```
ContourPlot[S1xS2$S1xS3$ConcordanceRatio / (1 - 2 e^-t2 / 3),
 {t2, 0, 3}, {t1, 0, 3}, PlotLegends → Automatic,
 Contours → {0.55, 0.65, 0.75, 0.85, 0.95, 1, 1.05, 1.15, 1.25, 1.35, 1.45},
 ColorFunctionScaling → False,
 ColorFunction → ColorData[{"ThermometerColors", {1.6, 0.4}}]]
```

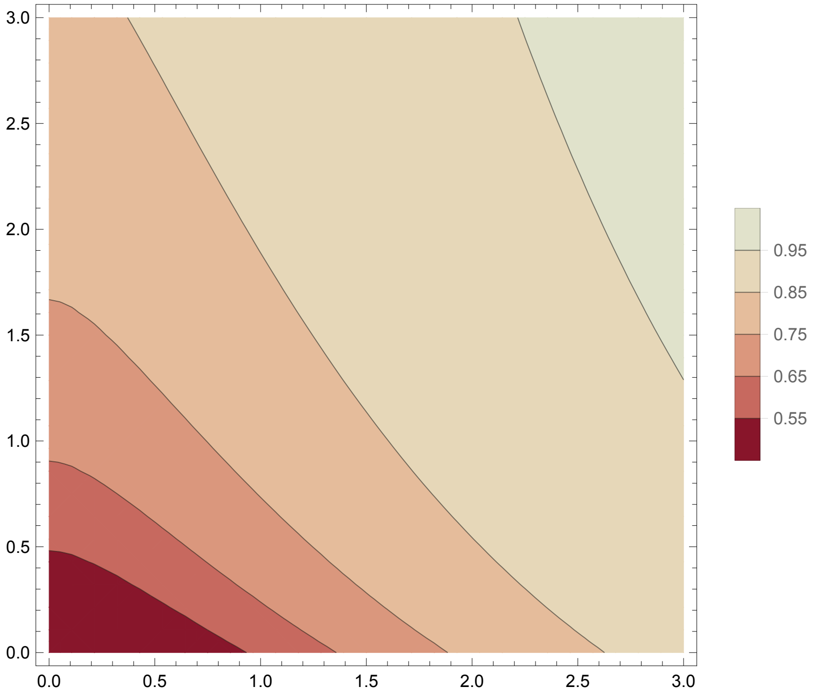


```
Limit[Limit[S1xS2$S1xS3$ConcordanceRatio / (1 - 2 e^-t2 / 3), t1 → 0], t2 → 0]
```

$\frac{1}{3}$

```
S1xS3$MutationMatrix =
  Outer[GetBranchProducts[##, S1xS3] &, trees, trees] -
   Outer[CullDerivedAncestralProducts[##, S1xS3] &, trees, trees] +
   Outer[AddDerivedAncestralOrdering[##, S1xS3] &, trees, trees];

S1xS3$TreeMatrix = Outer[Times, tree$probabilities, tree$probabilities];

S1xS3$PMatrix = S1xS3$MutationMatrix * S1xS3$TreeMatrix;

S1xS3$MarginalConcordant =
  Simplify[(1/2) (Total[S1xS3$PMatrix[[1]]] + Total[S1xS3$PMatrix[[2]]] +
      Total[S1xS3$PMatrix[[All, 1]]] + Total[S1xS3$PMatrix[[All, 2]]])];
S1xS3$MarginalDiscordant =
  Simplify[(1/2) (Total[S1xS3$PMatrix[[3]]] + Total[S1xS3$PMatrix[[4]]] +
      Total[S1xS3$PMatrix[[All, 3]]] + Total[S1xS3$PMatrix[[All, 4]]])];
```

```
S1xS3$ConcordanceRatio = Simplify[S1xS3$MarginalConcordant /
    (S1xS3$MarginalConcordant + S1xS3$MarginalDiscordant)];

S2xS3$MutationMatrix =
  Outer[GetBranchProducts[##, S2xS3] &, trees, trees] -
   Outer[CullDerivedAncestralProducts[##, S2xS3] &, trees, trees] +
   Outer[AddDerivedAncestralOrdering[##, S2xS3] &, trees, trees];

S2xS3$TreeMatrix = Outer[Times, tree$probabilities, tree$probabilities];

S2xS3$PMatrix = S2xS3$MutationMatrix * S2xS3$TreeMatrix;

S2xS3$MarginalConcordant =
  Simplify[(1/2) (Total[S2xS3$PMatrix[[1]]] + Total[S2xS3$PMatrix[[2]]] +
      Total[S2xS3$PMatrix[[All, 1]]] + Total[S2xS3$PMatrix[[All, 2]]])];
S2xS3$MarginalDiscordant =
  Simplify[(1/2) (Total[S2xS3$PMatrix[[3]]] + Total[S2xS3$PMatrix[[4]]] +
      Total[S2xS3$PMatrix[[All, 3]]] + Total[S2xS3$PMatrix[[All, 4]]])];

S2xS3$ConcordanceRatio = Simplify[S2xS3$MarginalConcordant /
    (S2xS3$MarginalConcordant + S2xS3$MarginalDiscordant)];

S1xS3$ConcordanceRatio == S2xS3$ConcordanceRatio

True
```

```
ContourPlot[S1xS3$ConcordanceRatio / (1 - 2 e^-t2 / 3),
 {t2, 0, 3}, {t1, 0, 3}, PlotLegends → Automatic,
 PlotRange → All,
 Contours → {0.55, 0.65, 0.75, 0.85, 0.95, 1, 1.05, 1.15, 1.25, 1.35, 1.45},
 ColorFunctionScaling → False,
 ColorFunction → ColorData[{"ThermometerColors", {1.6, 0.4}}]]
```

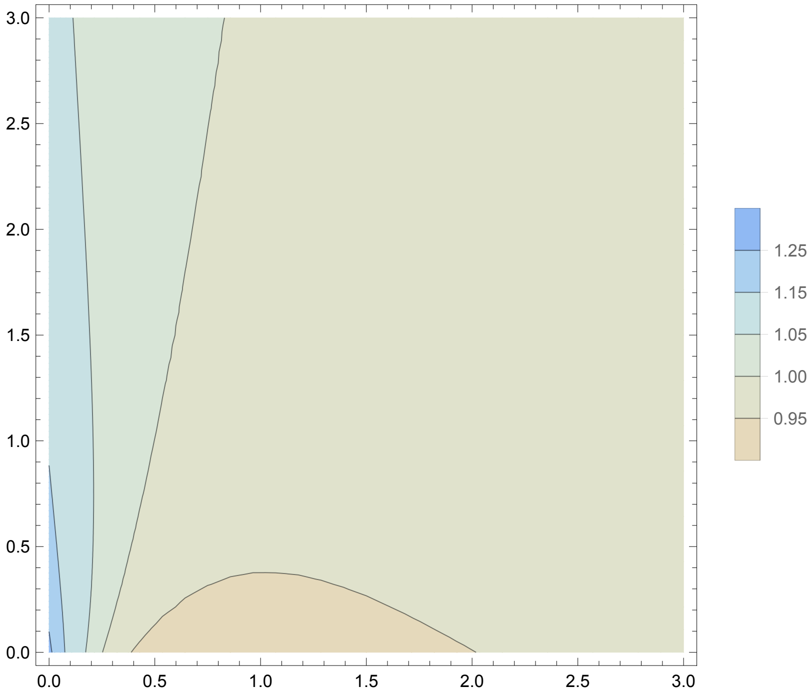


```
Limit[Limit[S1xS3$ConcordanceRatio / (1 - 2 e^-t2 / 3), t1 → 0], t2 → 0]
```

$$\frac{-252 + 89\,\text{Log}[2]}{-192 + 62\,\text{Log}[2]}$$

```
S1xS3$S2xS3$MutationMatrix =
  Outer[GetBranchProducts[##, S1xS3$S2xS3] &, trees, trees] -
   Outer[CullDerivedAncestralProducts[##, S1xS3$S2xS3] &, trees, trees] +
   Outer[AddDerivedAncestralOrdering[##, S1xS3$S2xS3] &, trees, trees];

S1xS3$S2xS3$TreeMatrix = Outer[Times, tree$probabilities, tree$probabilities];

S1xS3$S2xS3$PMatrix = S1xS3$S2xS3$MutationMatrix * S1xS3$S2xS3$TreeMatrix;
```

```
S1xS3$S2xS3$MarginalConcordant =
  Simplify[
   (1/2) (Total[S1xS3$S2xS3$PMatrix[[1]]] + Total[S1xS3$S2xS3$PMatrix[[2]]] +
      Total[S1xS3$S2xS3$PMatrix[[All, 1]]] +
      Total[S1xS3$S2xS3$PMatrix[[All, 2]]])];
S1xS3$S2xS3$MarginalDiscordant =
  Simplify[
   (1/2) (Total[S1xS3$S2xS3$PMatrix[[3]]] + Total[S1xS3$S2xS3$PMatrix[[4]]] +
      Total[S1xS3$S2xS3$PMatrix[[All, 3]]] + Total[S1xS3$S2xS3$PMatrix[[All, 4]]])];

S1xS3$S2xS3$ConcordanceRatio = Simplify[S1xS3$S2xS3$MarginalConcordant /
    (S1xS3$S2xS3$MarginalConcordant + S1xS3$S2xS3$MarginalDiscordant)];
```

ContourPlot[S1xS3$S2xS3$ConcordanceRatio / $\left(1 - \frac{2 e^{-t_2}}{3}\right)$,

{t_2, 0, 3}, {t_1, 0, 3}, PlotLegends → Automatic,
PlotRange → All,
Contours → {0.55, 0.65, 0.75, 0.85, 0.95, 1, 1.05, 1.15, 1.25, 1.35, 1.45, 1.55},
ColorFunctionScaling → False,
ColorFunction → ColorData[{"ThermometerColors", {1.6, 0.4}}]]

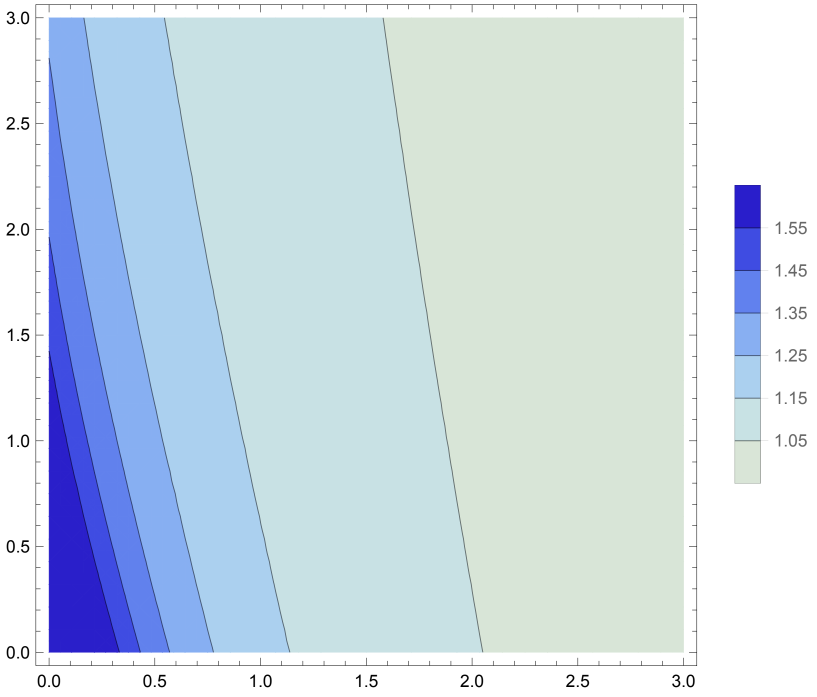

Limit[Limit[S1xS3$S2xS3$ConcordanceRatio / $\left(1 - \frac{2 e^{-t_2}}{3}\right)$, t_1 → 0], t_2 → 0]

$\frac{7}{3}$

```
N[7/3]
```

2.33333

```
ContourPlot[x + y, {x, 0.5, 0.55}, {y, 0, 1.05},
 PlotLegends → Placed[Automatic, Right], ColorFunctionScaling → False,
 Contours → {0.55, 0.65, 0.75, 0.85, 0.95, 1.05, 1.15, 1.25, 1.35, 1.45, 1.55},
 ColorFunction → ColorData[{"ThermometerColors", {1.6, 0.4}}]]
```

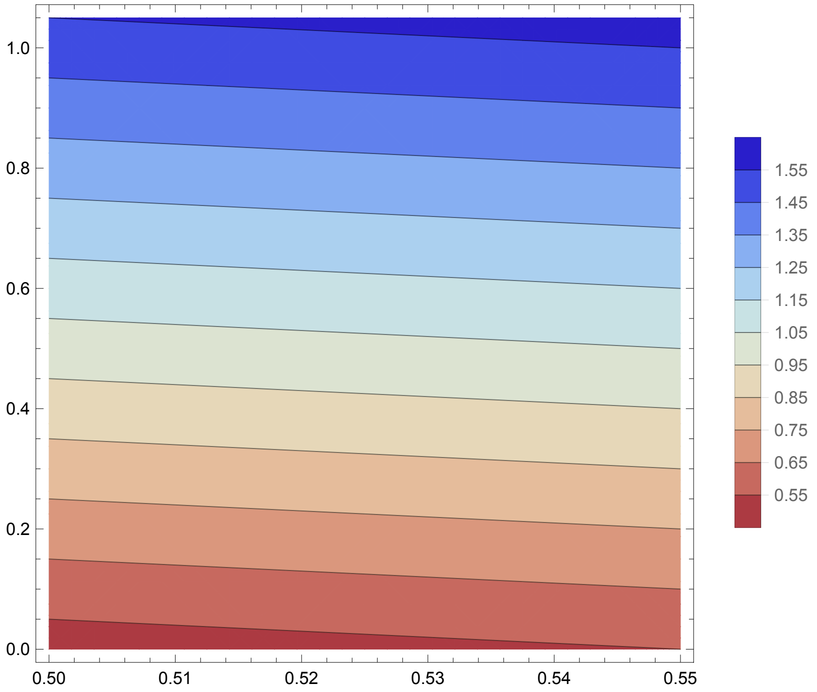


```
TotalP = Total[S1xS2$PMatrix, 2] + Total[S1xS3$PMatrix, 2] +
   Total[S2xS3$PMatrix, 2] + Total[S1xS3$S2xS3$PMatrix, 2] +
   Total[S1xS2$S1xS3$PMatrix, 2] + Total[S1xS2$S2xS3$PMatrix, 2];

P$concord = Simplify[(S1xS2$ConcordanceRatio * Total[S1xS2$PMatrix, 2] +
       S1xS3$ConcordanceRatio * Total[S1xS3$PMatrix, 2] +
       S2xS3$ConcordanceRatio * Total[S2xS3$PMatrix, 2] +
       S1xS3$S2xS3$ConcordanceRatio * Total[S1xS3$S2xS3$PMatrix, 2] +
       S1xS2$S1xS3$ConcordanceRatio * Total[S1xS2$S1xS3$PMatrix, 2] +
       S1xS2$S2xS3$ConcordanceRatio * Total[S1xS2$S2xS3$PMatrix, 2]) /
    TotalP];
```

```
ContourPlot[P$concord / (1 - 2 ⅇ^(-t₂)/3), {t₂, 0, 3}, {t₁, 0, 3}, PlotLegends → Automatic,
 ColorFunction → ColorData[{"RedBlueTones", {1.05, 0.95}}],
 PlotRange → All, ColorFunctionScaling → False,
 Contours → {0.95, 0.96, 0.97,  0.98, 0.99, 1.00, 1.01, 1.02, 1.03, 1.04}]
```

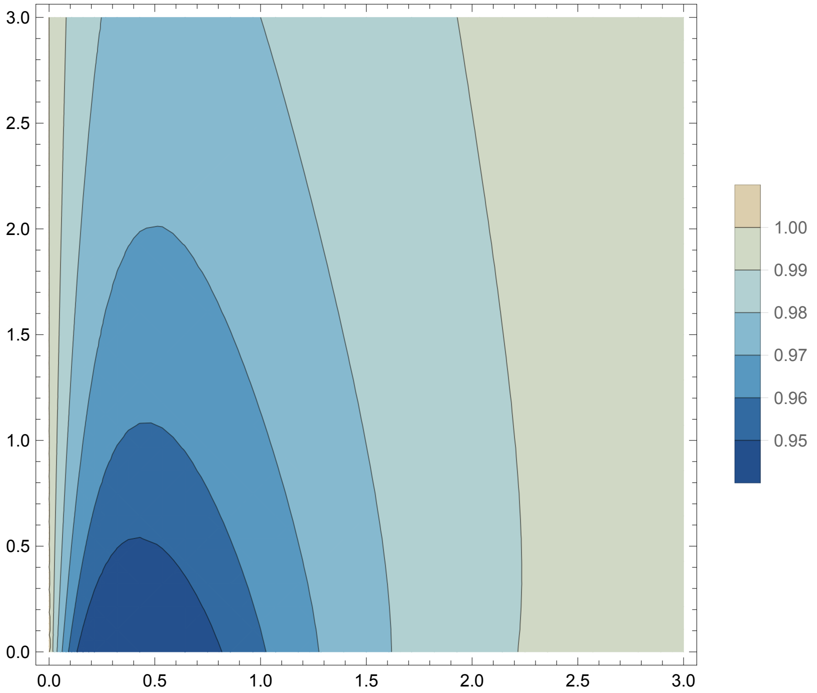


```
FindMinimum[{P$concord / (1 - 2 ⅇ^(-t₂)/3), t₁ >= 0 && t₂ >= 0}, {{t₁, 1}, {t₂, 1}}]
```

$\{0.931998, \{t_1 \to 1.86898 \times 10^{-6}, t_2 \to 0.359363\}\}$

```
ContourPlot[x + y, {x, .945, 1}, {y, 0, .06},
 PlotLegends → Placed[Automatic, Right], ColorFunctionScaling → False,
 Contours → {0.95, 0.96, 0.97, 0.98, 0.99, 1.00, 1.01, 1.02, 1.03, 1.04, 1.05},
 ColorFunction → ColorData[{"RedBlueTones", {1.05, 0.95}}]]
```

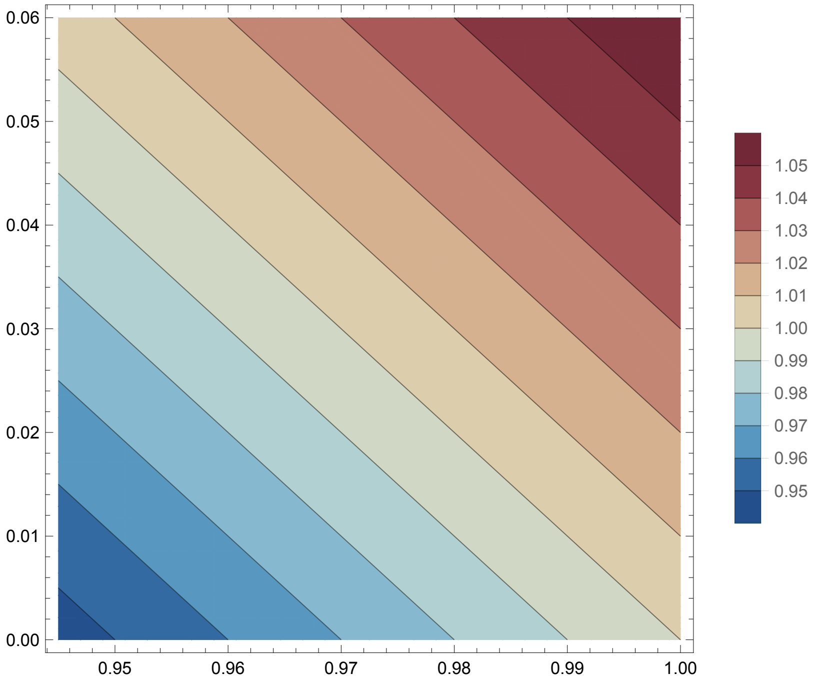

```
ContourPlot[(Total[S1xS2$S2xS3$PMatrix, 2] + Total[S1xS2$S1xS3$PMatrix, 2] +
    Total[S1xS3$S2xS3$PMatrix, 2]) / TotalP, {t2, 0, 3}, {t1, 0, 3},
 ColorFunctionScaling → False, PlotRange → All,
 Contours → {0.05, 0.1, 0.15, 0.2, 0.25, 0.3, 1/3, 0.35, 0.4, 0.45, 0.5},
 ColorFunction → ColorData[{"GrayTones", {0.5, 0}}],
 PlotLegends → Automatic]
```

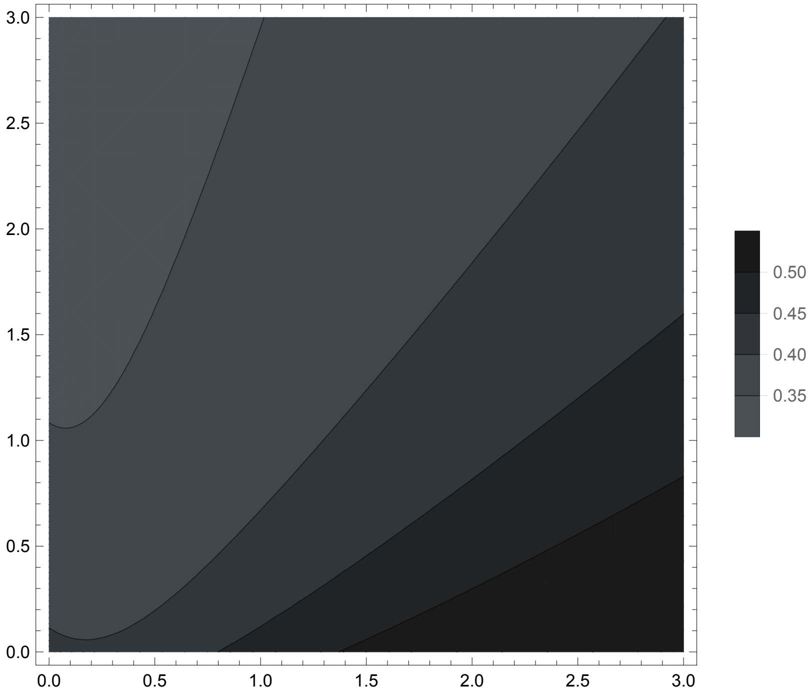

```
ContourPlot[(Total[S1xS2$PMatrix, 2] + Total[S1xS2$S1xS3$PMatrix, 2] +
    Total[S1xS2$S2xS3$PMatrix, 2]) / TotalP, {t₂, 0, 3}, {t₁, 0, 3},
 ColorFunctionScaling → False,
 Contours → {0.05, 0.1, 0.15, 0.2, 0.25, 0.3, 0.35, 0.4, 0.45, 0.5},
 ColorFunction → ColorData[{"GrayTones", {0.5, 0}}]]
```

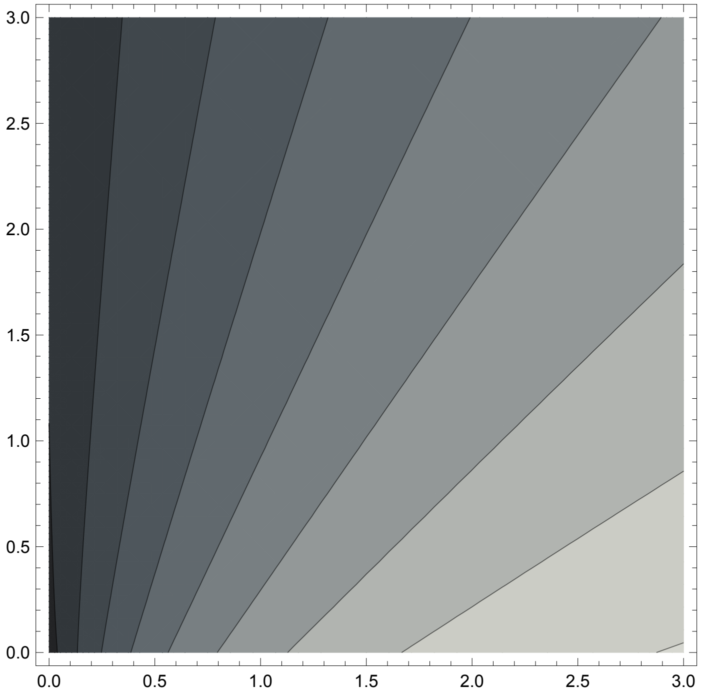

```
ContourPlot[Total[S1xS3$PMatrix, 2] / TotalP, {t₂, 0, 3}, {t₁, 0, 3},
 ColorFunctionScaling → False,
 Contours → {0.05, 0.1, 0.15, 0.2, 0.25, 0.3, 0.35, 0.4, 0.45, 0.5},
 ColorFunction → ColorData[{"GrayTones", {0.5, 0}}]]
```

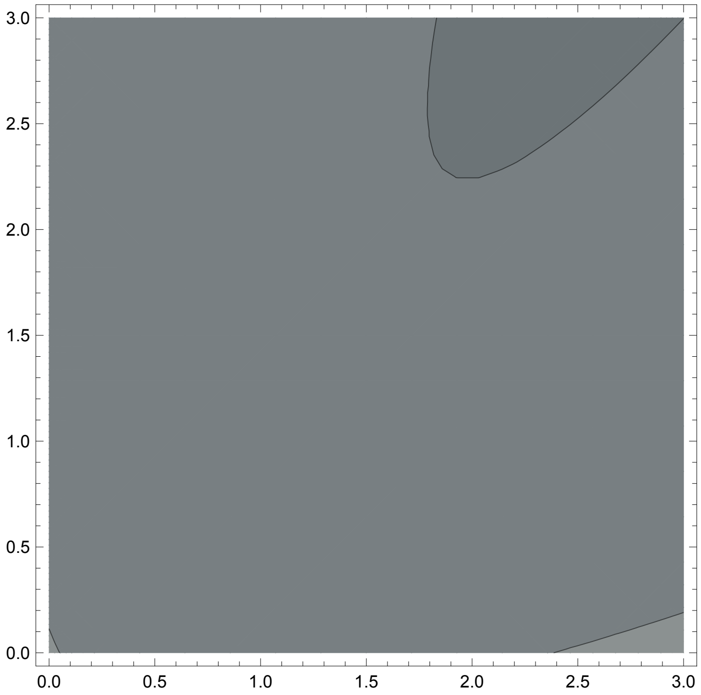

```
ContourPlot[(Total[S1xS3$S2xS3$PMatrix, 2]) / TotalP, {t₂, 0, 3}, {t₁, 0, 3},
 ColorFunctionScaling → False,
 Contours → {0.05, 0.1, 0.15, 0.2, 0.25, 0.3, 0.35, 0.4, 0.45, 0.5},
 ColorFunction → ColorData[{"GrayTones", {0.5, 0}}]]
```

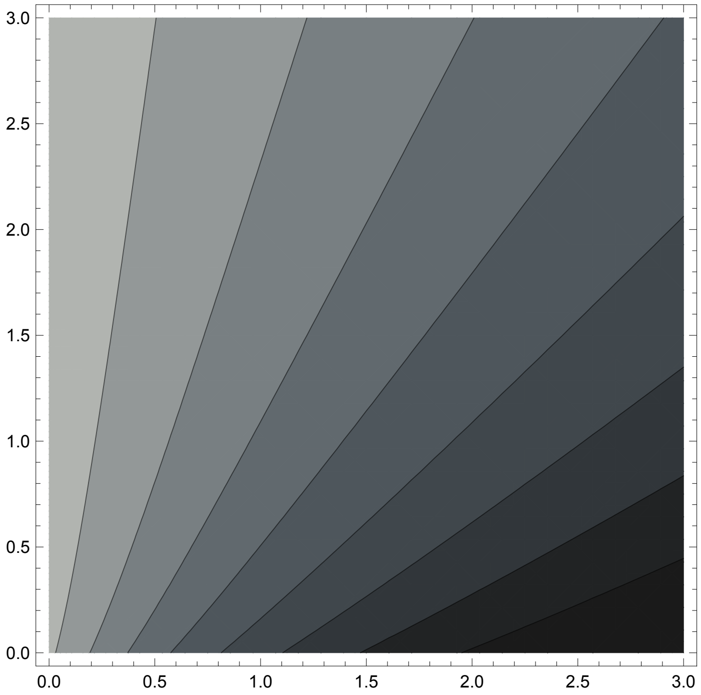

```
ContourPlot[Total[S1xS2$S2xS3$PMatrix, 2] / TotalP, {t₂, 0, 3}, {t₁, 0, 3},
 ColorFunctionScaling → False,
 Contours → {0.05, 0.1, 0.15, 0.2, 0.25, 0.3, 0.35, 0.4, 0.45, 0.5},
 ColorFunction → ColorData[{"GrayTones", {0.5, 0}}]]
```

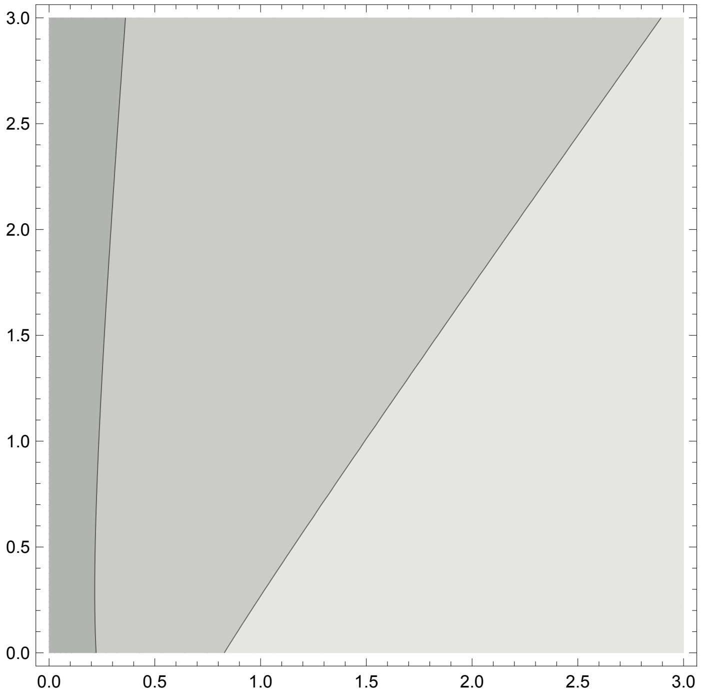

```
ContourPlot[Total[S1xS3$S2xS3$PMatrix, 2] / TotalP, {t₂, 0, 3}, {t₁, 0, 3},
 ColorFunctionScaling → False,
 Contours → {0.1, 0.15, 0.2, 0.25, 0.3, 1 / 3, 0.35, 0.4, 0.45, 0.5},
 ColorFunction → ColorData[{"GrayTones", {0.6, 0}}],
 PlotLegends → Automatic, FrameLabel → Automatic]
```

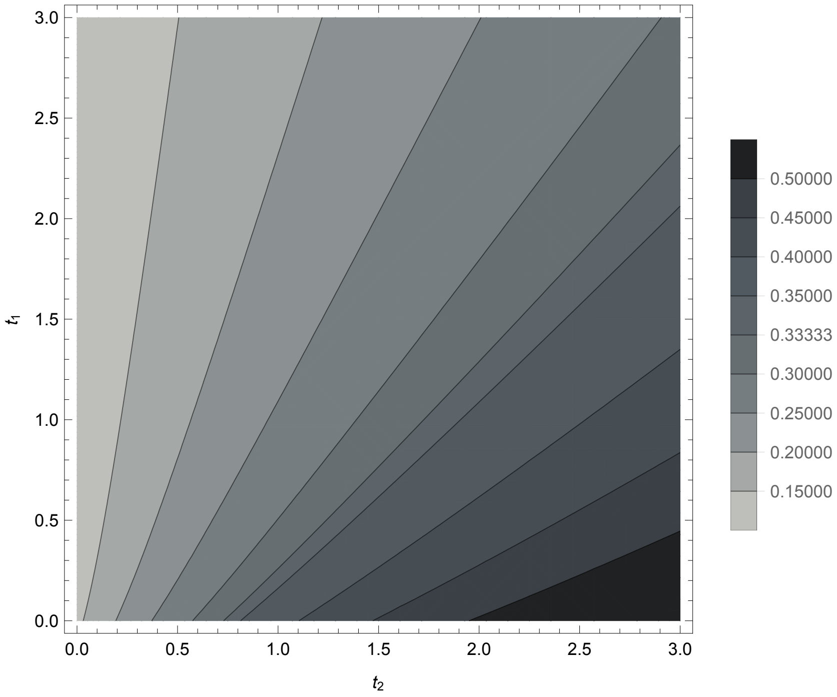

```
ContourPlot[Total[S1xS2$S1xS3$PMatrix, 2] / TotalP, {t2, 0, 3}, {t1, 0, 3},
 ColorFunctionScaling → False,
 Contours → 5,
 ColorFunction → ColorData[{"GrayTones", {0.6, 0}}],
 PlotLegends → Automatic, FrameLabel → Automatic]
```

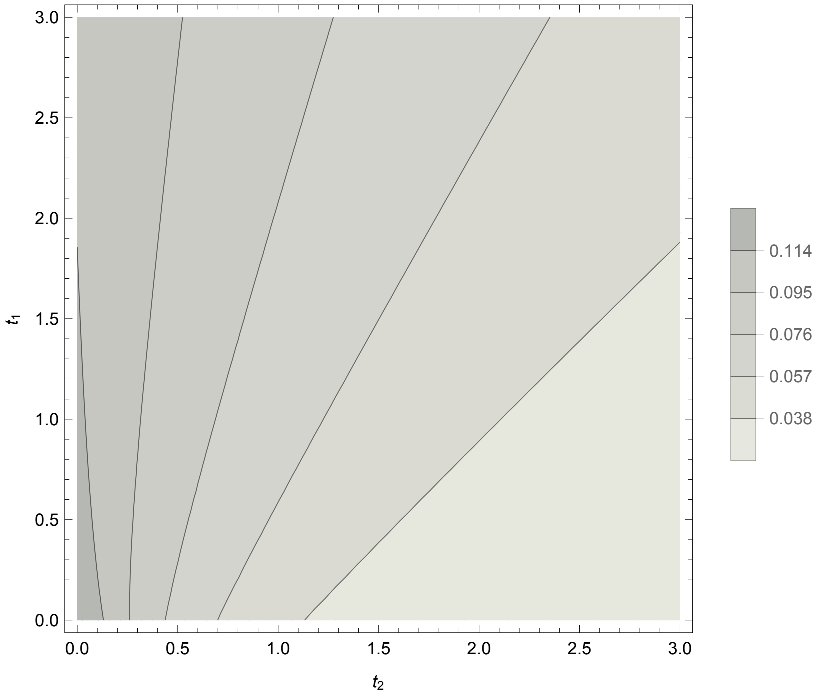


```
S1xS2$AncestralP =
  (Outer[GetBranchProducts$AncOnly[##, S1xS2] &, trees, trees] -
     Outer[CullDerivedAncestralProducts$AncOnly[##, S1xS2] &, trees, trees] +
     Outer[AddDerivedAncestralOrdering$AncOnly[##, S1xS2] &, trees, trees]) *
   S1xS2$TreeMatrix;
```

```
ContourPlot[Total[S1xS2$AncestralP, 2] / Total[S1xS2$PMatrix, 2],
 {t2, 0, 3}, {t1, 0, 3}, PlotLegends → Automatic, FrameLabel → Automatic]
```

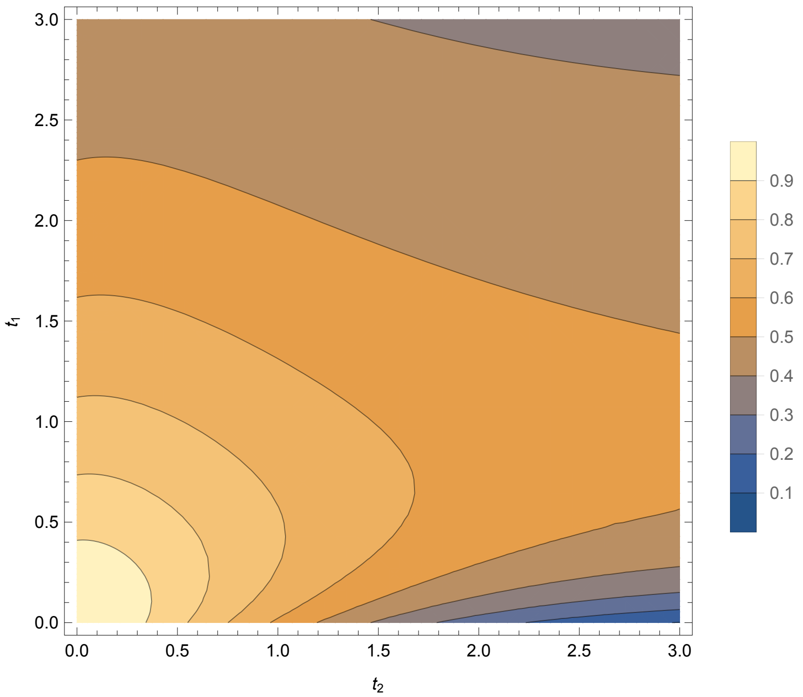


```
S1xS3$AncestralP =
  (Outer[GetBranchProducts$AncOnly[##, S1xS3] &, trees, trees] -
     Outer[CullDerivedAncestralProducts$AncOnly[##, S1xS3] &, trees, trees] +
     Outer[AddDerivedAncestralOrdering$AncOnly[##, S1xS3] &, trees, trees]) *
   S1xS3$TreeMatrix;

S2xS3$AncestralP =
  (Outer[GetBranchProducts$AncOnly[##, S2xS3] &, trees, trees] -
     Outer[CullDerivedAncestralProducts$AncOnly[##, S2xS3] &, trees, trees] +
     Outer[AddDerivedAncestralOrdering$AncOnly[##, S2xS3] &, trees, trees]) *
   S2xS3$TreeMatrix;
```

```
ContourPlot[Total[S1xS3$AncestralP, 2] / Total[S1xS3$PMatrix, 2],
 {t2, 0, 3}, {t1, 0, 3}, PlotLegends → Automatic, FrameLabel → Automatic]
```

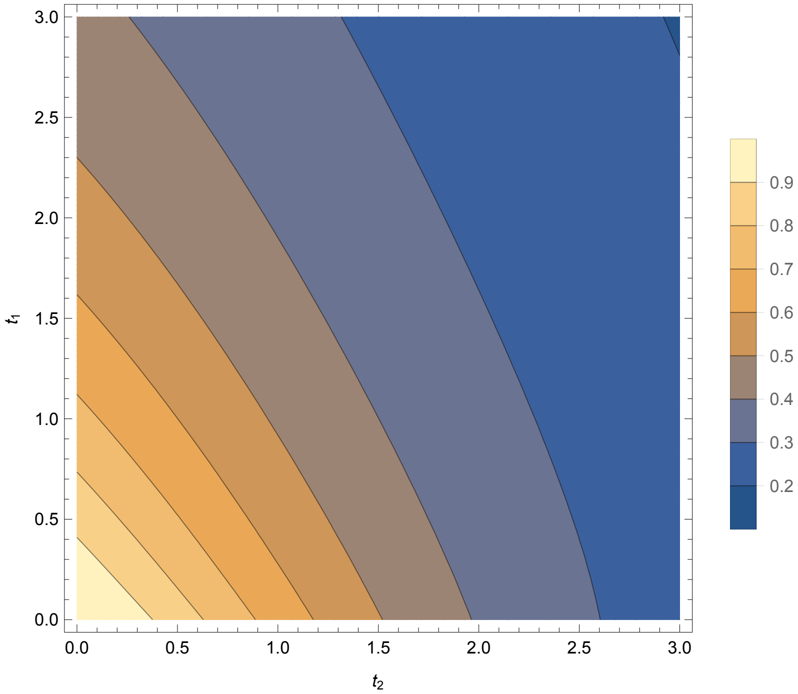


```
S1xS2$S1xS3$AncestralP =
  (Outer[GetBranchProducts$AncOnly[##, S1xS2$S1xS3] &, trees, trees] -
     Outer[CullDerivedAncestralProducts$AncOnly[##, S1xS2$S1xS3] &, trees, trees] +
     Outer[AddDerivedAncestralOrdering$AncOnly[##, S1xS2$S1xS3] &, trees, trees]) *
   S1xS2$S1xS3$TreeMatrix;

S1xS2$S2xS3$AncestralP =
  (Outer[GetBranchProducts$AncOnly[##, S1xS2$S2xS3] &, trees, trees] -
     Outer[CullDerivedAncestralProducts$AncOnly[##, S1xS2$S2xS3] &, trees, trees] +
     Outer[AddDerivedAncestralOrdering$AncOnly[##, S1xS2$S2xS3] &, trees, trees]) *
   S1xS2$S2xS3$TreeMatrix;
```

```
ContourPlot[Total[S1xS2$S1xS3$AncestralP, 2] / Total[S1xS2$S1xS3$PMatrix, 2],
 {t2, 0, 3}, {t1, 0, 3}, PlotLegends → Automatic, FrameLabel → Automatic]
```

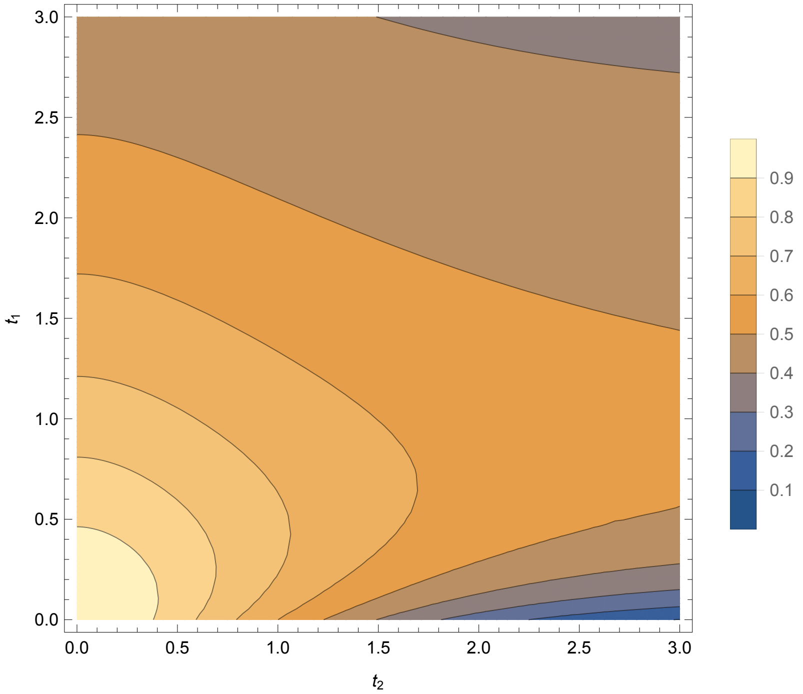


```
S1xS3$S2xS3$AncestralP =
  (Outer[GetBranchProducts$AncOnly[##, S1xS3$S2xS3] &, trees, trees] -
     Outer[CullDerivedAncestralProducts$AncOnly[##, S1xS3$S2xS3] &, trees, trees] +
     Outer[AddDerivedAncestralOrdering$AncOnly[##, S1xS3$S2xS3] &, trees, trees]) *
   S1xS3$S2xS3$TreeMatrix;
```

```
ContourPlot[Total[S1xS3$S2xS3$AncestralP, 2] / Total[S1xS3$S2xS3$PMatrix, 2],
 {t2, 0, 3}, {t1, 0, 3}, PlotLegends → Automatic, FrameLabel → Automatic]
```

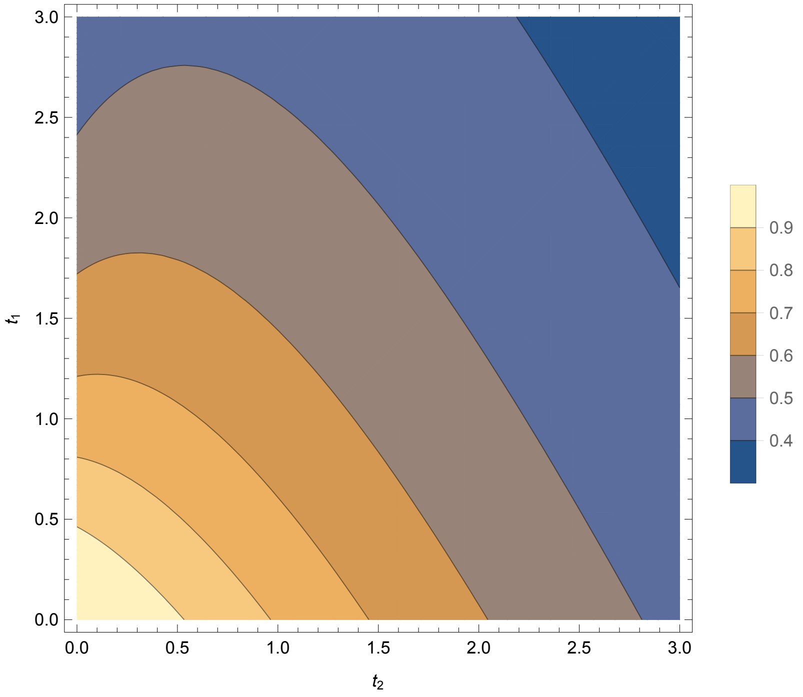


```
P$ancestral = Simplify[(Total[S1xS2$AncestralP, 2] +
       Total[S1xS3$AncestralP, 2] +
       Total[S2xS3$AncestralP, 2] +
       Total[S1xS3$S2xS3$AncestralP, 2] +
       Total[S1xS2$S1xS3$AncestralP, 2] +
       Total[S1xS2$S2xS3$AncestralP, 2]) /
     (Total[S1xS2$PMatrix, 2] + Total[S1xS3$PMatrix, 2] +
       Total[S2xS3$PMatrix, 2] + Total[S1xS3$S2xS3$PMatrix, 2] +
       Total[S1xS2$S1xS3$PMatrix, 2] + Total[S1xS2$S2xS3$PMatrix, 2])];
```

```
ContourPlot[P$ancestral, {t₂, 0, 3}, {t₁, 0, 3},
 ColorFunctionScaling → False, PlotRange → All,
 Contours → {0.3, 0.4, 0.5, 0.6, 0.7, 0.8, 0.9},
 ColorFunction → ColorData[{"GrayTones", {1, 0.3}}],
 PlotLegends → Automatic]
```

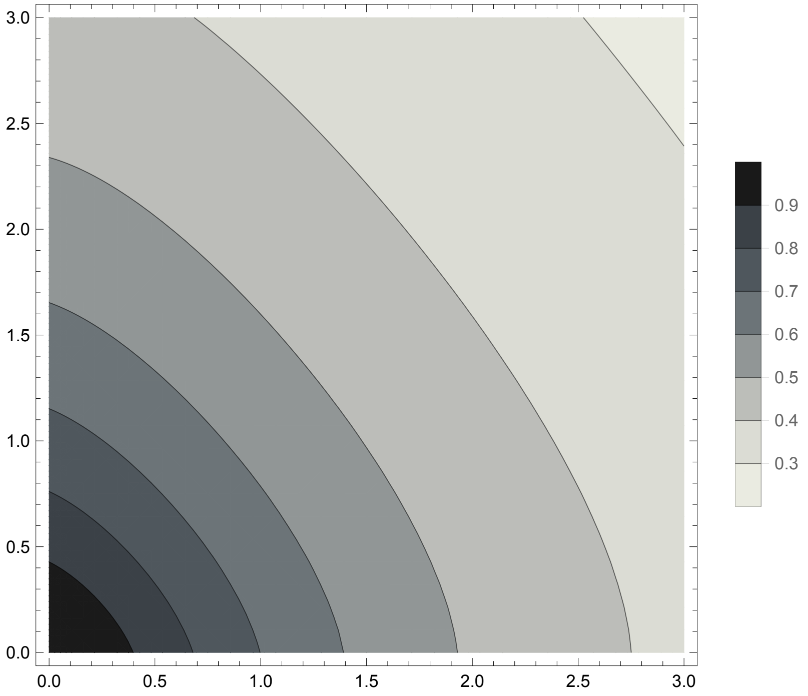

Supplement: Supplementary file 15 [file EVL3-2-281-s014.pdf]
