# Supplementary material for: Speciation genes are more likely to have discordant gene trees
Source: Evol Lett. 2018 Aug 8;2(4):281–96. doi: 10.1002/evl3.77 (PMC6121824; doi:10.1002/evl3.77)

```
Get[NotebookDirectory[] <> "IncompatibilityMatrices.m"];

Get[NotebookDirectory[] <> "RelaxedAncestralMatrices.m"];

BranchLength["(S1,S2)S3 int"] = {t1, q[t2], t1, q[t2], t1, t2, 1, t2 - q[t2], 1};
BranchLength["(S1,S2)S3 anc"] = {t1, t2, 1/3, t1, t2, 1/3, t1, t2, 4/3, 1};
BranchLength["(S2,S3)S1"] = {t1, t2, 4/3, t1, t2, 1/3, t1, t2, 1/3, 1};
BranchLength["(S1,S3)S2"] = {t1, t2, 1/3, t1, t2, 4/3, t1, t2, 1/3, 1};

q[x_] := 1 - (x / (E^x - 1));

ParseMatrix[treematrix_, incompatibility_] :=
 Boole[Map[SameQ[#, incompatibility] &, M[treematrix], {2}]]

GetBranchProducts[tree1_, tree2_, incompatibility_] :=
 If[MatrixQ[M[StringJoin[tree1, ";", tree2]]],
  Simplify[BranchLength[tree1].ParseMatrix[
     StringJoin[tree1, ";", tree2], incompatibility].BranchLength[tree2]],
  Simplify[BranchLength[tree2].ParseMatrix[StringJoin[tree2, ";", tree1],
     incompatibility].BranchLength[tree1]]]

GetAncestralProducts[tree1_, tree2_, incompatibility_] :=
 If[MatrixQ[M[StringJoin[tree1, ";", tree2]]],
  Simplify[BranchLength[tree1].
    (ParseMatrix[StringJoin[tree1, ";", tree2], incompatibility] *
      RDA[StringJoin[tree1, ";", tree2]]).
    BranchLength[tree2]],
  Simplify[BranchLength[tree2].
    (ParseMatrix[StringJoin[tree2, ";", tree1], incompatibility] *
      RDA[StringJoin[tree2, ";", tree1]]).
    BranchLength[tree1]]]

trees = {"(S1,S2)S3 int", "(S1,S2)S3 anc", "(S2,S3)S1", "(S1,S3)S2"};
tree$probabilities = {1 - E^-t2, E^-t2/3, E^-t2/3, E^-t2/3};
```

Calculating the probability of concordance for a locus involved in an S_1 x S_2 incompatibility.

`ParseMatrix` returns the indicator matrix for a specific incompatibility pattern from a general matrix of incompatibilities for a gene tree pair.

`GetBranchProducts` returns the inner product of all branch lengths from a particular pair of trees, filtered by the indicator matrix for that pair from `ParseMatrix`. This corresponds to the probability of two mutations that could give rise to a DMI on a specific gene tree pair.

To get these probabilities for all gene tree pairs, `GetBranchProducts` is applied to each pairwise combination. Here, we use the outer product of the tree vector with itself and apply `GetBranchProducts` to each resulting argument; the result is labeled as the

S1xS2$MutationMatrix.

The probability of each gene tree pair can be obtained by taking the outer product of the tree probability vector with itself; this is labeled here as the S1xS2$TreeMatrix.

P(T_x, T_y | S_1 x S_2) P(T_x) P(T_y), for all T_x and T_y, labeled here as S1xS2$PMatrix, can then be obtained by taking the element-wise product of the S1xS2$MutationMatrix with the S1xS2$TreeMatrix.

The marginal probability of concordance, at a single locus, can then be obtained by summing the rows and columns corresponding to the concordant gene trees from this matrix (rows/columns 1 and 2), and dividing by the sum of all elements in the matrix. The marginal probability of discordance can be similarly obtained (rows/columns 3 and 4). The sum of marginal probabilities for both concordance and discordance is equivalent to the sum of all elements in the matrix, that is, the denominator of Eq. 1 from the main text.

The relative probability of concordance can then be compared to the expected probability of concordance for a random locus: $1 - \frac{2\,e^{-t_2}}{3}$.

```
S1xS2$MutationMatrix = Outer[GetBranchProducts[##, S1xS2] &, trees, trees];
S1xS2$TreeMatrix = Outer[Times, tree$probabilities, tree$probabilities] ;
S1xS2$PMatrix = S1xS2$MutationMatrix * S1xS2$TreeMatrix;

(*
Sanity check:
  the probability of concordance for a random locus should be 1 - (2/3)e^-t,
We should get this by taking the marginal probability of
 concordance from the gene tree probability matrix
*)
FullSimplify[(1/2) ×
  (Total[S1xS2$TreeMatrix[[1]]] + Total[S1xS2$TreeMatrix[[2]]] +
    Total[S1xS2$TreeMatrix[[All, 1]]] + Total[S1xS2$TreeMatrix[[All, 2]]])]
```

$$1 - \frac{2\,e^{-t_2}}{3}$$

```
S1xS2$MarginalConcordant =
  Simplify[(1/2) (Total[S1xS2$PMatrix[[1]]] + Total[S1xS2$PMatrix[[2]]] +
      Total[S1xS2$PMatrix[[All, 1]]] + Total[S1xS2$PMatrix[[All, 2]]])];
S1xS2$MarginalDiscordant =
  Simplify[(1/2) (Total[S1xS2$PMatrix[[3]]] + Total[S1xS2$PMatrix[[4]]] +
      Total[S1xS2$PMatrix[[All, 3]]] + Total[S1xS2$PMatrix[[All, 4]]])];
```

```
S1xS2$ConcordanceRatio = FullSimplify[
  S1xS2$MarginalConcordant / (S1xS2$MarginalConcordant + S1xS2$MarginalDiscordant)]
```

$$1 - \frac{2\, e^{-t_2}\,(7 + 3\,t_1 + 3\,t_2)}{9\,(2 + t_1)}$$

```
ContourPlot[S1xS2$ConcordanceRatio / (1 - 2 e^-t2 / 3),
 {t2, 0, 3}, {t1, 0, 3}, PlotLegends → Automatic,
 Contours → {0.55, 0.65, 0.75, 0.85, 0.95, 1.05, 1.15, 1.25, 1.35, 1.45},
 ColorFunctionScaling → False,
 ColorFunction → ColorData[{"ThermometerColors", {1.45, 0.6}}]]
```

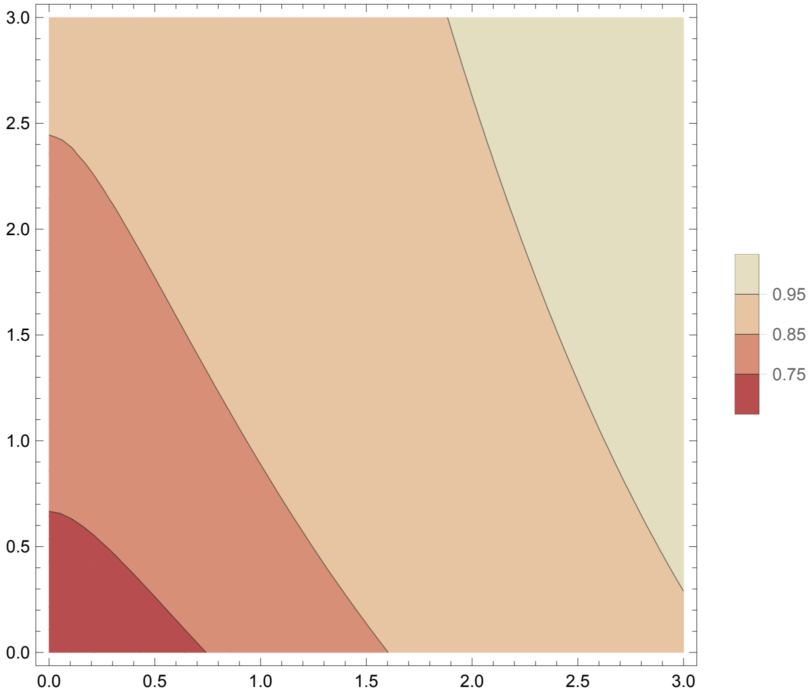

Calculating $P(T_x, T_y \mid S_1 \times S_2; S_1 \times S_3$ incompatibility), labeled here as `S1xS2$S1xS3$PMatrix` and $P(T_x, T_y \mid S_1 \times S_2; S_2 \times S_3$ incompatibility), labeled here as `S1xS2$S2xS3$PMatrix`

```
S1xS2$S1xS3$MutationMatrix =
  Outer[GetBranchProducts[##, S1xS2$S1xS3] &, trees, trees];
S1xS2$S1xS3$TreeMatrix = Outer[Times, tree$probabilities, tree$probabilities] ;
S1xS2$S1xS3$PMatrix = S1xS2$S1xS3$MutationMatrix * S1xS2$S1xS3$TreeMatrix;
```

```
S1xS2$S1xS3$MarginalConcordant =
  Simplify[
   (1/2) (Total[S1xS2$S1xS3$PMatrix[[1]]] + Total[S1xS2$S1xS3$PMatrix[[2]]] +
      Total[S1xS2$S1xS3$PMatrix[[All, 1]]] +
      Total[S1xS2$S1xS3$PMatrix[[All, 2]]])];
S1xS2$S1xS3$MarginalDiscordant =
  Simplify[
   (1/2) (Total[S1xS2$S1xS3$PMatrix[[3]]] + Total[S1xS2$S1xS3$PMatrix[[4]]] +
      Total[S1xS2$S1xS3$PMatrix[[All, 3]]] + Total[S1xS2$S1xS3$PMatrix[[All, 4]]])];

S1xS2$S1xS3$ConcordanceRatio = FullSimplify[S1xS2$S1xS3$MarginalConcordant /
   (S1xS2$S1xS3$MarginalConcordant + S1xS2$S1xS3$MarginalDiscordant)]
```

$$1 - \frac{2\, e^{-t_2} \left(7 + 3\, t_1 + 3\, t_2\right)}{9\, \left(2 + t_1\right)}$$

```
S1xS2$S2xS3$MutationMatrix =
  Outer[GetBranchProducts[##, S1xS2$S2xS3] &, trees, trees];
S1xS2$S2xS3$TreeMatrix = Outer[Times, tree$probabilities, tree$probabilities] ;
S1xS2$S2xS3$PMatrix = S1xS2$S2xS3$MutationMatrix * S1xS2$S2xS3$TreeMatrix;

S1xS2$S2xS3$MarginalConcordant =
  Simplify[
   (1/2) (Total[S1xS2$S2xS3$PMatrix[[1]]] + Total[S1xS2$S2xS3$PMatrix[[2]]] +
      Total[S1xS2$S2xS3$PMatrix[[All, 1]]] +
      Total[S1xS2$S2xS3$PMatrix[[All, 2]]])];
S1xS2$S2xS3$MarginalDiscordant =
  Simplify[
   (1/2) (Total[S1xS2$S2xS3$PMatrix[[3]]] + Total[S1xS2$S2xS3$PMatrix[[4]]] +
      Total[S1xS2$S2xS3$PMatrix[[All, 3]]] + Total[S1xS2$S2xS3$PMatrix[[All, 4]]])];

S1xS2$S2xS3$ConcordanceRatio = FullSimplify[S1xS2$S2xS3$MarginalConcordant /
    (S1xS2$S2xS3$MarginalConcordant + S1xS2$S2xS3$MarginalDiscordant)];

S1xS2$S2xS3$ConcordanceRatio == S1xS2$S1xS3$ConcordanceRatio

True
```

```
ContourPlot[S1xS2$S2xS3$ConcordanceRatio / (1 - 2 e^-t2 / 3),
 {t2, 0, 3}, {t1, 0, 3}, PlotLegends → Automatic,
 Contours → {0.55, 0.65, 0.75, 0.85, 0.95, 1.05, 1.15, 1.25, 1.35, 1.45},
 ColorFunctionScaling → False,
 ColorFunction → ColorData[{"ThermometerColors", {1.45, 0.6}}]]
```

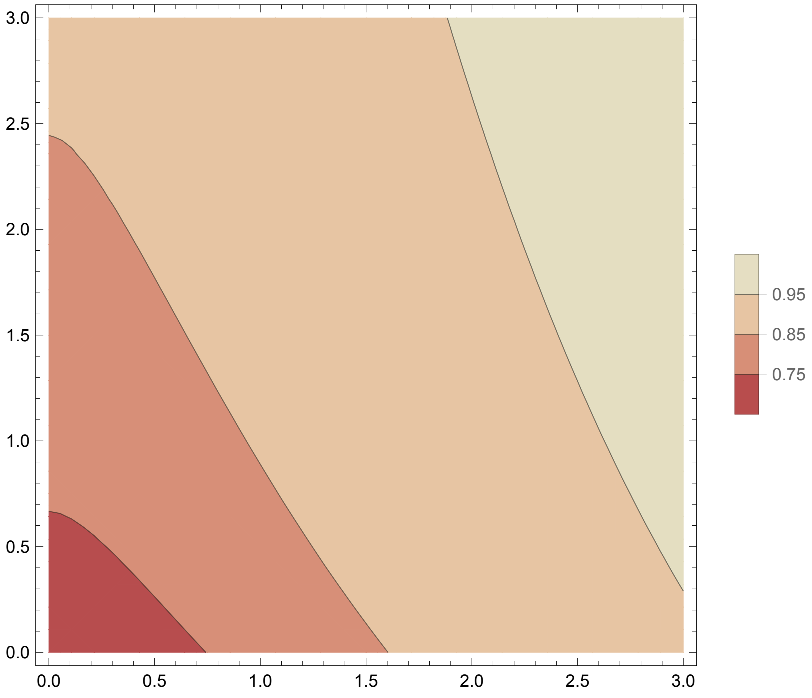

Calculating $P(T_x, T_y \mid S_1 \, x \, S_3)$, labeled here as `S1xS3$PMatrix`

```
S1xS3$MutationMatrix = Outer[GetBranchProducts[##, S1xS3] &, trees, trees];
S1xS3$TreeMatrix = Outer[Times, tree$probabilities, tree$probabilities] ;

fv[x_] = E^-x / (1 - E^-t2);

fτ[t_] = Simplify[Limit[Integrate[fv[x] / (t2 - x), x], x → t] -
    Limit[Integrate[fv[x] / (t2 - x), x], x → 0]];

g[t2_] = FullSimplify[Integrate[t * fv[t] * Integrate[fτ[x], {x, t, t2}], {t, 0, t2}] +
    Integrate[t * fτ[t] * Integrate[fv[x], {x, t, t2}], {t, 0, t2}]];

(*
Changes to the probability of an incompatibility due to mutation
 ordering. Product from lengths of segment dg/eg and gh (also eh/dh and gh)
 on (S1,S2)S3 int. tree subtracted. Replaced with calculations with
 mutation order taken into account (see Supplemental Material).
*)
```

```
{Outer[Times, {1, 0, 0, 0}, {0, 1, 1, 1}] +
   Outer[Times, {0, 1, 1, 1}, {1, 0, 0, 0}] // MatrixForm,
 Outer[Times, {1, 0, 0, 0}, {1, 0, 0, 0}] // MatrixForm}
```

$$\left\{\begin{pmatrix}0&1&1&1\\1&0&0&0\\1&0&0&0\\1&0&0&0\end{pmatrix},\begin{pmatrix}1&0&0&0\\0&0&0&0\\0&0&0&0\\0&0&0&0\end{pmatrix}\right\}$$

```
S1xS3$MutationMatrix += (Outer[Times, {1, 0, 0, 0}, {0, 1, 1, 1}] +
      Outer[Times, {0, 1, 1, 1}, {1, 0, 0, 0}])
    (-(t2 - q[t2]) * t2 + (t2 - q[t2]) ((1/2) (q[t2] + t2)));
S1xS3$MutationMatrix += Outer[Times, {1, 0, 0, 0}, {1, 0, 0, 0}]
    (-2 * (t2 - q[t2]) * q[t2] + 2 * (t2 - q[t2]) * g[t2]);

S1xS3$PMatrix = S1xS3$MutationMatrix * S1xS3$TreeMatrix;

S1xS3$MarginalConcordant =
  Simplify[(1/2) (Total[S1xS3$PMatrix[[1]]] + Total[S1xS3$PMatrix[[2]]] +
      Total[S1xS3$PMatrix[[All, 1]]] + Total[S1xS3$PMatrix[[All, 2]]])];
S1xS3$MarginalDiscordant =
  Simplify[(1/2) (Total[S1xS3$PMatrix[[3]]] + Total[S1xS3$PMatrix[[4]]] +
      Total[S1xS3$PMatrix[[All, 3]]] + Total[S1xS3$PMatrix[[All, 4]]])];

S1xS3$ConcordanceRatio = Simplify[S1xS3$MarginalConcordant /
     (S1xS3$MarginalConcordant + S1xS3$MarginalDiscordant)];

S2xS3$MutationMatrix = Outer[GetBranchProducts[##, S2xS3] &, trees, trees];
S2xS3$TreeMatrix = Outer[Times, tree$probabilities, tree$probabilities] ;

S2xS3$MutationMatrix += (Outer[Times, {1, 0, 0, 0}, {0, 1, 1, 1}] +
      Outer[Times, {0, 1, 1, 1}, {1, 0, 0, 0}])
    (-(t2 - q[t2]) * t2 + (t2 - q[t2]) ((1/2) (q[t2] + t2)));
S2xS3$MutationMatrix += Outer[Times, {1, 0, 0, 0}, {1, 0, 0, 0}]
    (-2 * (t2 - q[t2]) * q[t2] + 2 * (t2 - q[t2]) * g[t2]);

S2xS3$PMatrix = S2xS3$MutationMatrix * S2xS3$TreeMatrix;

S2xS3$MarginalConcordant =
  Simplify[(1/2) (Total[S2xS3$PMatrix[[1]]] + Total[S2xS3$PMatrix[[2]]] +
      Total[S2xS3$PMatrix[[All, 1]]] + Total[S2xS3$PMatrix[[All, 2]]])];
S2xS3$MarginalDiscordant =
  Simplify[(1/2) (Total[S2xS3$PMatrix[[3]]] + Total[S2xS3$PMatrix[[4]]] +
      Total[S2xS3$PMatrix[[All, 3]]] + Total[S2xS3$PMatrix[[All, 4]]])];

S2xS3$ConcordanceRatio = Simplify[S2xS3$MarginalConcordant /
     (S2xS3$MarginalConcordant + S2xS3$MarginalDiscordant)];
```

```
S1xS3$ConcordanceRatio == S2xS3$ConcordanceRatio
```

```
True
```

```
ContourPlot[S1xS3$ConcordanceRatio / (1 - (2 e^-t2)/3),
 {t2, 0, 3}, {t1, 0, 3}, PlotLegends → Automatic, PlotRange → All,
 Contours → {0.55, 0.65, 0.75, 0.85, 0.95, 1, 1.05, 1.15, 1.25, 1.35, 1.45},
 ColorFunctionScaling → False,
 ColorFunction → ColorData[{"ThermometerColors", {1.45, 0.6}}]]
```

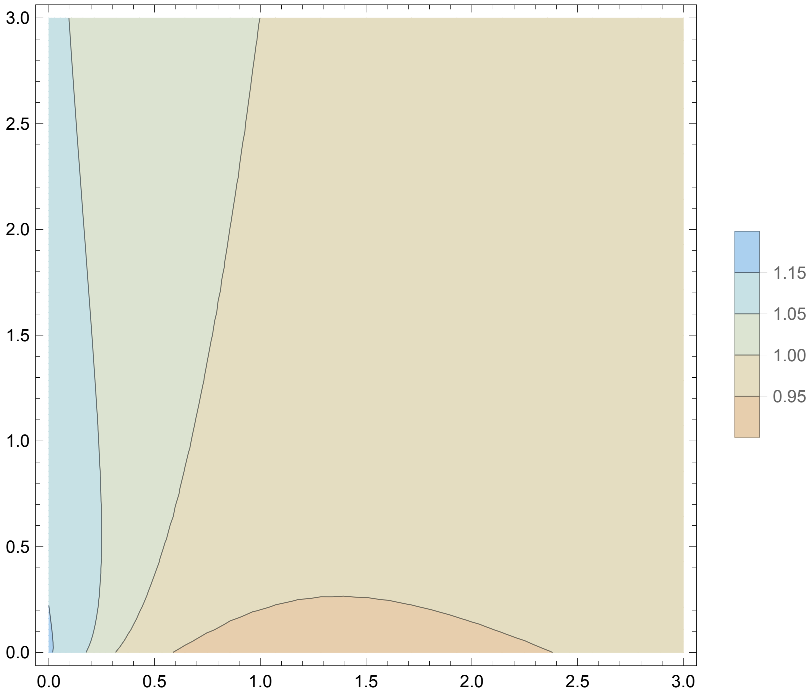

Calculating $P(T_x, T_y \mid S_1 \, x \, S_3; S_2 \, x \, S_3)$, labeled here as `S1xS3$S2xS3$PMatrix` and

```
S1xS3$S2xS3$MutationMatrix =
  Outer[GetBranchProducts[##, S1xS3$S2xS3] &, trees, trees];
S1xS3$S2xS3$TreeMatrix = Outer[Times, tree$probabilities, tree$probabilities] ;
S1xS3$S2xS3$PMatrix = S1xS3$S2xS3$MutationMatrix * S1xS3$S2xS3$TreeMatrix;
```

```
S1xS3$S2xS3$MarginalConcordant =
  Simplify[
   (1/2) (Total[S1xS3$S2xS3$PMatrix[[1]]] + Total[S1xS3$S2xS3$PMatrix[[2]]] +
      Total[S1xS3$S2xS3$PMatrix[[All, 1]]] +
      Total[S1xS3$S2xS3$PMatrix[[All, 2]]])];
S1xS3$S2xS3$MarginalDiscordant =
  Simplify[
   (1/2) (Total[S1xS3$S2xS3$PMatrix[[3]]] + Total[S1xS3$S2xS3$PMatrix[[4]]] +
      Total[S1xS3$S2xS3$PMatrix[[All, 3]]] + Total[S1xS3$S2xS3$PMatrix[[All, 4]]])];
```

```
S1xS3$S2xS3$ConcordanceRatio = Simplify[S1xS3$S2xS3$MarginalConcordant /
    (S1xS3$S2xS3$MarginalConcordant + S1xS3$S2xS3$MarginalDiscordant)];
```

$$\text{ContourPlot}\left[\text{S1xS3\$S2xS3\$ConcordanceRatio} \Big/ \left(1-\frac{2\,e^{-t_2}}{3}\right),\right.$$

```
 {t2, 0, 3}, {t1, 0, 3}, PlotLegends → Automatic, PlotRange → All,
 Contours → {0.55, 0.65, 0.75, 0.85, 0.95, 1.05, 1.15, 1.25, 1.35, 1.45},
 ColorFunctionScaling → False,
 ColorFunction → ColorData[{"ThermometerColors", {1.45, 0.6}}]]
```

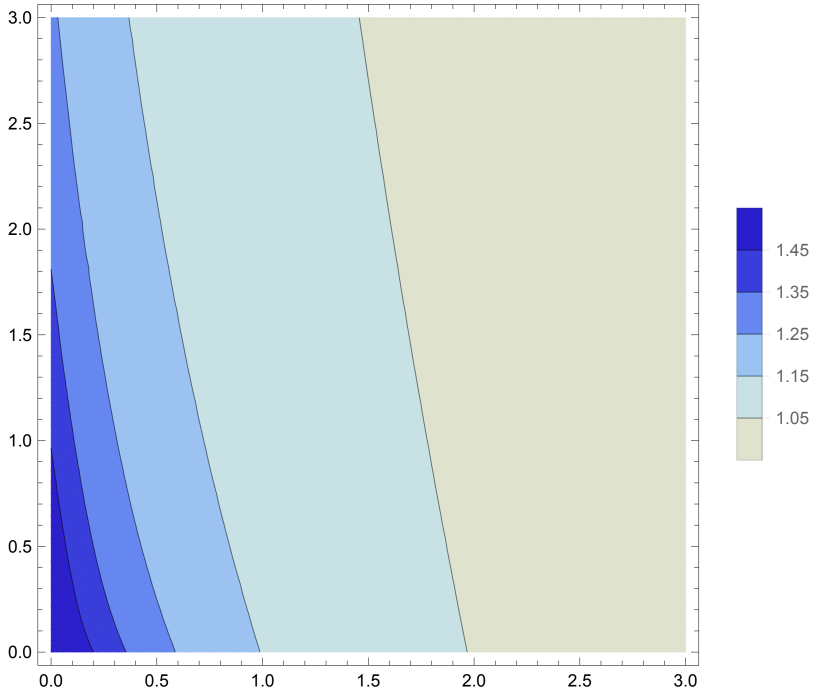


```
P$concord = Simplify[(S1xS2$ConcordanceRatio * Total[S1xS2$PMatrix, 2] +
        S1xS3$ConcordanceRatio * Total[S1xS3$PMatrix, 2] +
        S2xS3$ConcordanceRatio * Total[S2xS3$PMatrix, 2] +
        S1xS3$S2xS3$ConcordanceRatio * Total[S1xS3$S2xS3$PMatrix, 2] +
        S1xS2$S1xS3$ConcordanceRatio * Total[S1xS2$S1xS3$PMatrix, 2] +
        S1xS2$S2xS3$ConcordanceRatio * Total[S1xS2$S2xS3$PMatrix, 2]) /
    (Total[S1xS2$PMatrix, 2] + Total[S1xS3$PMatrix, 2] +
        Total[S2xS3$PMatrix, 2] + Total[S1xS3$S2xS3$PMatrix, 2] +
        Total[S1xS2$S1xS3$PMatrix, 2] + Total[S1xS2$S2xS3$PMatrix, 2])];
```

```
ContourPlot[P$concord / (1 - 2 ⅇ^(-t₂) / 3), {t₂, 0, 3}, {t₁, 0, 3}, PlotLegends → Automatic,
 ColorFunction → ColorData[{"RedBlueTones", {1.05, 0.95}}],
 PlotRange → All, ColorFunctionScaling → False,
 Contours → {0.94, 0.95, 0.96, 0.97,  0.98, 0.99, 1.00, 1.01, 1.02, 1.03, 1.04, 1.05}]
```

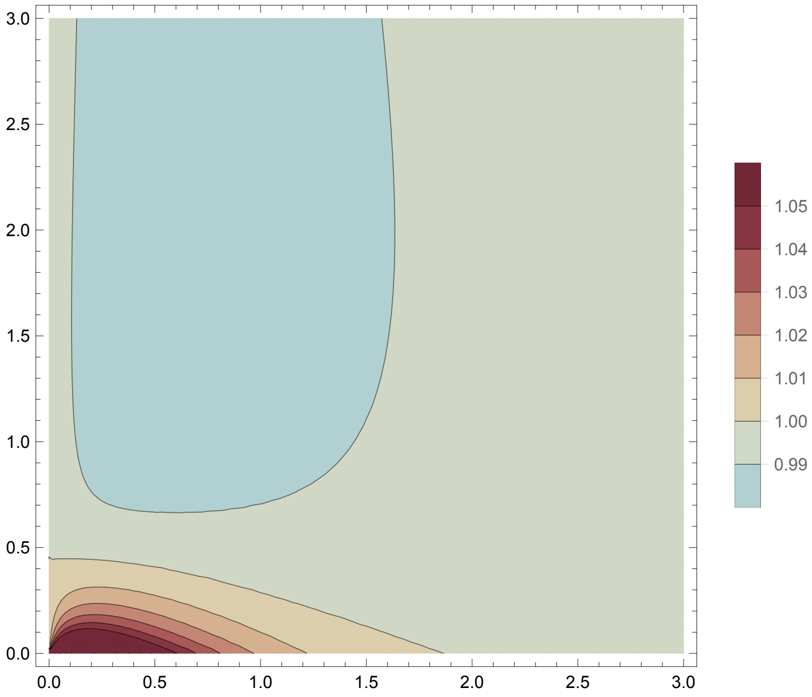


```
TotalP = Total[S1xS2$PMatrix, 2] + Total[S1xS3$PMatrix, 2] +
   Total[S2xS3$PMatrix, 2] + Total[S1xS3$S2xS3$PMatrix, 2] +
   Total[S1xS2$S1xS3$PMatrix, 2] + Total[S1xS2$S2xS3$PMatrix, 2];
```

```
ContourPlot[(Total[S1xS2$PMatrix, 2]) / TotalP, {t2, 0, 5}, {t1, 0, 5},
 ColorFunctionScaling → False,
 Contours → {0.05, 0.1, 0.15, 0.2, 0.25, 0.3, 0.35, 0.4, 0.45, 0.5},
 ColorFunction → ColorData[{"GrayTones", {0.5, 0}}]]
```

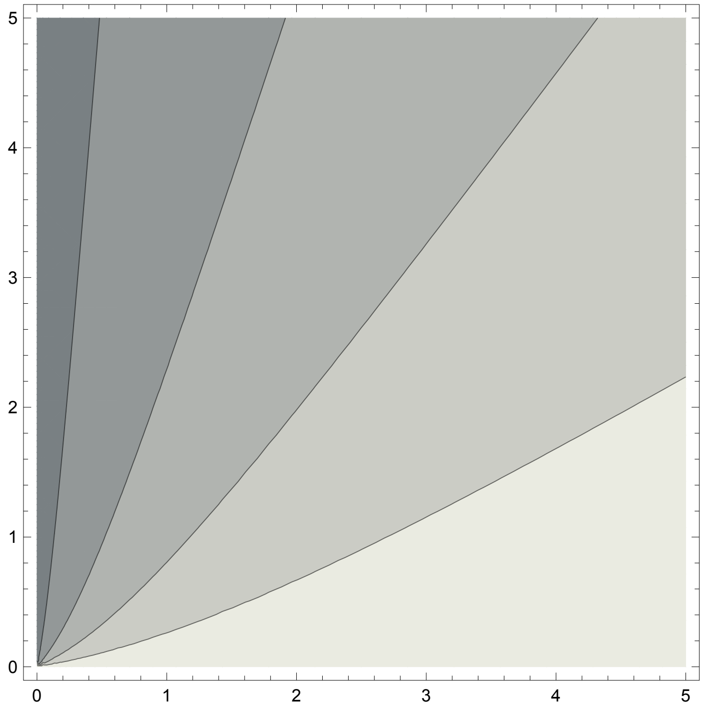

```
ContourPlot[(Total[S1xS3$PMatrix, 2]) / TotalP, {t₂, 0, 5}, {t₁, 0, 5},
 ColorFunctionScaling → False, PlotRange → All,
 Contours → {0.05, 0.1, 0.15, 0.2, 0.25, 0.3, 0.35, 0.4, 0.45, 0.5},
 ColorFunction → ColorData[{"GrayTones", {0.5, 0}}],
 PlotLegends → Automatic]
```

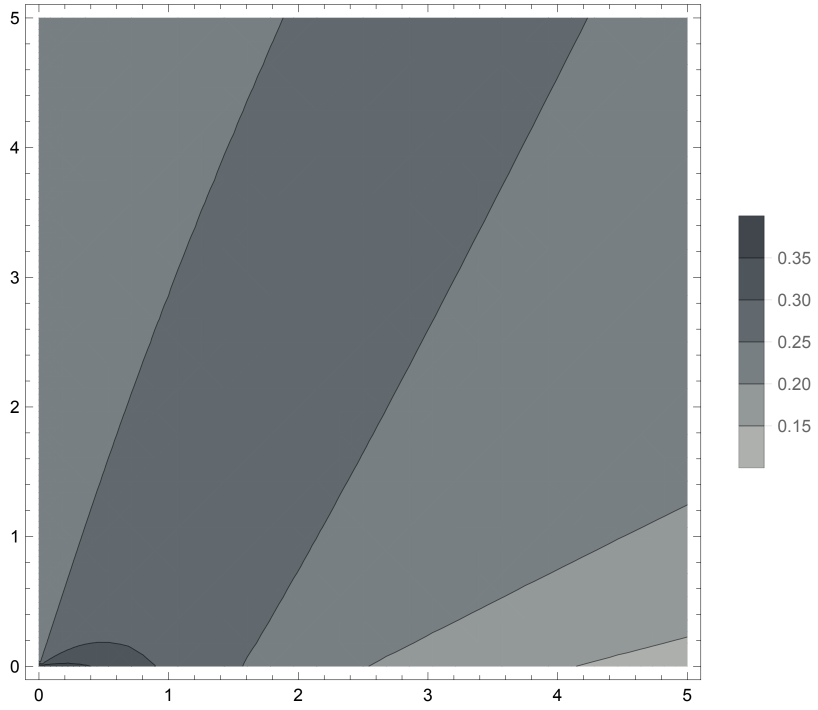

```
ContourPlot[(Total[S1xS3$S2xS3$PMatrix, 2]) / TotalP, {t2, 0, 5}, {t1, 0, 5},
 ColorFunctionScaling → False, PlotRange → All,
 Contours → {0.05, 0.1, 0.15, 0.2, 0.25, 0.3, 0.35, 0.4, 0.45, 0.5},
 ColorFunction → ColorData[{"GrayTones", {0.5, 0}}],
 PlotLegends → Automatic]
```

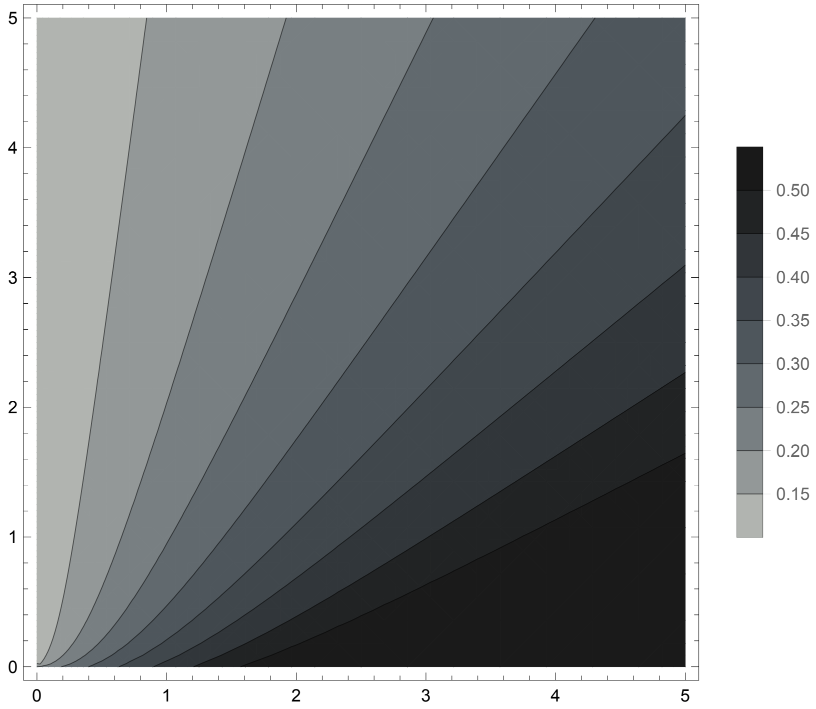

```
ContourPlot[(Total[S1xS2$S2xS3$PMatrix, 2]) / TotalP, {t2, 0, 5}, {t1, 0, 5},
 ColorFunctionScaling → False, PlotRange → All,
 Contours → {0.05, 0.1, 0.15, 0.2, 0.25, 0.3, 0.35, 0.4, 0.45, 0.5},
 ColorFunction → ColorData[{"GrayTones", {0.5, 0}}],
 PlotLegends → Automatic]
```

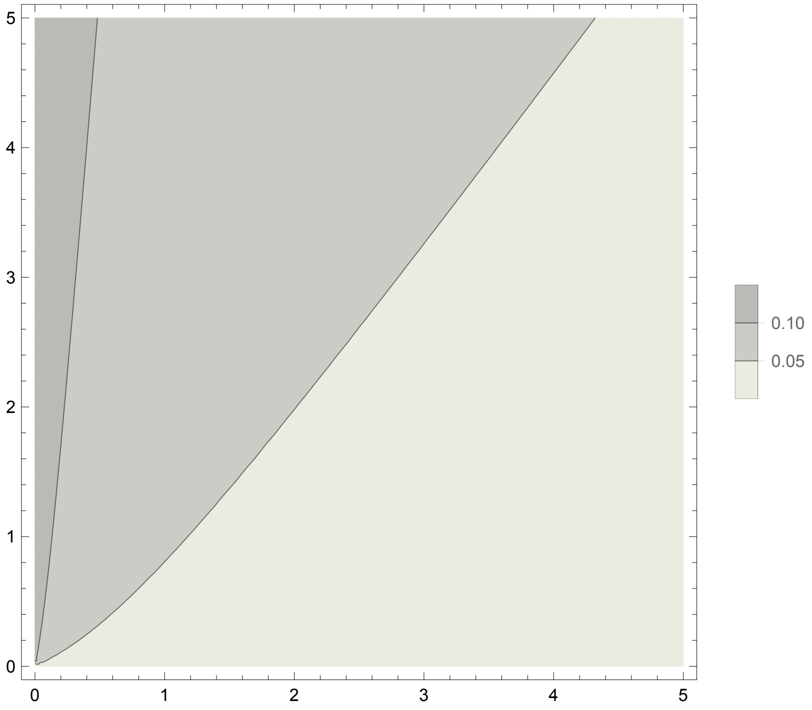


```
S1xS2$AncestralP =
  Outer[GetAncestralProducts[##, S1xS2] &, trees, trees] * S1xS2$TreeMatrix;
```

```
ContourPlot[Total[S1xS2$AncestralP, 2] / Total[S1xS2$PMatrix, 2],
 {t2, 0, 5}, {t1, 0, 5}, ColorFunctionScaling → False, PlotRange → All,
 Contours → {0.2, 0.3, 0.4, 0.5, 0.6, 0.7, 0.8, 0.9},
 ColorFunction → ColorData[{"GrayTones", {1, 0.1}}],
 PlotLegends → Automatic]
```

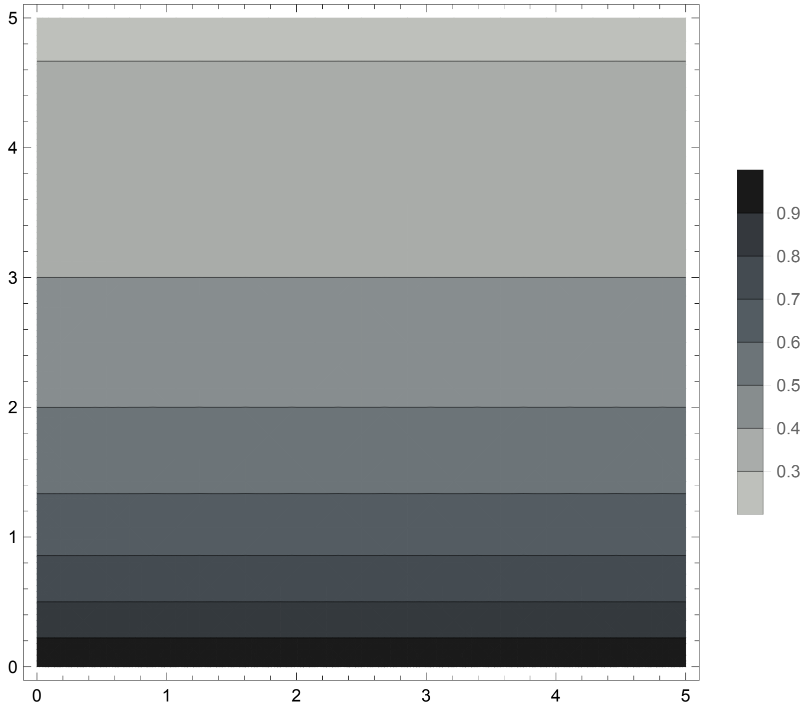


```
S1xS3$AncestralP =
  Outer[GetAncestralProducts[##, S1xS3] &, trees, trees] * S1xS3$TreeMatrix;
```

```
S2xS3$AncestralP =
  Outer[GetAncestralProducts[##, S2xS3] &, trees, trees] * S1xS3$TreeMatrix;
```

```
ContourPlot[Total[S1xS3$AncestralP, 2] / Total[S1xS3$PMatrix, 2],
 {t2, 0, 5}, {t1, 0, 5}, ColorFunctionScaling → False, PlotRange → All,
 Contours → {0.2, 0.3, 0.4, 0.5, 0.6, 0.7, 0.8, 0.9},
 ColorFunction → ColorData[{"GrayTones", {1, 0.1}}],
 PlotLegends → Automatic]
```

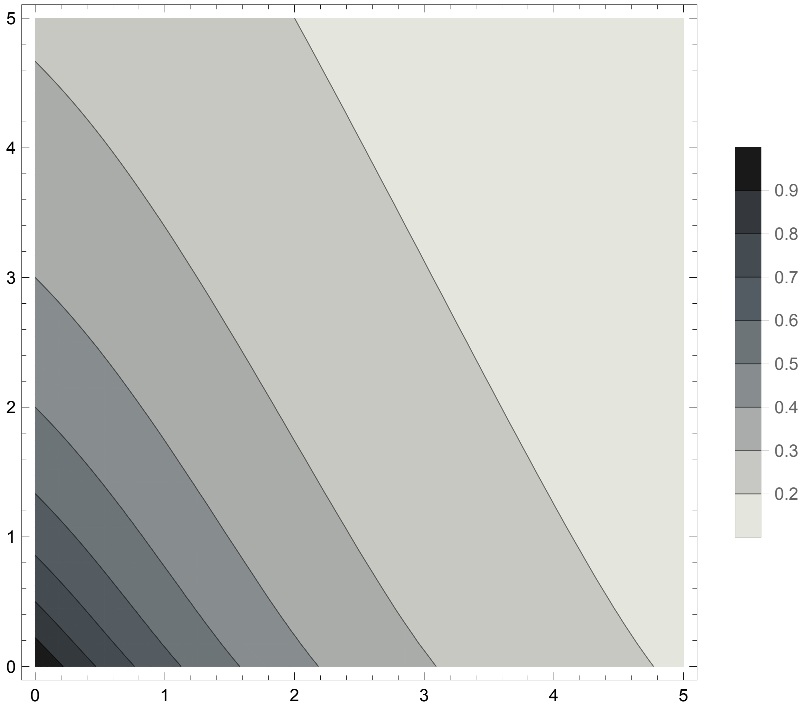


```
S1xS2$S1xS3$AncestralP =
  Outer[GetAncestralProducts[##, S1xS2$S1xS3] &, trees, trees] * S1xS3$TreeMatrix;

S1xS2$S2xS3$AncestralP =
  Outer[GetAncestralProducts[##, S1xS2$S1xS3] &, trees, trees] * S1xS3$TreeMatrix;
```

```
ContourPlot[Total[S1xS2$S1xS3$AncestralP, 2] / Total[S1xS2$S1xS3$PMatrix, 2],
 {t2, 0, 5}, {t1, 0, 5}, ColorFunctionScaling → False, PlotRange → All,
 Contours → {0.2, 0.3, 0.4, 0.5, 0.6, 0.7, 0.8, 0.9},
 ColorFunction → ColorData[{"GrayTones", {1, 0.1}}],
 PlotLegends → Automatic]
```

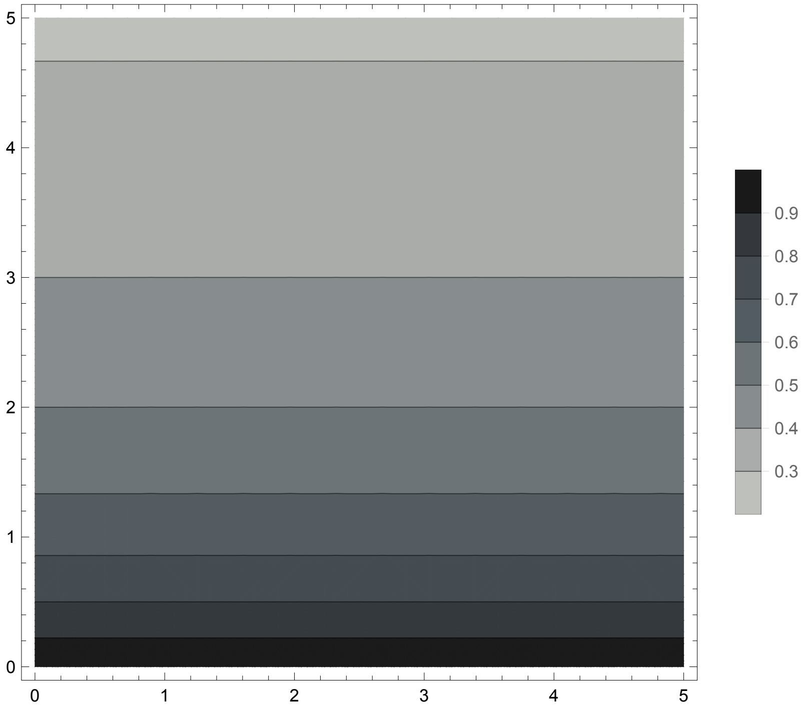


```
S1xS3$S2xS3$AncestralP =
  Outer[GetAncestralProducts[##, S1xS3$S2xS3] &, trees, trees] * S1xS3$TreeMatrix;
```

```
ContourPlot[Total[S1xS3$S2xS3$AncestralP, 2] / Total[S1xS3$S2xS3$PMatrix, 2],
 {t2, 0, 5}, {t1, 0, 5}, ColorFunctionScaling → False, PlotRange → All,
 Contours → {0.2, 0.3, 0.4, 0.5, 0.6, 0.7, 0.8, 0.9},
 ColorFunction → ColorData[{"GrayTones", {1, 0.1}}],
 PlotLegends → Automatic]
```

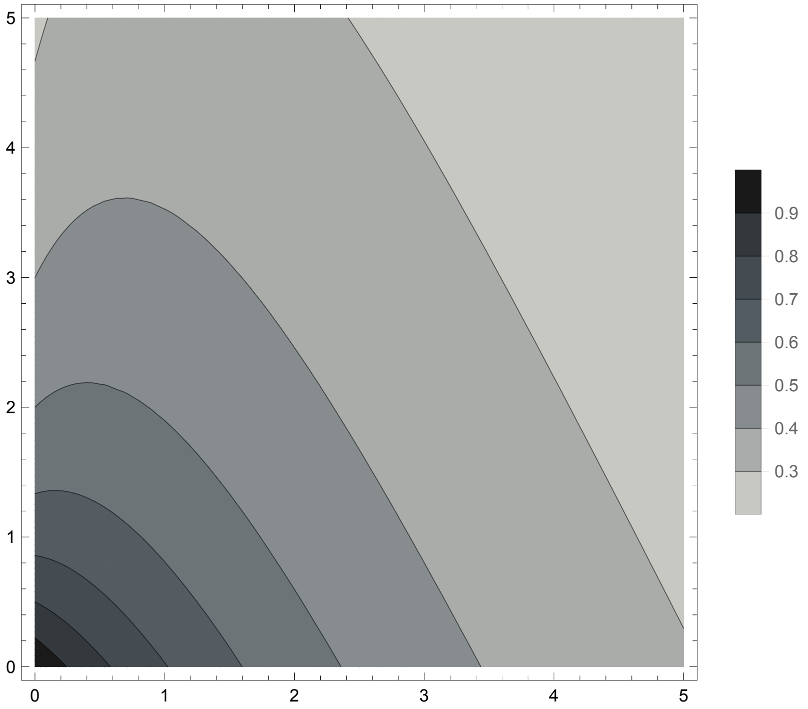


```
P$ancestral = Simplify[(Total[S1xS2$AncestralP, 2] +
       Total[S1xS3$AncestralP, 2] +
       Total[S2xS3$AncestralP, 2] +
       Total[S1xS3$S2xS3$AncestralP, 2] +
       Total[S1xS2$S1xS3$AncestralP, 2] +
       Total[S1xS2$S2xS3$AncestralP, 2]) /
    (Total[S1xS2$PMatrix, 2] + Total[S1xS3$PMatrix, 2] +
       Total[S2xS3$PMatrix, 2] + Total[S1xS3$S2xS3$PMatrix, 2] +
       Total[S1xS2$S1xS3$PMatrix, 2] + Total[S1xS2$S2xS3$PMatrix, 2])];
```

```
ContourPlot[P$ancestral, {t2, 0, 3}, {t1, 0, 3},
 ColorFunctionScaling → False, PlotRange → All,
 Contours → {0.2, 0.3, 0.4, 0.5, 0.6, 0.7, 0.8, 0.9},
 ColorFunction → ColorData[{"GrayTones", {1, 0.1}}],
 PlotLegends → Automatic]
```

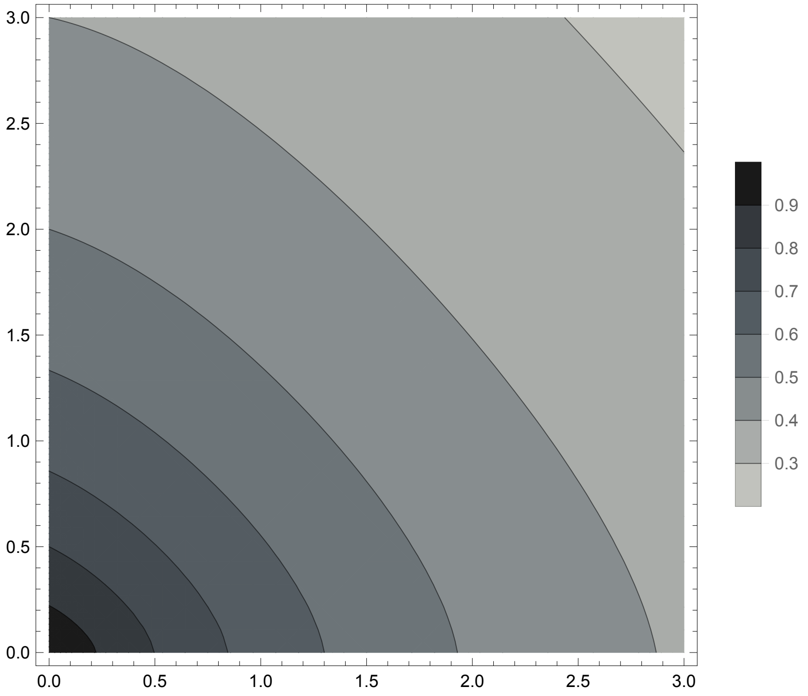

Supplement: Supplementary file 16 [file EVL3-2-281-s015.pdf]
